# Sustainable and Scalable Redesign of PS-750‑M Synthesis While Retaining Micellar Catalytic Efficiency

**DOI:** 10.1021/acssuschemeng.5c13808

**Published:** 2026-02-23

**Authors:** Ramesh Hiralal Choudhary, Amna Akram, Reda Zainab, Ashik Chhetri, Pritam Dolui, Fabrice Gallou, Michael Harmata, Sachin Handa

**Affiliations:** † Department of Chemistry, 601 S College Ave, 14716 University of Missouri, Columbia, Missouri 65211, United States; ‡ Chemical & Analytical Development, 111826 Novartis Pharma AG, 4056 Basel, Switzerland

**Keywords:** PS-750-M, Micellar Catalysis, Process Mass Intensity, E-factor, Cross-Couplings

## Abstract

An efficient, two-step synthetic route for PS-750-M has been developed, offering significant improvements in terms of sustainability, scalability, and operational simplicity compared to the conventional four-step process. The new methodology eliminates hazardous reagents, toxic solvents, and chromatography, while enabling solvent recovery and catalyst reuse. Quantitative green metrics reveal dramatic reductions in environmental impact, with E-factor and process mass intensity (PMI) decreasing by over 90% and 85%, respectively. Importantly, PS-750-M, synthesized via this greener route, retains its micellar catalytic efficiency tested on a variety of transformations, including palladium-catalyzed C–C couplings, Buchwald–Hartwig aminations, biaryl ketone formation, and rapid amide couplings. These results support the industrial viability of the redesigned process and its alignment with the principles of green chemistry, potentially facilitating the large-scale adoption of this approach in pharmaceutical and fine chemical manufacturing.

## Graphical abstract

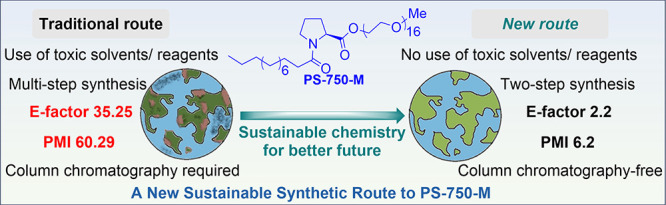

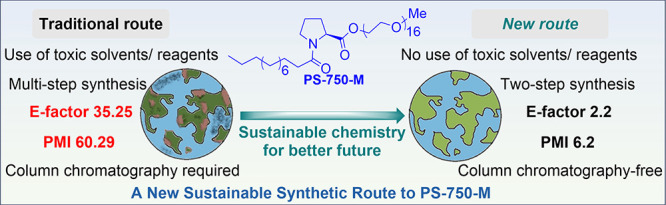

## Introduction

Designing catalytic processes that are both sustainable and scalable has evolved from an aspirational concept to an operational imperative across the fine chemicals and pharmaceutical manufacturing sectors. A central driver of this transition is the recognition that solvents dominate the mass of most chemical processes, and therefore, the waste, as measured in mass-based metrics—the E-factor (kg waste per kg product) and process mass intensity (PMI) (total input mass per kg product).^1^ Reducing solvent usage, switching to safer reaction media, and simplifying synthetic processes are now primary levers for lowering E-factor and PMI, and thus, the environmental footprint of synthesis. For bulk chemicals, acceptable E-factors are typically <1–5, whereas fine chemicals and pharmaceuticals often begin with values higher, reflecting heavy solvent use and multistep workups; lowering these numbers requires process intensification and solvent innovation.^2–4^ PMI complements the E-factor by accounting for all materials (including benign ones and solvents used in product purification), making it particularly useful at the process design stage and for cross-process comparisons.^5^

As Sheldon memorably underscored, *“the best solvent is no solvent, but if needed, water has a lot to offer*.^3^ *”* This aphorism distills a modern process philosophy: leverage solvent-free or minimal-solvent strategies when feasible, and otherwise use water strategically to achieve dramatic improvements in greenness without compromising performance.^6–11^ However, moving complex organic reactions into aqueous media has historically been challenging because most substrates, catalysts, and intermediates are insoluble in water or sensitive to it. The workaround that has unlocked water’s potential is micellar catalysis, performing reactions in water containing a small amount of a designer nonionic surfactant that self-assembles into nanomicelles.^12,13^ These micelles provide lipophilic microenvironments that accommodate insoluble organic reactants and catalysts, thereby enabling chemoselective transition-metal-catalyzed reactions under benign conditions. The micellar cores act as dynamic “pockets,” analogous to enzyme active sites, and can simultaneously serve as solvents, ligands, and reaction promoters, often enabling lower catalyst loadings and shorter reaction times than with classical organic solvents.^14^ Reactions assisted by the right amphiphile can deliver reaction scope, rates, and clean impurity profiles comparable to or better than those obtained in traditional organic solvents.^15^

Beyond academic demonstrations, industrial case studies show that micellar catalysis lowers PMI, reduces cycle time, and simplifies workups (e.g., by replacing column chromatography with phase separations), strengthening the case for scale-up in process chemistry.^16^ These favorable outcomes are attributed to high effective molarity inside micelles, improved mass transfer, and the possibility to recycle water/surfactant and, in several cases, the metal catalyst, thereby addressing both environmental and economic drivers for adoption.^17–19^

Within this landscape, PS-750-M, a proline-based nonionic amphiphile, has emerged as an enabling surfactant for ligand-free catalysis and highly important transformations in water.^20^ Reports highlight completely organic-solvent-free amide couplings, C–H fluorination, and cross-couplings of water-sensitive acyl chlorides, facilitated by nanomicelles of PS-750-M, where the micelle’s hydrophobic interior and interfacial chemistry enable reaction pathways that are otherwise inaccessible or inefficient in bulk water.^21–23^ Mechanistic studies unraveled metal–micelle interactions and shielding effects that stabilize transient species and promote turnover.^24,25^ These properties potentially position PS-750-M as a complementary tool to widely used nonionic surfactants (e.g., TPGS-750-M), broadening the scope of micellar catalysis in water.^26^

Despite these advances, the synthesis and supply of some nonionic surfactants used for micellar catalysis present practical challenges that can undermine sustainability, robustness, and scalability.^27^ Classical routes to amphiphiles, such as TPGS-750-M, involve esterifications between α-tocopherol, succinic acid, and mPEG-750-M.^28^ In contrast, the amphiphile Savie requires toxic solvents and pyrophoric reagents. Each step can require dry organic solvents, activating agents, and purifications that inflate E-factor and PMI, if not engineered for solvent minimization and continuous flow.^29^ This affects process metrics: solvent-heavy syntheses raise E-factor/PMI, variability compromises scale-up reproducibility, and limited recyclability increases operational costs and waste streams, which is likely true for the traditional synthesis of PS-750-M. Addressing them is therefore central to realizing the full sustainability promise of micellar catalysis.

Herein, we address these challenges by introducing a novel two-step synthetic route to PS-750-M, which eliminates crystallization and column chromatography, and minimizes the use of hazardous organic solvents, aligning with Sheldon’s call to reduce solvent burdens and leverage water wherever practical (Scheme 1A). PS-750-M mimics the structural and solvation properties of amidic solvents such as DMF, DMAc, and NMP, enabling a variety of cross-couplings. When surfactant synthesis itself becomes greener and simpler, it can lead to several significant benefits, including lower solvent volumes, fewer unit operations, and streamlined purifications, resulting in dramatically reduced mass intensity. From a process design perspective, such improvements move PS-750-M manufacture closer to fine-chemicals targets and support broader adoption in multikilogram campaigns.^30^ A concise, chromatography-free route reduces batch variability and enables continuous or semicontinuous production, all of which are prerequisites for cGMP integration and industrial supply reliability.^31^ By retaining or enhancing micellar catalytic efficiency, the redesigned PS-750-M synthesis strengthens technology readiness for micellar reactions.

## Results and Discussion

### Synthesis of PS-750-M

The conventional four-step synthetic route^29^ for PS-750-M was successfully redesigned into a streamlined, rapid, and environmentally benign two-step process (Scheme 1B vs Scheme 1C). This method simplifies synthesis while improving sustainability. l-Proline (**1a**) was deprotonated in aqueous sodium hydroxide, followed by the controlled, dropwise addition of lauroyl chloride at 0 °C, which led to the formation of **1b** after the acidic workup. Upon complete consumption of the starting material (approximately 2 h; see Supporting Information, pp S3, S4), the reaction medium was acidified, and extraction with ethyl acetate furnished the *N*-acylated proline intermediate (**1b**) in 95% yield. The intermediate **1b** underwent esterification with poly­(ethylene glycol) methyl ether (mPEG, average MW 750) using Amberlyst-15­(H) as a heterogeneous acid catalyst under toluene reflux for 48 h (for details, see Supporting Information, pp S4, S5). Postreaction workup involved catalyst filtration and toluene recovery for reuse, affording PS-750-M as a white, wax-like surfactant in 98% yield (Scheme 1C).

**Scheme 1:**
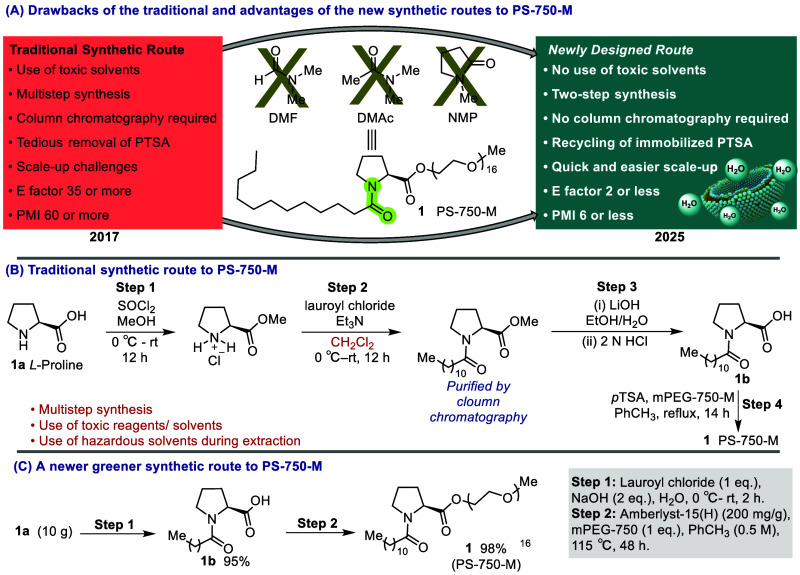
(A) Overview of the Traditional and Advanced Synthetic Routes to PS-750-M (B) Traditional Route versus and (C) Newer Sustainable Synthesis of PS-750-M

In contrast, the previously reported protocol required four steps—methyl ester formation of l-proline, *N*-acylation, ester hydrolysis, and mPEG-750 esterification—employing hazardous reagents (e.g., SOCl_2_) and toxic solvents (e.g., dichloromethane), alongside labor-intensive column chromatography.^29^ These limitations compromise both safety and sustainability. Our redesigned route eliminates these drawbacks by using greener solvents, operating under chromatography-free conditions, and employing recyclable catalysts.

### Alignment with Green Chemistry Principles

The optimized process adheres to 6 out of the 12 Principles of Green Chemistry^32^ by (Figure 1, highlighted in green boxes):

- Reducing the number of synthetic steps (principle 8)
- Eliminating column chromatography (principle 1)
- Avoiding toxic reagents and hazardous solvents, replacing them with ethyl acetate (principles 5 and 12)
- Demonstrating scalability and operational simplicity (principle 4)
- Substituting homogeneous *p*-toluenesulfonic acid with Amberlyst-15­(H) for easy separation and reuse (principle 2)
- Recovering and recycling both catalyst and solvent, minimizing waste and cost (principle 1)

**Figure 1:**
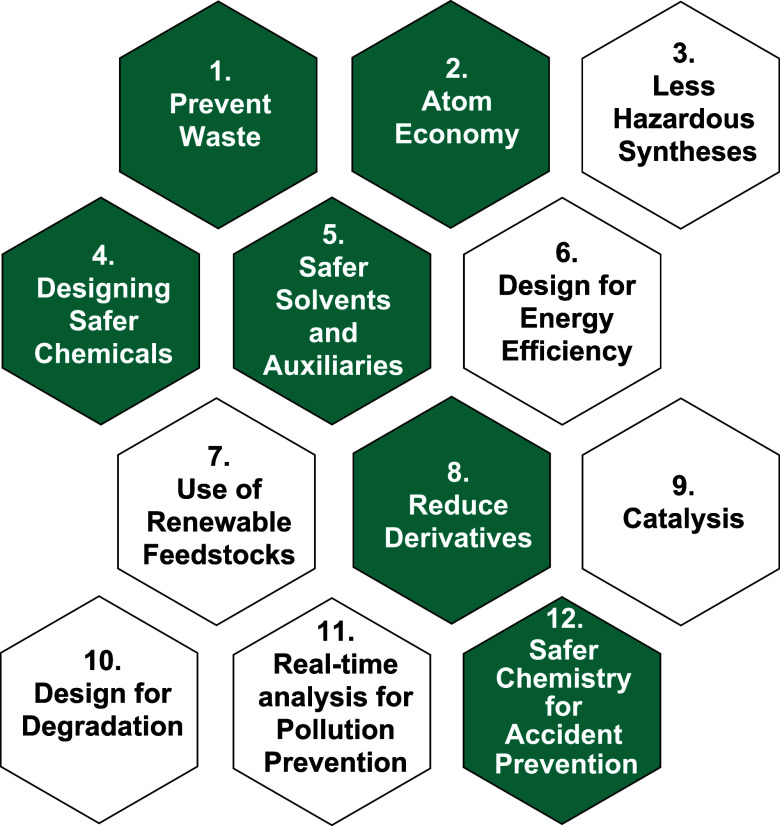
Twelve principles of green chemistry.

Collectively, these improvements render the synthesis highly sustainable, cost-effective, and industrially viable, while preserving excellent yields and product quality.

### Green Metrics

To quantitatively assess the efficiency and environmental impact of both the conventional and optimized synthetic routes, two widely recognized sustainability indicators were employed: the E-factor^33^ and PMI^34^ as well as atom economy and reaction mass efficiency (Figure 2; detailed calculations are provided in the Supporting Information, pp S16–S24). These metrics provide a holistic measure of waste generation and material efficiency, respectively.

**Figure 2:**
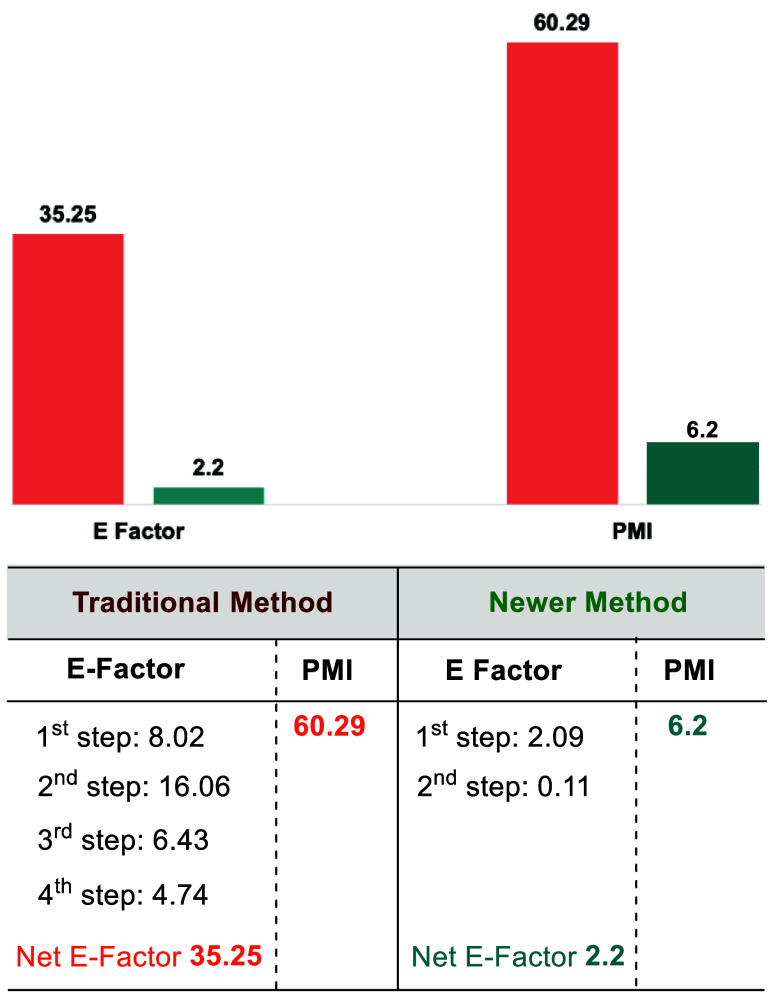
Metrics for comparing sustainability in terms of environmental impact.

For the traditional four-step synthesis, the overall E-factor was calculated to be **35.25**, indicating a process with high waste intensity. Among the individual steps, Step 2 (*N*-acylation) emerged as the most waste-generating, with an E-factor of 16.06. Notably, in accordance with standard definitions, solvents used during column chromatography were excluded from these calculations. Nevertheless, the omission underscores a critical limitation: in practical scenarios, especially when handled by less experienced chemists/graduate students, the actual waste footprint could be significantly higher.

Similarly, the overall PMI for the traditional route was determined to be **60.29**, reflecting substantial material consumption even under optimized laboratory conditions. Again, these numbers assume minimal solvent use during chromatographic purification; in routine practice, this value could escalate considerably.

In stark contrast, the redesigned two-step methodology demonstrated exceptional improvements in sustainability. The E-factor dropped dramatically to **2.2**, a value consistently observed regardless of whether the synthesis was performed by an experienced graduate student or a first-year trainee (see Supporting Information, pp S16–S19). Likewise, the overall PMI was reduced to **6.2**, a multifold decrease compared to the conventional route (see Supporting Information, pp S20, S21). These results unequivocally validate the green design principles embedded in the new synthetic strategy.

Thus, the optimized route achieved a 16-fold reduction in E-factor and an *ca*. 10-fold reduction in PMI, supporting its superior material efficiency and minimal waste generation. Robust reproducibility across varying skill levels (*from an untrained first-year graduate student to a third-year student*) highlights the practicality and scalability of the process. Collectively, these metrics confirm that the new methodology is not only greener but also operationally simpler and economically advantageous.

## Activity Comparisons

The efficiency of PS-750-M synthesized via the new route was assessed and benchmarked against previously reported methods. To evaluate its catalytic performance, a broad range of transformations—including carboxylation,^25^ nitrile arylation,^35^ Suzuki–Miyaura coupling,^23^ oxidative Mizoroki–Heck reaction,^20^ Buchwald–Hartwig amination,^36^ biaryl ketone synthesis,^24^ and amide bond formation^21^—were selected.

### Palladium-Catalyzed Cross Couplings

Our group previously reported the role of PS-750-M in stabilizing the highly reactive trichloromethyl carbanion generated from chloroform and its application in Pd-catalyzed carboxylation of aryl and (hetero)­aryl halides.^25^ The optimized conditions were strictly followed for carboxylation using 3 wt % aqueous PS-750-M synthesized via the new route as the reaction medium. Representative substrates bearing electron-donating methoxy (**5**), pyridyl (**3**), fused carbocycle (**6**), and nitrile (**4**) functionalities, which are challenging to carboxylate under traditional conditions (e.g., using alkyllithiums or Grignard reagents due to side reactions), were successfully converted with yields comparable to those previously reported (Scheme 2A).

**Scheme 2:**
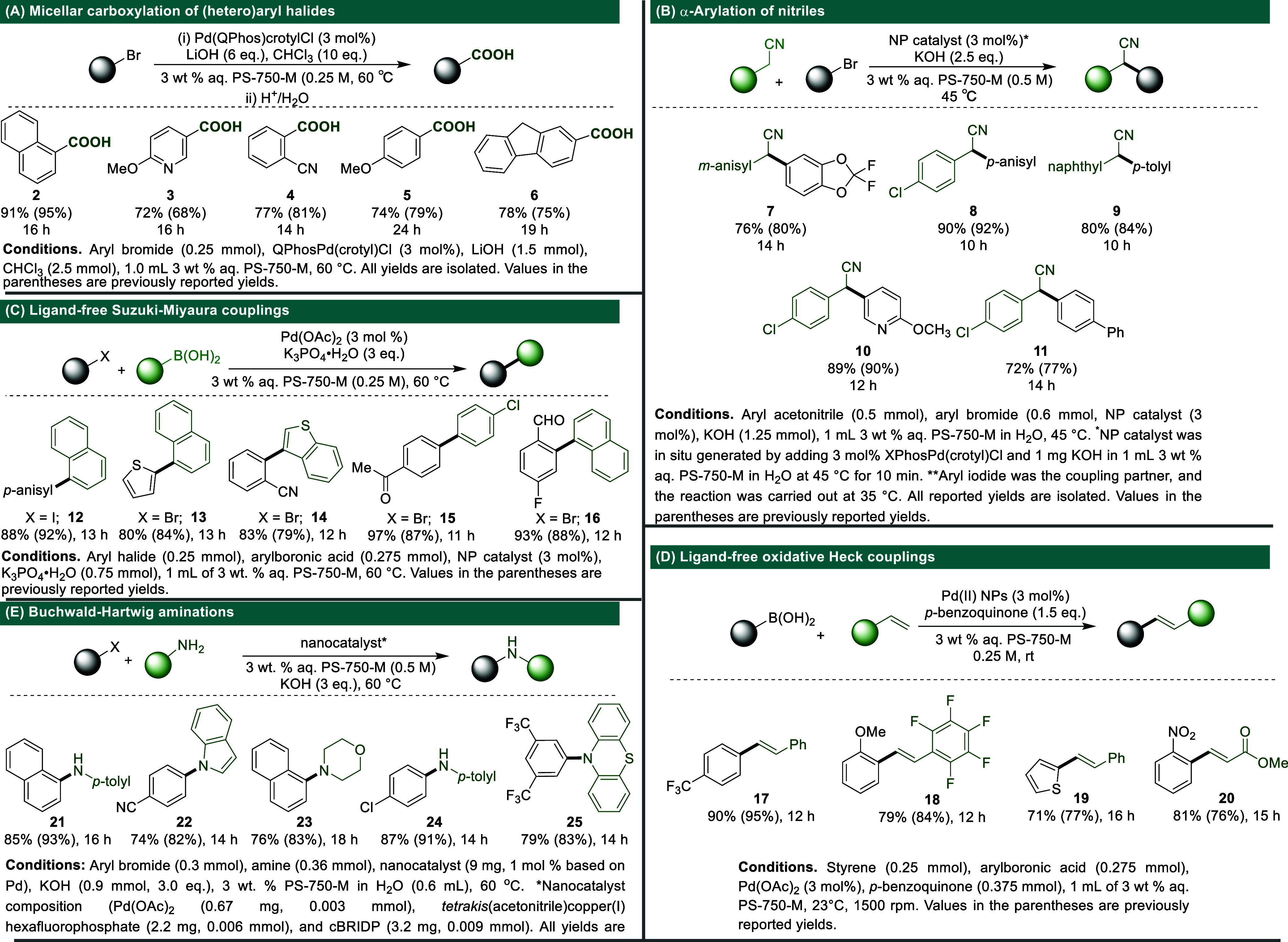
Activity Test of PS-750-M for Palladium-Catalyzed Cross-Couplings: (A) Carboxylation of (Hetero)­aryl halides, (B) *α*-Arylation of Nitriles, (C) Ligand-free Suzuki–Miyaura Couplings (D) Ligand-free Oxidative Heck Couplings, and (E) Buchwald–Hartwig Aminations

PS-750-M stabilizes water-sensitive intermediates such as carbanions and keteniminates, preventing quenching by water. Utilizing such intermediates and ultrasmall palladium nanoparticles generated in situ from XPhosPd­(crotyl)­Cl in 3 wt % aqueous PS-750-M, α-arylation of nitriles was achieved.^35^ The efficacy of new PS-750-M was demonstrated for this transformation. Examples include electron-withdrawing fluoro (**7**), chloro-substituted substrates (**8**, **11**), naphthyl (**9**), and pyridyl-containing bromides (**10**), all of which yield comparable results to those previously reported (Scheme 2B).

Micellar conditions proved crucial for achieving high yields and minimizing side reactions in ligand-free Suzuki couplings, as reactions performed poorly in conventional organic solvents.^23^ The performance of new PS-750-M was also validated using representative Suzuki coupling examples (**12**–**16**, Scheme 2C), demonstrating comparable catalytic activity. Substrates bearing cyano, acetyl, and thiophene groups exhibited consistent reactivity, confirming that the modified synthesis does not compromise the efficacy of PS-750-M.

Ligand-free Pd nanoparticles tend to aggregate in water, leading to a loss of catalytic activity unless stabilized by strong π-donor alkynes or ammonium salts.^37,38^ This issue is even more pronounced for Pd­(II) nanoparticles, which also suffer from precursor polymerization. Our amphiphile, PS-750-M, enriched with tertiary amide groups, creates a polar interior that dissolves Pd­(II) aggregates and generates ultrasmall Pd­(II) nanoparticles in situ. These nanoparticles are stabilized within the micellar core, likely through Pd–X–Pd linkages mediated by hydroxide or acetate anions.^20^ The newly synthesized PS-750-M was evaluated for this purpose, and the resulting nanoparticles were tested in ligand-free oxidative Heck couplings between arylboronic acids and styrenes (Scheme 2D). Representative substrates through a random selection process were examined, including those bearing electron-withdrawing trifluoromethyl (**17**), electron-donating methoxy (**18**), thiophene (**19**), and nitro groups (**20**). All substrates afforded the desired products in yields comparable to previously reported values.

The new PS-750-M was also evaluated for its catalytic performance in Buchwald–Hartwig amination reactions using bimetallic Cu–Pd nanoparticles.^36^ The results demonstrated that the activity of PS-750-M was comparable to that observed in our initial findings, indicating that the new synthetic route of PS-750-M did not compromise its efficiency. Notably, these tests were performed by a less-trained first year graduate student. This consistency underscores the robustness of the material and its suitability for cross-coupling applications under similar conditions. The representative examples **21**–**25** are shown in Scheme 2E.

### C–C and C–N Bond-Forming Reaction at the Carbonyl Center

The formation of C–C and C–N bonds at the carbonyl center is significant for constructing complex molecules from simple starting materials. These reactions facilitate the synthesis of important ketones and amides, contributing to molecular diversity. Therefore, the new PS-750-M was also evaluated for its activity in these critical bond-forming reactions:

### Biaryl Ketone Formation from a Carboxylic Acid Derivative

We previously demonstrated that ligand-free Pd nanoparticles in PS-750-M enable the synthesis of biaryl ketones from carboxylic acid derivatives.^24^ Ligand-free Pd(0) nanoparticles form efficiently in aqueous PS-750-M micelles from Pd_2_dba_3_, with the amide functionality aiding their formation and stabilization. This metal–micelle interaction imparts high catalytic efficiency in cross-coupling reactions between boronic acids and water-sensitive triazine acid adducts, affording nonsymmetrical biaryl ketones. In this work, PS-750-M prepared via a more sustainable route was evaluated for the same transformation. Coupling partners, including aromatic–aromatic (**26**, **28**), and aromatic–heteroaromatic (**27**, **29**), gave yields comparable to the original method (Scheme 3A). These results confirm that the greener PS-750-M retains catalytic performance.

**Scheme 3:**
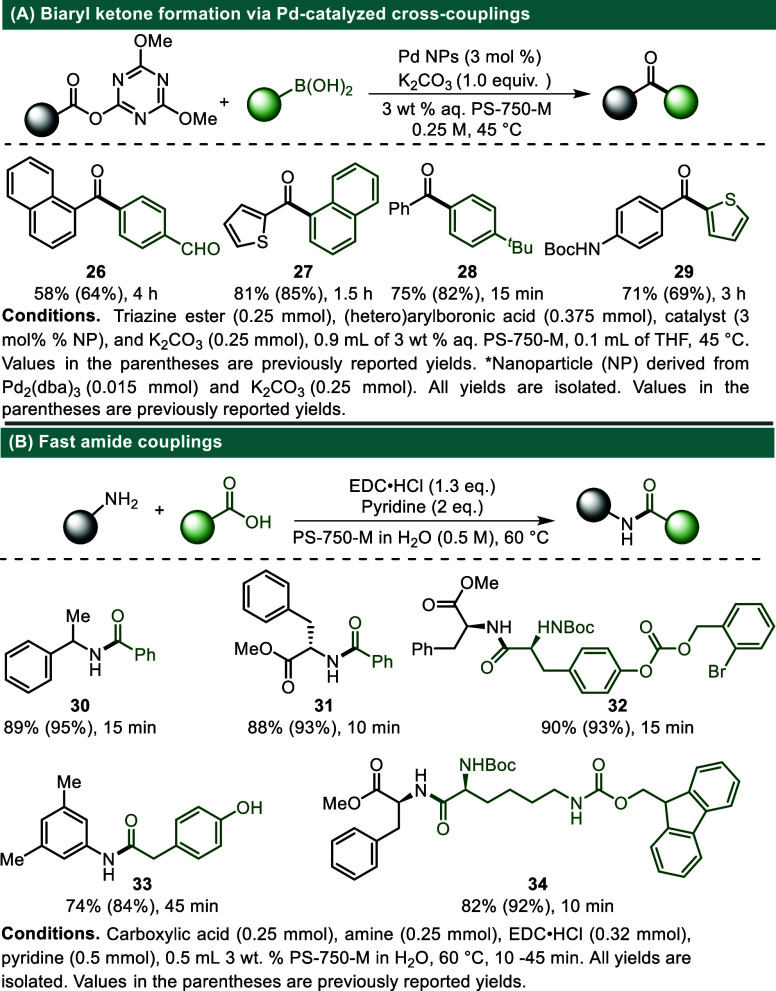
Reactions at the Carbonyl Center: (A) Biaryl Ketone Synthesis, (B) Fast Amide Couplings

### Fast Amide Couplings

PS-750-M forms micelles that mimic polar aprotic solvents (e.g., DMF, NMP) due to embedded tertiary amide functionalities, enabling highly efficient EDC-mediated couplings without hazardous additives typically used to suppress epimerization.^21^ A key innovation is the complete elimination of organic solvents during both reaction and isolation; products precipitate and are simply filtered, making the process highly sustainable. The newly synthesized PS-750-M exhibited comparable reactivity, as demonstrated in examples **30**–**34** (Scheme 3B). Functional groups, such as Boc, Fmoc, and hydroxy, remained intact during the desired reactions.

## Experimental Section

### Synthesis of **1b**

To a 500 mL one-neck round-bottom flask containing a PTFE-coated stir bar, l-proline **1a** (10 g, 0.086 mol) was dissolved in 20 mL of distilled water. The flask was cooled to 0 °C, and NaOH (6.94 g, 0.174 mol) dissolved in 20 mL of distilled water was added to the flask containing **1a** using a glass pipet. The mixture was stirred for 5 min at 0 °C. After stirring the mixture for 5 min, lauroyl chloride (21.06 mL, 0.086 mol) was added dropwise to the reaction mixture over 30 min using an addition funnel. After the complete addition of lauroyl chloride to the reaction mixture, the ice bath was left in place underneath the reaction flask, and the mixture was allowed to warm to room temperature (rt) for 2 h. After the reaction was complete, as monitored by TLC, the flask was recooled to 0 °C in an ice bath, and then the mixture was acidified with 1 N aqueous HCl (87 mL, 0.086 mol). With the addition of aq HCl, a white solid, appeared in the mixture (see Figure S1). The resulting solid was extracted with EtOAc (3 × 50 mL; see p S4 in the Supporting Information), and the combined organic extracts containing the desired compound were dried over anhydrous Na_2_SO_4_. Volatiles were removed under reduced pressure to obtain **1b** (24.5 g, 95%) as a waxy solid. EtOAc was recovered and reused for the preparation of the second batch of **1b**.

### Synthesis of PS-750-M (**1b**)

To a one-neck 500 mL round-bottom flask, **1b** (24.5 g, 0.0825 mol), Amberlyst 15­(H) (4.84 g), and mPEG-750 M (62.4 g, 0.0825 mol) were added. Then, toluene (160 mL, 0.5 M) was added, and the resulting mixture was stirred at 600 rpm while refluxing at 115 °C with a Dean–Stark apparatus for 48 h (for details and notes, please see section 2.1 in Supporting Information, pp S4 and S5). After complete consumption of the starting material as monitored by TLC, the mixture was allowed to cool to room temperature. The Amberlyst beads were removed by filtration through a sintered funnel using a Whatman filter paper. The solvent was removed under reduced pressure to yield a white, waxy solid of pure PS-750-M (82.1 g, 98%, Scheme S3). The recovered solvent was used to prepare the second batch of PS-750-M.

## Conclusions

The redesigned synthesis of PS-750-M represents a major advancement toward sustainable process chemistry. By reducing synthetic steps, eliminating chromatography, and replacing hazardous reagents with greener alternatives, the new route achieves exceptional improvements in the E-factor and PMI while maintaining high yields and reproducibility. The new method requires only 48 h for synthesis, whereas the old method takes about a week and uses significantly more energy. The catalytic performance of PS-750-M remains uncompromised, validating its suitability for complex aqueous-phase transformations. This work demonstrates that sustainability and scalability can coexist without sacrificing efficiency, offering a practical blueprint for integrating micellar catalysis into industrial workflows. Future efforts will focus on continuous-flow implementation and broader application of this strategy to other amphiphiles, further reinforcing the role of green chemistry in next-generation manufacturing.

## Supplementary Material



## Notes

General experimental details, experiment photographs, analytical data, scanned NMRs, and details of the material used (PDF)
