# Supplementary material for: Sustainable and Scalable Redesign of PS-750‑M Synthesis While Retaining Micellar Catalytic Efficiency
Source: ACS Sustain Chem Eng. 2026 Feb 23;14(9):4702–8. doi: 10.1021/acssuschemeng.5c13808 (PMC12959926; doi:10.1021/acssuschemeng.5c13808)
Supplement: Supplementary file 1 [file sc5c13808_si_001.pdf]

Sustainable and Scalable Redesign of PS-750-M Synthesis While Retaining Micellar Catalytic Efficiency

Ramesh Hiralal Choudhary,† Amna Akram,† Reda Zainab,† Ashik Chhetri,† Pritam Dolui,† Fabrice Gallou,‡ Michael Harmata,† Sachin Handa†*

†Department of Chemistry, 601 S College Ave, University of Missouri, Columbia, MO 65211, United States

‡Chemical & Analytical Development, Novartis Pharma AG, 4056 Basel, Switzerland

*Email: sachinhanda@missouri.edu

Number of Pages: 57
Number of Figures: 02
Number of Tables: 11
Number of Schemes: 04

Supporting Information

Contents **Page #**

1. General experimental details S2
2. Synthesis of surfactant PS-750-M S3-S5
3. Reproducibility test for previously reported reactions using PS-750-M synthesized via the new route S6-S15
4. Environmental (E)-factor calculations S16-S19
5. Process mass intensity (PMI) calculations S20-S21
6. Other green chemistry metrics S22-S24
7. References S25-S26
8. Analytical data S27-S37
9. NMR spectra S38-S57

1. GENERAL EXPERIMENTAL DETAILS

All manipulations were carried out under air unless noted. Commercially purchased chemicals were used without further purification unless otherwise mentioned. Solvent molarity listed in reaction schemes is relative to the limiting reagent. TLC plates (UV 254 indicator, aluminum-backed, 175-225 μm thickness) were purchased from Merck, and silica gel for column chromatography (standard grade, 230 – 400 mesh) was purchased from Silicycle. Celite was purchased from Merck. *L*-Proline was purchased from Ambeed, Dodecyl chloride was purchased from TCI, and Amberlyst-15(H) ion-exchange resin beads were purchased from Alfa-Aesar. Amberlyst beads were washed with methanol and dried under high vacuum prior to use. Toluene, ethyl acetate, and hexane were purchased from Fisher Scientific. Pure deuterated NMR solvents were purchased from MilliporeSigma. Aqueous solution of surfactant (PS-750-M) was prepared with distilled grade water and thoroughly purged with argon before use. Unless otherwise mentioned, all NMR spectra were recorded at 23 °C on a Bruker AVANCE III HD spectrometer (300, 400, and 500 MHz) with a commercial 5 mm probe. Reported chemical shifts are referenced to residual solvent peaks.[1]

2. SYNTHESIS OF SURFACTANT PS-750-M

2.1 A new synthetic plan for PS-750-M

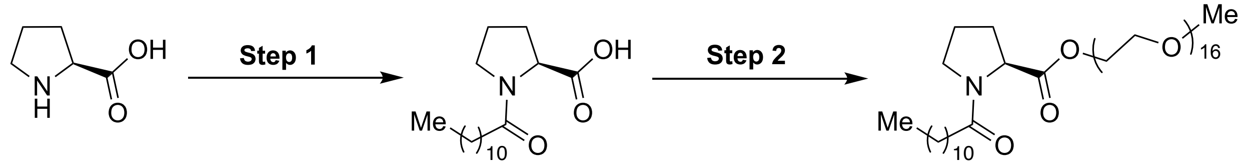

1a *L*-Proline **1b** **1** PS-750-M

Conditions:
Step 1. Lauroyl chloride (1 eq.), NaOH (2 eq.), H_2O, 0 °C- rt, 2 h.
Step 2. Amberlyst-15(H) (200 mg/g), mPEG-750 (1 eq.), toluene (0.5 M), 115 °C, 48 h.

Scheme S1. New synthetic plan for PS-750-M.

2.1a. Step 1. Synthesis of *N*-acylated proline

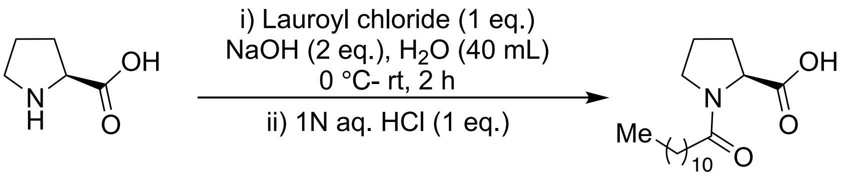

1a 10 g **1b** 24.5 g, 95%

Scheme S2. Synthesis of *N*-acylated proline.

Procedure. To a 500 mL one-neck round-bottom flask containing a PTFE-coated stir bar, *L*-proline **1a** (10 g, 0.086 mol) was dissolved in 20 mL of distilled water. The flask was cooled to 0 °C, and NaOH (6.94 g, 0.174 mol) dissolved in 20 mL of distilled water was added to the flask using a glass pipette. The mixture was stirred for 5 minutes at 0 ºC. After stirring the mixture for 5 mins, lauroyl chloride (21.06 mL, 0.086 mol) was added dropwise to the reaction mixture over 30 minutes using an addition funnel. After the complete addition of lauroyl chloride to the reaction mixture, the ice bath was left in place underneath the reaction flask, and the mixture was allowed to warm to room temperature (rt) for 2 hours. After the reaction was completed, as monitored by TLC, the flask was re-cooled to 0 °C using an ice bath, and then the mixture was acidified with 1N aqueous solution of HCl (87 mL, 0.086 mol). With the addition of aq. HCl, a white solid, appeared in the mixture (Figure S1). The resulting solid was extracted with EtOAc (3 × 50 mL), and the combined organic solvent containing the desired compound was dried over anhydrous

Na_2SO_4. Volatiles were removed under reduced pressure to obtain **1b** (24.5 g, 95%) as a waxy solid (Scheme S2). EtOAc was recovered and reused for the preparation of the second batch of **1b**.

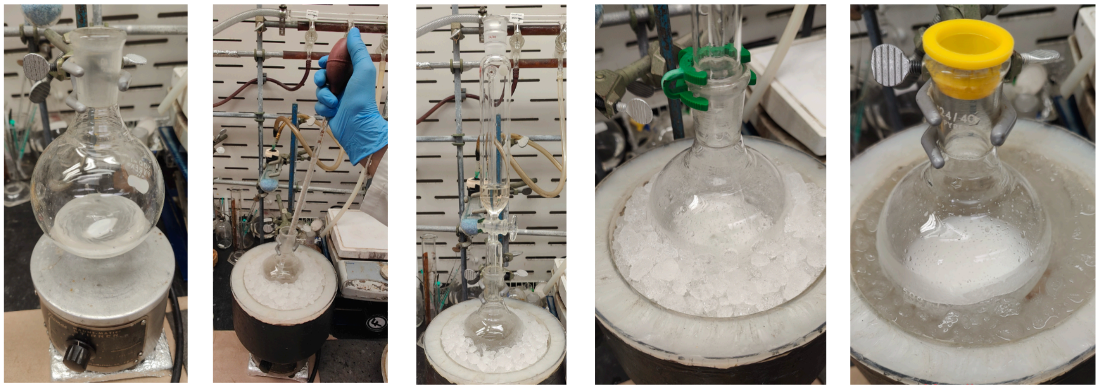

Proline + H_2O	Addition of NaOH	Addition of lauroyl chloride	Reaction mixture after completion of the reaction	After acidification

Figure S1. Progress of reaction in step 1.

2.1b. Step 2. Synthesis of PS-750-M

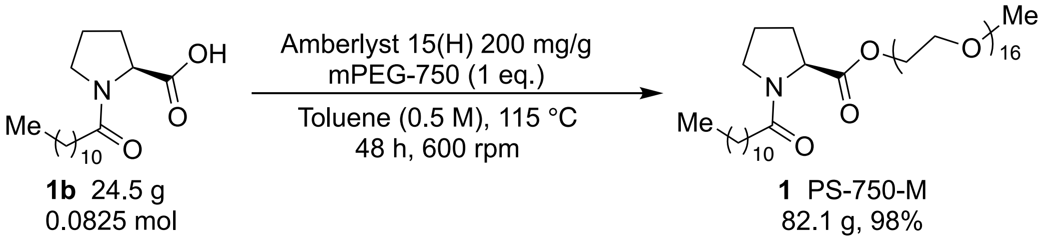

Scheme S3. Synthesis of PS-750-M surfactant.

Procedure. To a one-neck 500 mL round-bottom flask, **1b** (24.5 g, 0.0825 mol), Amberlyst 15(H) (4.84 g), and mPEG-750M (62.4 g, 0.0825 mol) were added. Then, toluene (160 mL, 0.5 M) was added, and the resulting mixture was stirred at 600 rpm while refluxing at 115 °C with a Dean-Stark apparatus for 48 hours. After complete consumption of the starting material as monitored by TLC, the mixture was allowed to cool to room temperature. The Amberlyst beads were removed by filtration through a sintered funnel using a Whatman filter paper. The solvent was removed and recovered under reduced pressure to obtain a white, waxy solid of pure PS-750-M (82.1 g, 98%, Scheme S3). The recovered solvent was used to prepare the second batch of PS-750-M.

Note: If the Amberlyst beads are broken due to excessive or improper stirring, a yellow coloration will appear in the reaction mixture. In that case, decolorization is necessary after the Amberlyst

beads are removed by filtration. Discoloration can be performed by adding activated charcoal to the reaction mixture and stirring the resulting mixture at 60 °C overnight. Then the mixture can be filtered through Celite to obtain a colorless solution of toluene containing pure PS-750-M. Thus, to avoid coloration, the stirring speed in step 2 should not exceed 600 rpm to prevent the disintegration of Amberlyst beads and to minimize the leaching of PTSA impurity into the product. Please see the images below for a reference.

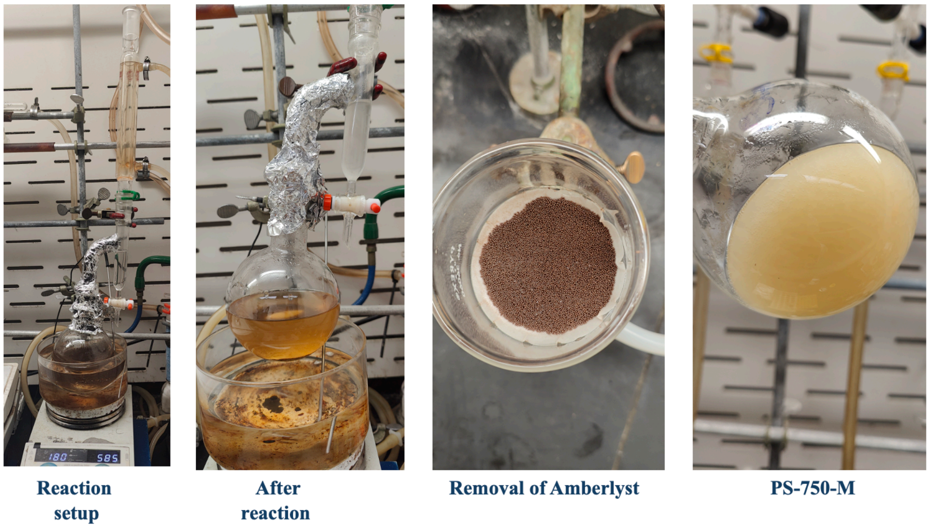

Figure S2. Reaction progress for step 2 at a higher stirring rate.

3. REPRODUCIBILITY TEST FOR PREVIOUSLY REPORTED REACTIONS USING PS-750-M SYNTHESIZED BY THE NEW ROUTE

3.1. Carboxylation of (hetero)aryl bromides via trichloromethyl carbanion in aqueous micelles

Table S1. Carboxylation of aryl bromides[a]

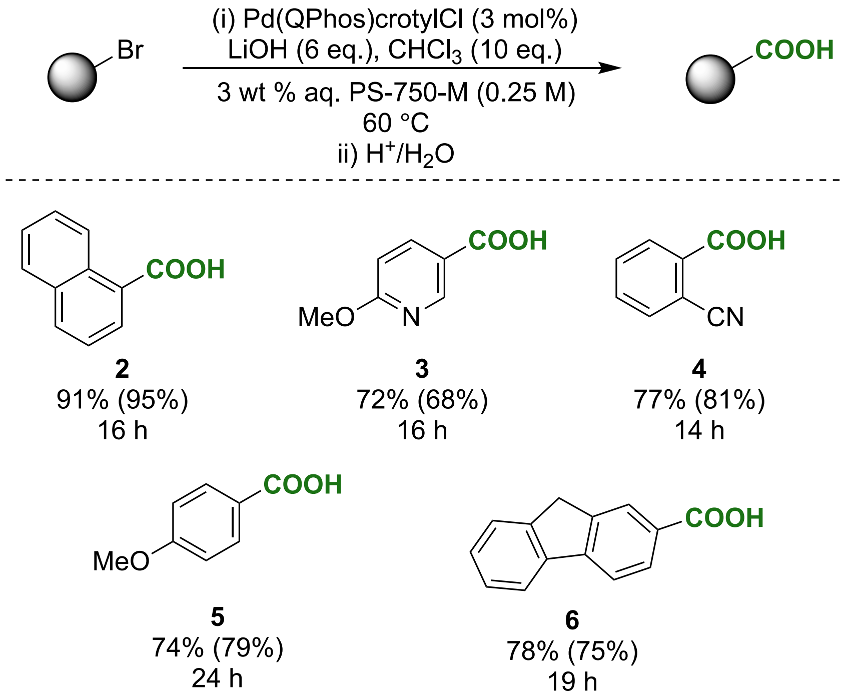

[a]**Conditions:** Aryl bromide (0.25 mmol), QPhosPd(crotyl)Cl (3 mol%), LiOH (1.5 mmol), $CHCl_3$ (2.5 mmol), 1.0 mL 3 wt % aq. PS-750-M, 60 °C. All yields are isolated. Values in the parentheses are previously reported yields.[2]

Procedure. To a clean and oven-dried 4.0 mL microwave vial containing a PTFE-coated magnetic stir bar, QPhosPd(crotyl)Cl (6.8 mg, 0.0075 mmol), LiOH (36 mg, 1.5 mmol), and aryl bromide (0.25 mmol; if solid, add after the addition of $CHCl_3$) were sequentially added. The reaction vial was closed with a rubber septum. The reaction mixture was evacuated and backfilled with nitrogen. This cycle was repeated three additional times. Then, 1.0 mL freshly degassed 3 wt. % aq. PS-750-M and $CHCl_3$ (200 μL, 2.5 mmol) were sequentially added. The resulting mixture was stirred at 60 °C for the time mentioned in Table S1. After complete consumption of the starting material as monitored by TLC or GC-MS, the mixture was allowed to cool to room temperature. An aqueous reaction mixture was extracted three times with 1 mL of EtOAc to remove any unreacted starting material or impurities. Afterward, the pH of the aqueous layer was adjusted to 1-2 by adding 1N

HCl, and the mixture was stirred at rt for 1- 2 minutes. The precipitate-containing aqueous layer was extracted with EtOAc (2 mL × 3). The combined organic layers were dried over anhydrous sodium sulfate (Na_2SO_4). The combined organic layers were passed through a silica plug. Volatiles were removed under reduced pressure to obtain the desired product.

3.2. Ultrasmall Pd nanoparticles mediated *α*-arylation of nitriles

Table S2. *α*-arylation of nitriles[a]

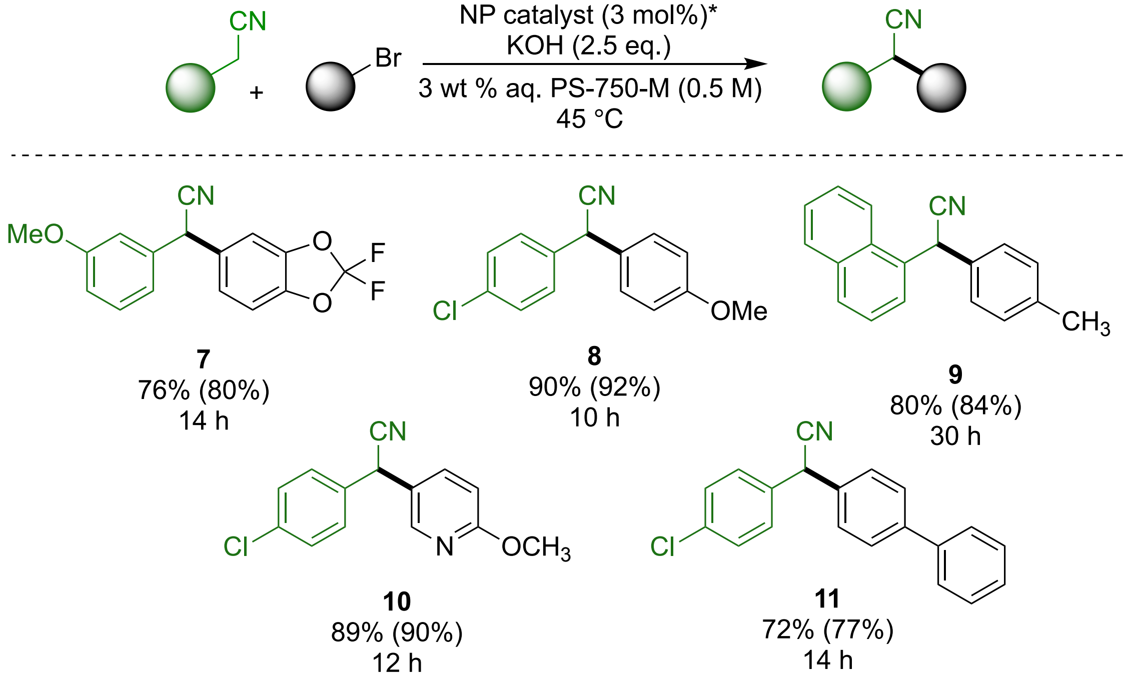

[a]**Conditions:** Aryl acetonitrile (0.5 mmol), aryl bromide (0.6 mmol, NP catalyst (3 mol%), KOH (1.25 mmol), 1 mL 3 wt. % aq. PS-750-M in H_2O, 45 °C. *NP catalyst was in situ generated by adding 3 mol% XPhosPd(crotyl)Cl and 1 mg KOH in 1 mL 3 wt % aq. PS-750-M in H_2O at 45 °C for 10 min. All reported yields are isolated. Values in the parentheses are previously reported yields.[3]

Procedure. In a 4.0 mL flame-dried microwave vial containing a PTFE-coated magnetic stir bar, XPhosPd(crotyl)Cl (10 mg, 0.015 mmol), aryl acetonitrile (0.5 mmol), aryl bromide (0.6 mmol), and KOH (70 mg, 1.25 mmol) were sequentially added under a nitrogen atmosphere. 1 mL aqueous solution of 3 wt % aq. PS-750-M was added to the reaction mixture. The septum was closed and wrapped with PTFE tape and black electrical tape. The mixture was evacuated and backfilled with nitrogen. The reaction mixture was stirred under a nitrogen atmosphere at 45 °C for the time

mentioned in Table S2. After complete consumption of the starting material as monitored by TLC, the reaction mixture was allowed to cool to rt. EtOAc (1.0 mL) was added to the reaction mixture, and the mixture was stirred for a minute at rt. Stirring was stopped, and the organic layer was allowed to separate. The organic layer was withdrawn, and this extraction cycle was repeated for an additional cycle. Combined organic layers were dried over anhydrous sodium sulfate. Volatiles were removed under reduced pressure, and the crude product was purified by flash chromatography over silica gel using a 1:1 EtOAc/hexanes eluent.

3.3. Ligand-free Pd(0) catalyzed Suzuki-Miyaura cross-couplings in water

Table S3. Suzuki-Miyaura couplings[a]

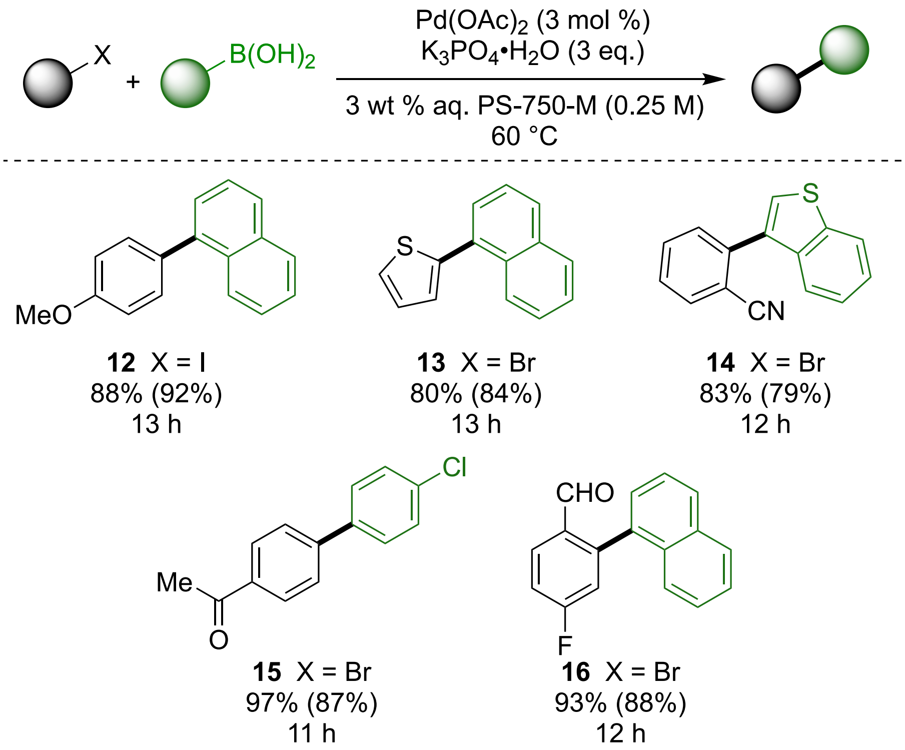

[a]**Conditions**: Aryl halide (0.25 mmol), arylboronic acid (0.275 mmol), NP catalyst (3 mol%), K_3PO_4•H_2O (0.75 mmol), 1 mL of 3 wt. % aq. PS-750-M, 60 °C. Values in the parentheses are previously reported yields.[4]

Procedure. In an oven-dried 4 mL reaction vial equipped with a PTFE-coated magnetic stir bar, palladium(II)acetate (1.7 mg, 0.0075 mmol) as a nanoparticle precursor, K_3PO_4•H_2O (172.5 mg, 0.75 mmol), and appropriate arylboronic acid (0.025 mmol) were charged. The vial was closed with a rubber septum properly and wrapped with PTFE tape and parafilm. The reaction vial was

evacuated and backfilled with nitrogen three times. Under positive nitrogen pressure, freshly nitrogen-purged 1 mL 3 wt. % aq. PS-750-M was added. The mixture was evacuated and backfilled with nitrogen three times. The vial was heated at 60 °C on a pre-heated stir plate and stirred for 30 minutes. The color of the reaction mixture darkens. The vial was then removed from the hot plate and allowed to cool to room temperature. Then, arylboronic acid (0.25 mmol) and aryl halide (0.25 mmol) were added under a positive nitrogen atmosphere. The vial was closed with a rubber septum and evacuated and backfilled with nitrogen. This cycle was repeated twice more. Then, the mixture was stirred and transferred to a pre-heated stir plate at 60 °C for a specified time mentioned in Table S3. After complete consumption of aryl halide as monitored by TLC, the reaction vial was cooled to rt. The septum was removed, and 1-2 mL EtOAc was added to the reaction mixture to extract the product. The organic layer was separated using a pipette. This extraction was repeated an additional two times. The combined organic layers were dried over Na_2SO_4. Volatiles were evaporated under reduced pressure to obtain the crude product, which was then purified by column chromatography over silica gel using hexanes/ethyl acetate as eluent.

3.4. Ligand-free Oxidative Heck-type coupling

Table S4. Mizoroki-Heck-type couplings[a]

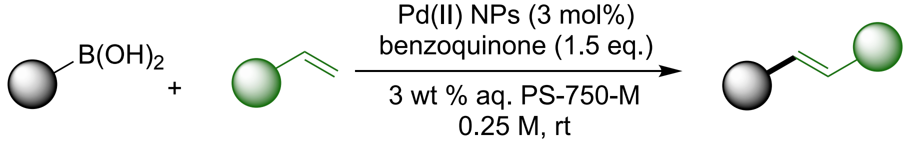

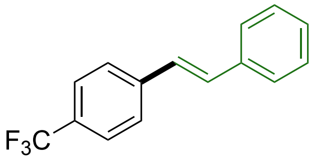

17
90% (95%)
12 h

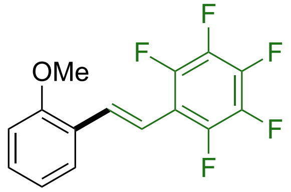

18
79% (84%)
12 h

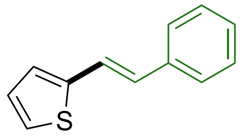

19
71% (77%)
16 h

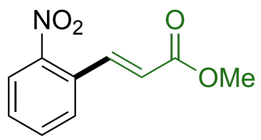

20
81% (76%)
15 h

[a]**Conditions:** Styrene (0.25 mmol), arylboronic acid (0.275 mmol), $Pd(OAc)_2$ (3 mol%), *p*-benzoquinone (0.375 mmol), 1 mL of 3 wt % aq PS-750-M, 23°C, 1500 rpm. Values in the parentheses are previously reported yields.[5]

Procedure. A 4.0 mL reaction vial containing a PTFE-coated magnetic stir bar was charged with nanoparticle precursor palladium(II)acetate (1.7 mg, 0.0075 mmol). Later, 1 mL 3 wt % aq. PS-750-M was added, and the vial was closed with a screw cap. Then, the reaction vial was transferred to a pre-heated stir plate at 45 °C and stirred for 50 minutes. After cooling to room temperature, the reaction vial was opened, and arylboronic acid (0.275 mmol), *p*-benzoquinone (108 mg, 0.375 mmol), and styrene (0.25 mmol) were added. The reaction vial was closed with a screwcap and then stirred (1500 rpm) at room temperature. After complete consumption of styrene as monitored by TLC or GC-MS, the reaction vial was opened, and 1-2 mL ethyl acetate was added. The mixture was then gently stirred for 2 minutes at rt. Stirring was stopped, and the organic layer was separated using a pipette. This extraction was repeated one more time. The combined organic layers were passed through a celite-pad filled in the pipette. Volatiles were evaporated under reduced pressure

to obtain the crude product, which was then purified by flash chromatography over silica gel using hexanes/ethyl acetate as eluent.

3.5. Bimetallic nanocatalysis for Buchwald-Hartwig amination

Table S5. Buchwald-Hartwig aminations[a]

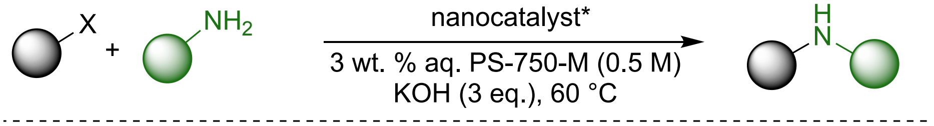

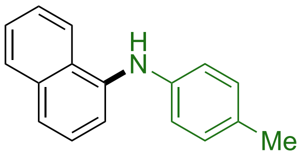

21
85% (93%)
16 h

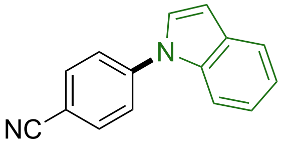

22
74% (82%)
14 h

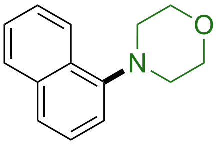

23
76% (83%)
18 h

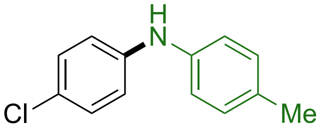

24
87% (91%)
14 h

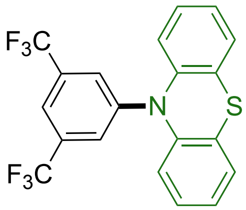

25
79% (83%)
14 h

[a]**Conditions:** Aryl bromide (0.3 mmol), aryl amine (0.36 mmol), nanocatalyst (9 mg, 1 mol % based on Pd), KOH (0.9 mmol, 3.0 eq.), 3 wt. % PS-750-M in H_2O (0.6 mL), 60 °C. Nanoparticle composition: $(Pd(OAc)_2$ (0.67 mg, 0.003 mmol), *tetrakis*(acetonitrile)copper(I) hexafluorophosphate (2.2 mg, 0.006 mmol), and cBRIDP (3.2 mg, 0.009 mmol). All yields are isolated. Values in the parentheses are previously reported yields.[6]

Procedure. A 4.0 mL flame-dried vial was equipped with a Teflon-coated magnetic stir bar and was charged palladium(II)acetate (0.67 mg, 0.003 mmol), followed by the addition of *tetrakis*(acetonitrile)copper(I) hexafluorophosphate (2.2 mg, 0.006 mmol), and ligand cBRIDP (3.2 mg, 0.009 mmol). The reaction vial was closed with a rubber septum and was evacuated and backfilled with nitrogen. This cycle was repeated twice more. 0.2 mL dry THF was added to the reaction vial. After covering the septum with parafilm, the mixture was gently heated with the heat gun to get a dark red color. Afterward, the mixture was stirred at 60 °C under a nitrogen atmosphere for 15 minutes. Then, the reaction mixture was allowed to cool to rt. Under positive nitrogen

pressure, the septum was opened, and activated charcoal powder (3.6 mg, 0.3 mmol) was added to the reaction mixture. The vial was again closed with a rubber septum and covered with parafilm after one vacuum-nitrogen cycle. The mixture was stirred again at 60 °C for 15 minutes to generate an active nanocatalyst. THF was evaporated from the reaction mixture at rt. (Hetero)aryl bromide (0.3 mmol), aryl(alkyl) amine (0.36 mmol), and KOH (50.4 mg, 0.9 mmol) were added to the reaction mixture under positive nitrogen pressure. The vial was closed with a rubber septum and covered with electric black tape after the three rounds of the vacuum nitrogen cycle. Freshly degassed 0.6 mL aqueous solution of 3 wt. % aq. PS-750-M was added to the reaction mixture. Further, the reaction mixture was purged with nitrogen for 2 min, and then it was stirred at 60 °C. After the complete consumption of the starting materials, as monitored by TLC and GC-MS, the reaction vial was removed from the oil bath and cooled to room temperature, and 1 mL of ethyl acetate was added to the reaction mixture. The mixture was then gently stirred for 1–2 min at rt. Stirring was stopped, and the organic layer was allowed to separate from the aqueous layer. The organic layer was removed from the aqueous layer using a pipette. The leftover aqueous layer was extracted one or two more times. The combined organic layers were dried over anhydrous Na_2SO_4. Volatiles were removed under reduced pressure to obtain a crude product, which was then purified by column chromatography on silica gel using a mixture of EtOAc/hexanes as the eluent.

3.6. Biaryl ketone formation from carboxylic acid derivatives

Table S6. Biaryl ketone formation[a]

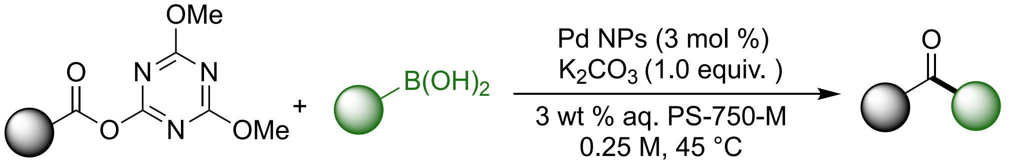

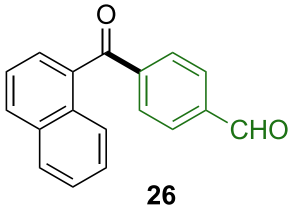

26
58% (64%)
4 h

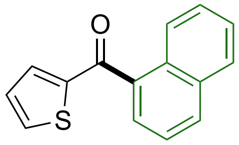

27
81% (85%)
1.5 h

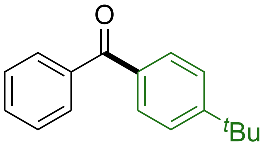

28
75% (82%)
15 min

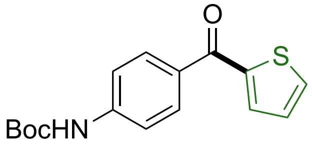

29
71% (69%)
3 h

[a]**Conditions:** Triazine ester (0.25 mmol), (hetero)arylboronic acid (0.375 mmol), catalyst (3 mol % **NP**), and K_2CO_3 (0.25 mmol), 0.9 mL of 3 wt % aq. PS-750-M, 0.1 mL of THF, 45 °C. Values in the parentheses are previously reported yields. Nanoparticles (**NPs**) were derived from $Pd_2(dba)_3$ (0.015 mmol) and K_2CO_3 (0.25 mmol). Values in the parentheses are previously reported yields.[7]

Procedure. In an oven-dried 4 mL reaction vial equipped with a PTFE-coated magnetic stir bar, triazine ester (0.25 mmol), Pd_2dba_3 (3.43 mg, 1.5 mol%, equivalent to 3 mol% Pd NPs), K_2CO_3 (0.25 mmol), and arylboronic acid (0.75 mmol) were added. The vial was sealed with a rubber septum. The mixture was evacuated and backfilled with nitrogen three times. Freshly nitrogen purged 0.9 mL of 3 wt % aq. PS-750-M and 0.1 mL of THF were added to the reaction mixture. The septum was tightly covered with tape. The vial was then transferred to a preheated stir plate at 45 °C, and the mixture was stirred under an inert atmosphere. The reactions were stirred for the time specified in Table S7. Then, the reaction mixture was cooled to rt. The reaction vial was opened, and 2 mL of ethyl acetate was added. The mixture was then gently stirred for 2 minutes

at rt. The organic layer was separated using a pipette. This extraction procedure was repeated for additional two times. The combined organic layers were dried over anhydrous Na_2SO_4. Volatiles were removed under reduced pressure to obtain the crude product, which was then purified by column chromatography over silica gel using hexanes/ethyl acetate as an eluent.

3.7. Column chromatography-free fast-amide coupling in aqueous micelles of PS-750-M

Table S7. Fast amide couplings[a]

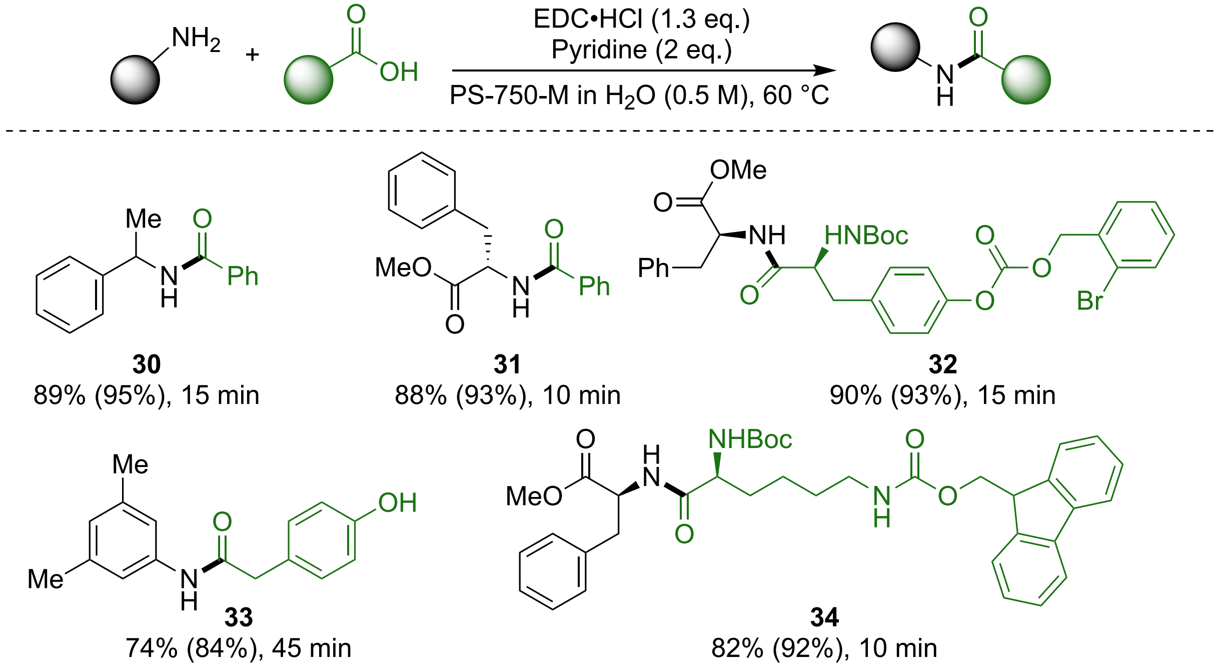

[a]**Conditions:** Carboxylic acid (0.25 mmol), amine (0.25 mmol), EDC•HCl (0.32 mmol), pyridine (0.5 mmol), 0.5 mL 3 wt. % aq. PS-750-M in H_2O, 60 °C, 10 - 45 min. All yields are isolated. Values in the parentheses are previously reported yields.[8]

Procedure. In an oven-dried 4 mL reaction vial equipped with a PTFE-coated magnetic stir bar, carboxylic acid (0.25 mmol, 1 equiv.), amine (0.25 mmol, 1 equiv.) and EDC•HCl (0.32 mmol, 1.3 equiv.) were weighed. The reaction vial was sealed with a rubber septum. 0.5 mL 3 wt. % aqueous PS-750-M solution was added to the reaction mixture, and the septum was covered with PTFE tape. After stirring the mixture for one minute at room temperature, pyridine (0.5 mmol, 2.0 equiv.) was added to the reaction mixture, and the mixture was allowed to stir at 60 °C on a pre-heated oil bath. The product was formed within a few minutes and solidified in a reaction vial. Reaction progress was monitored by TLC, and after complete consumption of the starting materials, the

reaction mixture was allowed to cool down to room temperature. The solid formed was filtered through Whatman filter paper, washed with deionized water (3 x 1mL), dried under high vacuum, and characterized by ^{1}H NMR.

4. E-FACTOR CALCULATIONS

4.1 E-Factor Calculation for synthesis of PS-750-M

The E-factor was calculated based on the formula reported in the literature.[9]

$$E - factor = \frac{\Sigma\, m(raw\ materials) + \Sigma\, m(reagents) + \Sigma\, m(reaction\ solvents) - m(desired\ product)}{m(desired\ product)}$$

Traditional route[10]

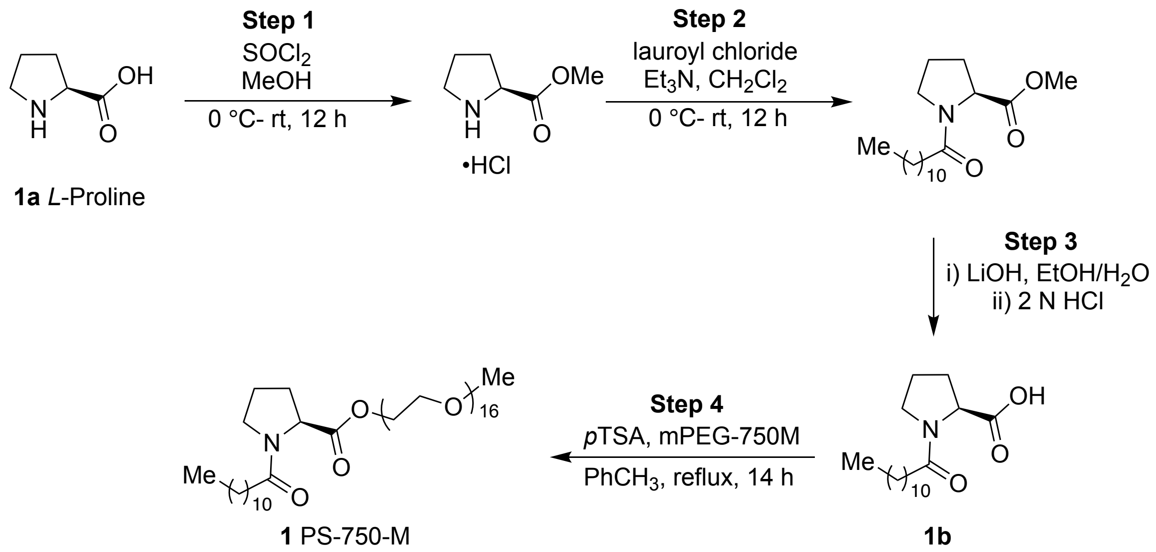

Scheme S4. Traditional route for the synthesis of PS-750-M.

Step 1:

L-Proline **1a** = 5 g

Thionyl chloride = 3.8 mL $\left(1.63\,\frac{\mathrm{g}}{\mathrm{mL}}\right) = 6.19\ \mathrm{g}$

Methanol = $60\ \mathrm{mL}\ \left(0.7918\,\frac{\mathrm{g}}{\mathrm{mL}}\right) = 47.46\ \mathrm{g}$

$$= \frac{(5\ \mathrm{g}\ +\ 6.19\ \mathrm{g} +\ 47.46\ \mathrm{g}) -\ (6.5\ \mathrm{g})}{(6.5\ \mathrm{g})}$$

$$= \frac{58.65\ \mathrm{g}\ -\ 6.5\ \mathrm{g}}{6.5\ \mathrm{g}}$$

= 52.15 g/ 6.5 g

= 8.023

Step 2:

Methyl *L*-prolinate hydrochloride = 6.5 g

Lauroyl chloride = $13.7\ \mathrm{mL}\left(0.946\,\frac{\mathrm{g}}{\mathrm{mL}}\right) = 12.96\ g$

Triethylamine = $15.2\ \mathrm{mL}\ \left(0.726\,\frac{\mathrm{g}}{\mathrm{mL}}\right) = 11.03\ g$

Dichloromethane = $100\ \mathrm{mL}\ \left(1.333\,\frac{\mathrm{g}}{\mathrm{mL}}\right) = 133.3$

Product obtained = 9.6 g

$$= \frac{(6.5\ \mathrm{g}\ +\ 12.96\ \mathrm{g}\ + 11.03\ \mathrm{g} +\ 133.3\ \mathrm{g}) - (9.6\ \mathrm{g})}{9.6\ \mathrm{g}}$$

$$= \frac{(163.73\ \mathrm{g}\ -\ 9.6\ \mathrm{g})}{9.6\ \mathrm{g}}$$

= 16.06

Step 3:

Methyl dodecanoyl-*L*-prolinate = 9.6 g

Lithium hydroxide monohydrate = 5.2 g

Ethanol = $25\ \mathrm{mL}\ \left(0.789\,\frac{\mathrm{g}}{\mathrm{mL}}\right)\ = 19.72\ g$

Water = $25\ \mathrm{mL}\left(0.997\,\frac{\mathrm{g}}{\mathrm{mL}}\right)\ = 24.92\ g$

$$= \frac{(9.6\ \mathrm{g}\ +\ 5.2\ \mathrm{g}\ + 19.72\ \mathrm{g}\ +\ 24.92\ \mathrm{g}) - (8.0\ \mathrm{g})}{8.0\ \mathrm{g}}$$

$$= \frac{(59.44\ \mathrm{g} -\ 8\ \mathrm{g})}{8\ \mathrm{g}}$$

= 6.43

Step 4:

dodecanoyl-*L*-proline = 8.0 g

p-Toluenesulfonic acid monohydrate = 0.040 g

mPEG-750 =40.5 g

Toluene = 100 mL $\left(0.8639\,\frac{\mathrm{g}}{\mathrm{mL}}\right) = 86.39\ g$

$$= \frac{(8.0\ \mathrm{g}\ +\ 0.040\ \mathrm{g}\ +\ 40.5\ \mathrm{g}\ +\ 86.39\ \mathrm{g}) - (23.5\ \mathrm{g})}{23.5\ \mathrm{g}}$$

$$= \frac{(134.93\ \mathrm{g}\ -\ 23.5\ \mathrm{g})}{23.5\ \mathrm{g}}$$

= 4.74

Net E-Factor (excluding solvents and silica gel used in purification processes) = 8.023 + 16.06 + 6.43 + 4.74 = 35.25

<u>New Route:</u>

Step 1. Synthesis of *N*-acylated proline (please refer to scheme S2 for details)

L-Proline **1a** = 10 g

Lauroyl chloride = 18.99 g

Sodium hydroxide = 6.94 g

Water used as solvent in reaction = 40 mL$\left(0.997\,\frac{\mathrm{g}}{\mathrm{mL}}\right)$ = 39.88

1b = 24.5 g

$$= \frac{(10\ \mathrm{g}\ +\ 18.99\ \mathrm{g} +\ 6.94\ \mathrm{g} +\ 39.88\ \mathrm{g}) -\ (24.5\ \mathrm{g})}{(24.5\ \mathrm{g})}$$

$$= \frac{(75.81\ \mathrm{g} - 24.5\ \mathrm{g})}{24.5\ \mathrm{g}}$$

= 2.09

Step 2. Synthesis of PS-750-M (please refer to scheme S3 for details)

1b = 24.5 g

mPEG-750 = 62.37 g

Amberlyst 15(H) = 4.9 g

Toluene was recovered

$$= \frac{(24.5\text{ g} + 62.37\text{ g} + 4.9\text{ g} - (82.1\text{ g})}{(82.1\text{ g})}$$

$$= \frac{91.77\text{ g} - 82.1\text{ g}}{82.1\text{ g}} = \frac{9.67\text{ g}}{82.1\text{ g}}$$

= 0.11

Net E-Factor = 2.09 + 0.11 = 2.2

Table S8. Side-to-Side comparison of E-factor calculations for traditional versus new route

	**Traditional route**	**New route**
Step 1	8.023	2.09
Step 2	16.06	0.11
Step 3	6.43	-
Step 4	4.74	-
Overall	35.25	2.2

5. PROCESS MASS INTENSITY (PMI) CALCULATIONS

5.1. PMI calculation of PS-750-M synthesis (traditional route)

The PMI calculations for traditional[10] and the new route of PS-750-M synthesis were calculated based on the formula reported in literature.[11]

Table S9. Amounts (g) of reactants, catalysts, reagents and solvents used during the traditional route of synthesis

Compounds	Amount (g)
L-proline	5.0
Thionyl chloride	6.194
Methanol	47.46
Lauryl chloride	12.96
Triethyl amine	11.0352
Dichloromethane	133.3
Lithium hydroxide monohydrate	5.2
Ethanol	19.725
Water	24.925
2N HCl	103
p-toluenesulfonic acid	0.04
mPEG-750	40.5
Toluene	86.69
Ethyl acetate	556.2
Hexanes	184.52
Silica gel	180

PMI = Total mass used in the synthesis of product / Mass of product
= 1416.75 / 23.5
= 60.29

5.2 PMI calculation of PS-750-M synthesis (new route)

Table S10. Amounts (g) of reactants, catalysts, reagents, and solvents used during the synthetic route

Ingredients	Amount (g)
L-proline	10
Sodium hydroxide	6.94
Lauryl chloride	18.99
Water	39.88
Ethyl acetate	135.3
1N HCl	88.74
Amberlyst 15(H)	4.9
mPEG-750	62.37
Toluene (recovered)	142.54

PMIr (Without inclusion of recovered solvent) = (Total mass used in the synthesis of the product) – (Mass of recovered solvent)/ Mass of product

= (509.66 – 142.54)/ 82.1 = 4.47

PMI (With inclusion of recovered solvent) = (Total mass used in the synthesis of product)/ Mass of product

= 509.66/ 82.1

= 6.2

Table S11. Side-to-Side comparison of PMI calculations for traditional versus new route

	Traditional route	New route
PMI	60.29	6.2

6. OTHER GREEN CHEMISTRY METRICS

6.1 Atom economy (AE) calculations

The AE was calculated based on the formula reported in the literature.[12]

$$AE = \frac{molecular\ weight\ of\ product}{total\ molecular\ weight\ of\ all\ reactants} \times 100$$

Traditional route

$$\mathrm{AE} = \frac{\text{mol. wt. of } \mathbf{1}}{\text{Mol. wt. of } (\mathbf{1a}) + \text{mol. wt. of SOCl2} + \text{mol. wt. of MeOH} + \text{mol. wt. of lauroyl chloride} + \text{mol. wt. of mPEG} - 750} \times 100$$

$$\mathrm{AE} = \frac{1016.31}{115.13 + 118.97 + 32.04 + 218.76 + 750} \times 100$$

$$\mathrm{AE} = \frac{1016.31}{1134.9} \times 100$$

$$\mathrm{AE}\ (\%) = 89.5$$

New Route

$$\mathrm{AE} = \frac{\text{mol. wt. of } \mathbf{1}}{\text{Mol. wt. of } (\mathbf{1a}) + \text{mol. wt. of lauroyl chloride} + \text{mol. wt. of mPEG} - 750} \times 100$$

$$\mathrm{AE} = \frac{1016.31}{115.13 + 218.76 + 756} \times 100$$

$$\mathrm{AE} = \frac{1016.31\ \mathrm{g/mol}}{1089.89\ \mathrm{g/mol}} \times 100$$

$$\mathrm{AE}\ (\%) = 93.2$$

6.2 Reaction mass efficiency (RME) calculations

$$RME = \frac{mass\ of\ isolated\ product}{total\ mass\ of\ reactants} \times 100$$

Traditional route:

Step 1:

L-Proline **1a** = 5 g

Thionyl chloride = 3.8 mL $\left(1.63\,\frac{\mathrm{g}}{\mathrm{mL}}\right) = 6.19\ \mathrm{g}$

Methanol = 60 mL $\left(0.7918\,\frac{\mathrm{g}}{\mathrm{mL}}\right) = 47.46\ \mathrm{g}$

Step 2:

Methyl *L*-prolinate hydrochloride = 6.5 g

Lauroyl chloride = 13.7 mL $\left(0.946\,\frac{\mathrm{g}}{\mathrm{mL}}\right) = 12.96\ g$

Step 3:

Methyl dodecanoyl-*L*-prolinate = 9.6 g

Step 4:

dodecanoyl-*L*-proline = 8.0 g

mPEG-750 = 40.5 g

Isolated product (**1**) = 23.5 g

$$\mathrm{RME} = \frac{\text{wt. of isolated product}}{\text{wt. of }(\mathbf{1a}) + \text{wt. of SOCl2} + \text{wt. of MeOH} + \text{wt of lauroyl chloride} + \ + \text{wt. of mPEG} - 750} \times 100$$

$$\mathrm{RME} = \frac{23.5\ \mathrm{g}}{5.0\ \mathrm{g}\ + 6.19\ \mathrm{g} + 47.46\ \mathrm{g} + 12.96\ \mathrm{g} + 40.5\ \mathrm{g}} \times 100$$

$$\mathrm{RME} = \frac{23.5\ \mathrm{g}}{112.11} \times 100$$

RME = 20.96 %

<u>**New Route:**</u>

Step 1:

L-Proline **1a** = 10 g

Lauroyl chloride = 18.99 g

Step 2

mPEG-750 = 62.37 g

Isolated product (**1**) = 82.1 g

$$RME = \frac{\text{wt. of isolated product}}{\text{wt. of proline} + \text{wt. of Lauroyl chloride} + \text{wt. of mPEG} - 750} \times 100$$

$$= \frac{82.1\ \text{g}}{10\ \text{g} + 18.99\ \text{g} + 62.37\ \text{g}} \times 100$$

$$= \frac{82.1\text{g}}{91.36\ \text{g}} \times 100$$

RME = 89.86 %

7. REFERENCES

(1) Fulmer, G. R.; Miller, A. J. M.; Sherden, N. H.; Gottlieb, H. E.; Nudelman, A.; Stoltz, B. M.; Bercaw, J. E.; Goldberg, K. I. NMR Chemical Shifts of Trace Impurities: Common Laboratory Solvents, Organics, and Gases in Deuterated Solvents Relevant to the Organometallic Chemist. *Organometallics* **2010**, *29*, 2176–2179. https://doi.org/10.1021/om100106e.

(2) Ansari, T. N.; Sharma, S.; Hazra, S.; Hicks, F.; Leahy, D. K.; Handa, S. Trichloromethyl Carbanion in Aqueous Micelles: Mechanistic Insights and Access to Carboxylic Acids from (Hetero)Aryl Halides. *ACS Catal.* **2022**, *12*, 15686–15695. https://doi.org/10.1021/acscatal.2c05288.

(3) Bihani, M.; Ansari, T. N.; Finck, L.; Bora, P. P.; Jasinski, J. B.; Pavuluri, B.; Leahy, D. K.; Handa, S. Scalable α-Arylation of Nitriles in Aqueous Micelles Using Ultrasmall Pd Nanoparticles: Surprising Formation of Carbanions in Water. *ACS Catal.* **2020**, *10*, 6816–6821. https://doi.org/10.1021/acscatal.0c01196.

(4) Ansari, T. N.; Sharma, S.; Hazra, S.; Jasinski, J. B.; Wilson, A. J.; Hicks, F.; Leahy, D. K.; Handa, S. Shielding Effect of Nanomicelles: Stable and Catalytically Active Oxidizable Pd(0) Nanoparticle Catalyst Compatible for Cross-Couplings of Water-Sensitive Acid Chlorides in Water. *JACS Au* **2021**, *1*, 1506–1513. https://doi.org/10.1021/jacsau.1c00236.

(5) Ansari, T. N.; Jasinski, J. B.; Leahy, D. K.; Handa, S. Metal–Micelle Cooperativity: Phosphine Ligand-Free Ultrasmall Palladium(II) Nanoparticles for Oxidative Mizoroki–Heck-Type Couplings in Water at Room Temperature. *JACS Au* **2021**, *1*, 308–315. https://doi.org/10.1021/jacsau.0c00087.

(6) Ansari, T. N.; Taussat, A.; Clark, A. H.; Nachtegaal, M.; Plummer, S.; Gallou, F.; Handa, S. Insights on Bimetallic Micellar Nanocatalysis for Buchwald–Hartwig Aminations. *ACS Catal.* **2019**, *9*, 10389–10397. https://doi.org/10.1021/acscatal.9b02622.

(7) Kaur, G.; Jasinski, J. B.; Gallou, F.; Handa, S. Metal-Micelle Interaction Leading to Spontaneous Formation of Ligand-Free Palladium(0) Nanoparticles: Highly Efficient Catalysis Enabling Biaryl

Ketone Formation from Carboxylic Acid Derivatives. *ACS Appl. Mater. Interfaces* **2022**, *14*, 50947-50955. https://doi.org/10.1021/acsami.2c15099.

(8) Sharma, S.; Buchbinder, N. W.; Braje, W. M.; Handa, S. Fast Amide Couplings in Water: Extraction, Column Chromatography, and Crystallization Not Required. *Org. Lett.* **2020**, *22*, 5737–5740. https://doi.org/10.1021/acs.orglett.0c01676.

(9) Sheykhan, M.; Rashidi Ranjbar, Z.; Morsali, A.; Heydari, A. Minimisation of E-Factor in the Synthesis of *N*-Hydroxylamines: The Role of Silver(I)-Based Coordination Polymers. Green Chem. **2012**, *14*, 1971. https://doi.org/10.1039/c2gc35076a.

(10) Brals, J.; Smith, J. D.; Ibrahim, F.; Gallou, F.; Handa, S. Micelle-enabled palladium catalysis for convenient sp2-sp3 coupling of nitroalkanes with aryl bromides in water under mild conditions. *ACS Catal*. **2017**, *7*, 7245. https://doi.org/10.1021/acscatal.7b02663.

(11) Monteith, E. R.; Mampuys, P.; Summerton, L.; Clark, J. H.; Maes, B. U. W.; McElroy, C. R. Why We Might Be Misusing Process Mass Intensity (PMI) and a Methodology to Apply It Effectively as a Discovery Level Metric. Green Chem. **2020**, *22*, 123–135. https://doi.org/10.1039/C9GC01537J.

(12) Constable, D. J. C.; Curzons, A. D.; Cunningham, V. L. Metrics to ‘Green’ Chemistry—Which Are the Best? *Green Chem.* **2002**, *4*, 521–527. https://doi.org/10.1039/B206169B.

(13) Geissler, A.; Meiffren, G. Compositions in the form of an injectable aqueous solution comprising human glucagon and a copolyamino acid. WO2019110838A1, **2019**.

8. ANALYTICAL DATA

Dodecanoyl-*L*-proline (1b)[13]

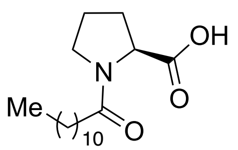

White waxy solid, yield 24.5 g (95%), R_f 0.2 (ethyl acetate). ^{1}H NMR (500 MHz, $CDCl_3$) δ 8.27 (s, 1H), 4.67 – 4.57 (m, 1H), 3.65 – 3.52 (m, 1H), 3.53 – 3.37 (m, 1H), 2.53 – 2.33 (m, 3H), 2.12 – 1.94 (m, 3H), 1.72 – 1.62 (m, 2H), 1.39 – 1.27 (m, 16H), 0.95 – 0.82 (m, 3H).

PS-750-M (1)[8]

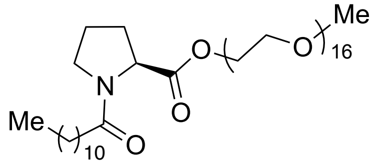

Waxy solid, yield 82.1 g (98%), R_f 0.2 (8:2, ethyl acetate/methanol).^{1}H NMR (400 MHz, $CDCl_3$) δ 4.65 – 4.39 (m, 1H), 4.31 – 4.19 (m, 1H), 3.58 – 3.73 (mPEG, m, 64-68H), 3.49 – 3.45 (m, 1H), 3.37 (s, Ome-PEG, 3H), 2.37 – 2.27 (m, 2H), 2.21– 2.05 (m, 2H), 2.02 – 1.86 (m, 2H), 1.68 – 1.59 (m, 2H), 1.34 – 1.22 (m, 16H), 0.87 (t, J = 6.6 Hz, 3H).

1-Naphthoic acid (2)[2]

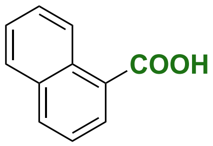

White solid, yield 39 mg (91%), R_f 0.51 (1:1, ethyl acetate: hexanes). ^{1}H NMR (300 MHz, $CDCl_3$) δ 9.10 (dq, J = 8.6, 0.9 Hz, 1H), 8.43 (dd, J = 7.3, 1.4 Hz, 1H), 8.15 – 8.07 (m, 1H), 7.93 (ddt, J = 8.1, 1.4, 0.6 Hz, 1H), 7.68 (ddd, J = 8.6, 6.8, 1.5 Hz, 1H), 7.63 – 7.51 (m, 2H).

6-Methoxynicotinic acid (3)[2]

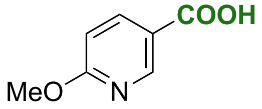

White solid, yield 28 mg (72%), R_f 0.2 (ethyl acetate). ^{1}H NMR (300 MHz, $CDCl_3$) δ 8.72 (dd, J = 2.5, 0.7 Hz, 1H), 8.14 (dd, J = 8.7, 2.4 Hz, 1H), 6.90 (dd, J = 8.6, 0.8 Hz, 1H), 3.92 (s, 3H).

2-Cyanobenzoic acid (4)[2]

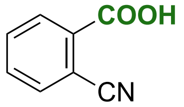

Off white solid, yield 28 mg (77%), R_f 0.36 (ethyl acetate). ^{1}H NMR (300 MHz, DMSO-d_6) δ 8.13 – 8.07 (m, 1H), 8.01 – 7.94 (m, 1H), 7.86 – 7.75 (m, 2H).

4-Methoxybenzoic acid (5)[2]

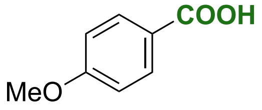

White solid, yield 28 mg (74%), R_f 0.49 (1:1, ethyl acetate: hexanes). ^{1}H NMR (300 MHz, $CDCl_3$) δ 8.11 – 8.01 (m, 2H), 6.99 – 6.90 (m, 2H), 3.88 (s, 3H).

9H-Fluorene-2-carboxylic acid (6)[2]

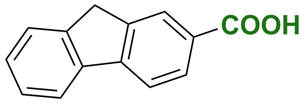

Brown solid, yield 41 mg (78%), R_f 0.3 (ethyl acetate). ^{1}H NMR (300 MHz, Acetone-d_6) δ 11.17 (s, 1H), 8.23 (dq, J = 1.7, 0.9 Hz, 1H), 8.09 (ddt, J = 7.9, 1.5, 0.8 Hz, 1H), 8.01 – 7.93 (m, 2H), 7.66 – 7.62 (m, 1H), 7.45 – 7.36 (m, 2H), 4.01 (s, 2H).

2-(2,2-Difluorobenzo[*d*][1,3]dioxol-5-yl)-2-(3-methoxyphenyl)acetonitrile (7)[3]

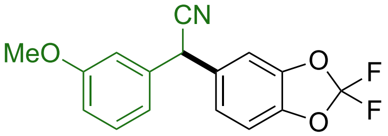

Yellow viscous liquid, yield 115 mg (76%) R_f 0.45 (1:9, ethyl acetate: hexanes). ^{1}H NMR (300 MHz, $CDCl_3$) δ 7.35 – 7.28 (m, 1H), 7.12 (ddd, J = 8.3, 1.8, 0.6 Hz, 1H), 7.08 – 7.03 (m, 1H), 7.03 (dt, J = 1.8, 0.5 Hz, 1H), 6.94 – 6.83 (m, 3H), 5.09 (s, 1H), 3.81 (s, 3H). ^{19}F NMR (282 MHz, $CDCl_3$) δ -49.78.

2-(4-Chlorophenyl)-2-(4-methoxyphenyl)acetonitrile (8)[3]

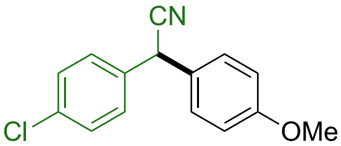

Yellow viscous liquid, yield 116 mg (90%), R_f 0.25 (1:9, ethyl acetate: hexanes). ^{1}H NMR (300 MHz, $CDCl_3$) δ 7.38 – 7.33 (m, 2H), 7.31 – 7.25 (m, 3H), 7.22 – 7.19 (m, 1H), 6.94 – 6.88 (m, 2H), 5.09 (s, 1H), 3.81 (s, 3H).

2-(Naphthalen-1-yl)-2-(*p*-tolyl)acetonitrile (9)[3]

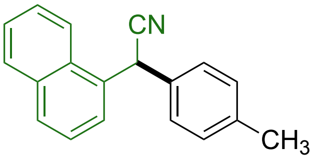

Viscous liquid, yield 106 mg (80%) R_f 0.22 (1:9, ethyl acetate: hexanes). ^{1}H NMR (300 MHz, $CDCl_3$) δ 7.94 – 7.84 (m, 3H), 7.70 – 7.62 (m, 1H), 7.57 – 7.46 (m, 3H), 7.24 (d, J = 7.9 Hz, 2H), 7.17 – 7.10 (m, 2H), 5.80 (s, 1H), 2.33 (s, 3H).

2-(4-Chlorophenyl)-2-(6-methoxypyridin-3-yl)acetonitrile (10)[3]

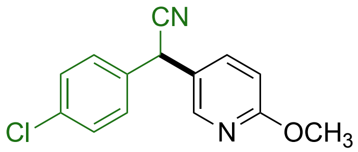

Viscous liquid, yield 117 mg (89%), R_f 0.5 (3:7, ethyl acetate: hexanes). ^{1}H NMR (300 MHz, $CDCl_3$) δ 8.10 (d, J = 2.6 Hz, 1H), 7.48 – 7.42 (m, 1H), 7.36 – 7.30 (m, 2H), 7.28 – 7.22 (m, 2H), 6.72 (dd, J = 8.7, 0.8 Hz, 1H), 5.05 (s, 1H), 3.90 (s, 3H).

2-([1,1'-Biphenyl]-4-yl)-2-(4-chlorophenyl)acetonitrile (11)[3]

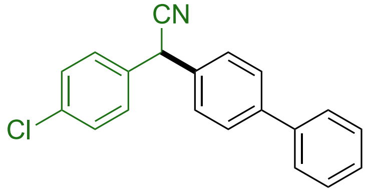

White solid, yield 109 mg (72%) R_f 0.25 (1:9, ethyl acetate: hexanes). ^{1}H NMR (500 MHz, $CDCl_3$) δ 7.62 – 7.58 (m, 2H), 7.58 – 7.54 (m, 2H), 7.45 (dd, J = 8.4, 6.8 Hz, 2H), 7.42 – 7.35 (m, 5H), 7.35 – 7.30 (m, 2H), 5.16 (s, 1H).

1-(4-Methoxyphenyl)naphthalene (12)[4]

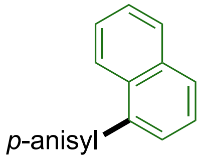

White solid, 52 mg (88%), R_f 0.42 (hexanes). ^{1}H NMR (300 MHz, $CDCl_3$) δ 7.92 (ddt, J = 8.0, 7.1, 0.9 Hz, 2H), 7.84 (d, J = 8.3 Hz, 1H), 7.54 – 7.40 (m, 6H), 7.04 (d, J = 8.7 Hz, 2H), 3.90 (s, 3H).

2-(Naphthalen-1-yl)thiophene (13)[4]

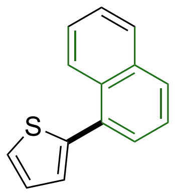

Light–yellow liquid, 42 mg (80%), R_f 0.39 (hexanes). ^{1}H NMR (300 MHz, $CDCl_3$) δ 8.25 – 8.19 (m, 1H), 7.93 – 7.83 (m, 2H), 7.58 (dd, J = 7.1, 1.4 Hz, 1H), 7.55 – 7.46 (m, 3H), 7.44 (dd, J = 5.1, 1.2 Hz, 1H), 7.26 – 7.21 (m, 1H), 7.20 – 7.17 (m, 1H).

2-(Benzo[*b*]thiophen-3-yl)benzonitrile (14)[4]

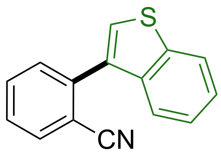

Light pink solid, 49 mg (83%), R_f 0.42 (1:9, ethyl acetate: hexanes). ^{1}H NMR (300 MHz, $CDCl_3$) δ 7.98 – 7.91 (m, 1H), 7.84 (ddd, J = 7.7, 1.4, 0.6 Hz, 1H), 7.74 – 7.61 (m, 4H), 7.51 (td, J = 7.5, 1.5 Hz, 1H), 7.46 – 7.37 (m, 2H).

1-(4'-Chloro-[1,1'-biphenyl]-4-yl)ethan-1-one (15)[4]

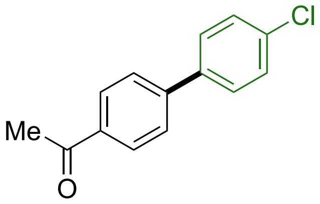

Light yellow solid, 56 mg (97%), R_f 0.2 (1:9, ethyl acetate: hexanes). ^{1}H NMR (400 MHz, $CDCl_3$) δ 8.03 (d, J = 8.4 Hz, 2H), 7.64 (d, J = 8.4 Hz, 2H), 7.55 (d, J = 8.6 Hz, 2H), 7.44 (d, J = 8.5 Hz, 2H), 2.64 (s, 3H).

4-Fluoro-2-(naphthalen-1-yl)benzaldehyde (16)[4]

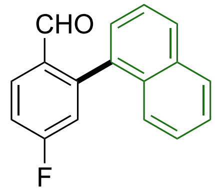

Colorless viscous liquid, 58 mg (93%), R_f 0.42 (1:9 ethyl acetate: hexanes). ^{1}H NMR (300 MHz, $CDCl_3$) *z* 9.54 (s, 1H), 8.16 (dd, *J* = 8.7, 5.9 Hz, 1H), 8.03 – 7.92 (m, 2H), 7.63 – 7.39 (m, 5H), 7.34 – 7.24 (m, 1H), 7.17 (dd, *J* = 9.0, 2.6 Hz, 1H).

(*E*)-1-styryl-4-(trifluoromethyl)benzene (17)[5]

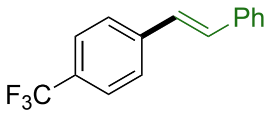

White solid, yield 63 mg (90%), R_f 0.76 (9:1, hexanes/ethyl acetate). ^{1}H NMR (300 MHz, $CDCl_3$) δ 7.61 (s, 4H), 7.57 – 7.51 (m, 2H), 7.36 – 7.41 (m, 2H), 7.34 – 7.27 (m, 1H), 7.20 (d, *J* = 16.4 Hz, 1H), 7.12 (d, *J* = 16.3 Hz, 1H). ^{19}F NMR (282 MHz, $CDCl_3$) δ -60.87.

(*E*)-1,2,3,4,5-pentafluoro-6-(2-methoxystyryl)benzene (18)[5]

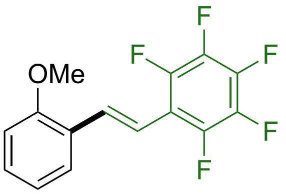

White solid, yield 59 mg (79%), R_f 0.30 (hexanes). ^{1}H NMR (300 MHz, $CDCl_3$) δ 7.76 (d, *J* = 16.9 Hz, 1H), 7.57 (dd, *J* = 7.8, 1.7 Hz, 1H), 7.36 – 7.27 (m, 1H), 7.06 – 6.90 (m, 3H), 3.90 (s, 3H). ^{19}F NMR (282 MHz, $CDCl_3$) δ -142.76 – -143.05 (m, 2F), -157.22 (t, *J* = 20.8 Hz, 1F), -163.30 (td, *J* = 21.5, 7.8 Hz, 2F).

(*E*)-2-styrylthiophene (19)[5]

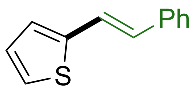

White solid, yield 33 mg (71%), R$_f$ 0.36 (1:9, ethyl acetate/ hexanes). ^{1}H NMR (300 MHz, $CDCl_3$) δ 7.51 – 7.43 (m, 2H), 7.36 – 7.31 (m, 2H), 7.27 – 7.25 (m, 1H), 7.20 – 7.18 (m, 2H), 7.07 – 7.06 (m, 1H), 7.02 – 6.99 (m, 1H), 6.93 (d, J = 16.1 Hz, 1H).

Methyl (*E*)-3-(2-nitrophenyl)acrylate (20)[5]

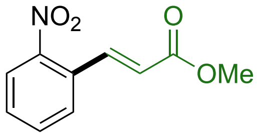

Yellowish white solid, yield 47 mg (81%), R$_f$ 0.3 (3:7, ethyl acetate/hexanes). ^{1}H NMR (300 MHz, $CDCl_3$) δ 8.18 – 7.99 (m, 2H), 7.71 – 7.59 (m, 2H), 7.59 – 7.49 (m, 1H), 6.37 (d, J = 15.8 Hz, 1H), 3.83 (s, 3H).

***N*-(*p*-Tolyl)naphthalen-1-amine (21)[6]**

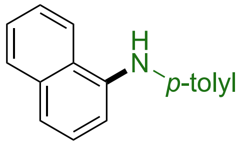

Pale yellow oil, yield 59 mg (85%), R$_f$ 0.4 (9:1, hexanes/ethyl acetate). ^{1}H NMR (300 MHz, $CDCl_3$) δ 8.08 – 8.00 (m, 1H), 7.93 – 7.85 (m, 1H), 7.60 – 7.45 (m, 3H), 7.40 (td, J = 7.9, 3.4 Hz, 1H), 7.36 – 7.29 (m, 1H), 7.12 (dd, J = 8.5, 2.9 Hz, 2H), 7.04 – 6.94 (m, 2H), 5.92 (s, 1H), 2.34 (s, 3H).

4-(1*H*-Indol-1-yl)benzonitrile (22)[6]

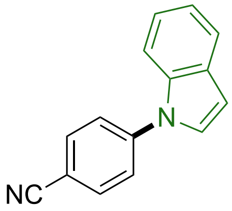

Brown solid, yield 49 mg (74%), R$_f$ 0.3 (9:1, hexanes/ethyl acetate). ^{1}H NMR (300 MHz, $CDCl_3$) δ 7.86 – 7.78 (m, 2H), 7.74 – 7.58 (m, 4H), 7.35 (d, J = 3.4 Hz, 1H), 7.31 – 7.17 (m, 2H), 6.75 (dd, J = 3.3, 0.8 Hz, 1H).

4-(Naphthalen-1-yl)morpholine (23)[6]

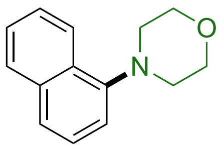

Yellow liquid, yield 49 mg (76%), R_f 0.4 (9:1, hexanes/ethyl acetate). ^{1}H NMR (300 MHz, $CDCl_3$) δ 8.23 (d, J = 9.4 Hz, 1H), 7.87 – 7.81 (m, 1H), 7.58 (d, J = 8.2 Hz, 1H), 7.53 – 7.39 (m, 3H), 7.11 (d, J = 7.3 Hz, 1H), 4.06 – 3.90 (m, 4H), 3.14 (t, J = 4.6 Hz, 4H).

4-Chloro-*N*-(*p*-tolyl)aniline (24)[6]

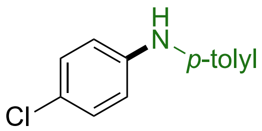

Yellow solid, yield 57 mg (87%), R_f 0.3 (9:1, hexanes/ethyl acetate). ^{1}H NMR (300 MHz, $CDCl_3$) δ 7.21 – 7.14 (m, 2H), 7.13 – 7.06 (m, 2H), 7.01 – 6.88 (m, 4H), 5.58 (s, 1H), 2.31 (s, 3H).

10-(3,5-*Bis*(trifluoromethyl)phenyl)-10*H*-phenothiazine (25)[6]

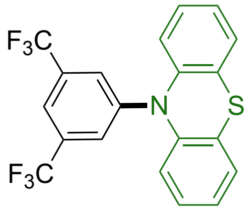

White solid, yield 98 mg (79%), R_f 0.3 (9:1, hexanes/ethyl acetate). ^{1}H NMR (300 MHz, $CDCl_3$) δ 7.57 (d, J = 12.0 Hz, 3H), 7.37 (dd, J = 7.6, 1.6 Hz, 2H), 7.30 – 7.05 (m, 4H), 7.01 – 6.91 (m, 2H). ^{19}F NMR (282 MHz, $CDCl_3$) δ -62.99.

4-(1-Naphthoyl)benzaldehyde (26)[7]

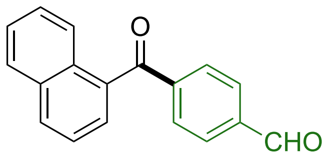

Yellow solid, yield 38 mg (58%), R_f 0.5 (2:8, ethyl acetate/hexane). ^{1}H NMR (500 MHz, $CDCl_3$) δ 10.13 (s, 1H), 8.17 – 8.14 (m, 1H), 8.05 (dt, J = 8.2, 1.2 Hz, 1H), 8.01 – 7.93 (m, 5H), 7.59 – 7.51 (m, 4H).

1-Naphthalenyl-2-thienylmethanone (27)[7]

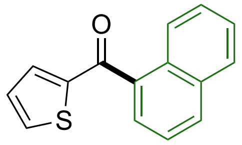

Viscous liquid, yield 48 mg (81%), R_f 0.5 (1:9, ethyl acetate/hexane). ^{1}H NMR (300 MHz, $CDCl_3$) δ 8.23 – 8.14 (m, 1H), 8.01 (dt, J = 8.3, 1.1 Hz, 1H), 7.97 – 7.87 (m, 1H), 7.80 – 7.70 (m, 2H), 7.60 – 7.46 (m, 4H), 7.11 (dd, J = 4.9, 3.8 Hz, 1H).

4-*tert*-Butylbenzophenone (28)[7]

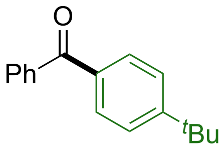

Viscous liquid, yield 44 mg (75%), R_f 0.8 (2:8, ethyl acetate/hexane). ^{1}H NMR (300 MHz, $CDCl_3$) δ 7.84 – 7.74 (m, 4H), 7.63 – 7.54 (m, 1H), 7.54 – 7.43 (m, 4H), 1.37 (s, 9H).

***Tert*-butyl (4-(thiophene-2-carbonyl)phenyl)carbamate (29)**[7]

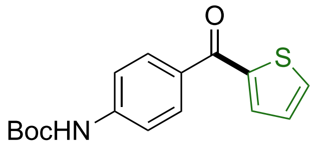

White solid, yield 54 mg (71%), R_f 0.5 (2:8, ethyl acetate/hexane). ^{1}H NMR (400 MHz, $CDCl_3$) δ 7.91 – 7.84 (m, 2H), 7.69 (dd, J = 5.0, 1.1 Hz, 1H), 7.64 (dd, J = 3.7, 1.2 Hz, 1H), 7.54 – 7.46 (m, 2H), 7.16 (dd, J = 4.9, 3.7 Hz, 1H), 6.74 (s, 1H), 1.54 (s, 9H).

_N_-(1-phenylethyl)benzamide (30)[8]

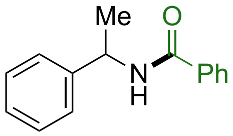

White solid, yield 50 mg (89%), R_f 0.36 (8:2, hexanes/ethyl acetate). ^{1}H NMR (300 MHz, $CDCl_3$) δ 7.81 – 7.74 (m, 2H), 7.53 – 7.46 (m, 1H), 7.48 – 7.32 (m, 6H), 7.32 – 7.27 (m, 1H), 6.30 (s, 1H), 5.35 (quint, J = 7.0 Hz, 1H), 1.62 (d, J = 6.9 Hz, 3H).

Methyl benzoyl-_L_-phenylalaninate (31)[8]

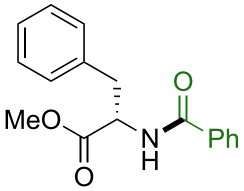

Light pink solid, yield 62 mg (88%), R_f 0.32 (7:3, hexanes/ethyl acetate). ^{1}H NMR (300 MHz, $CDCl_3$) δ 7.78 – 7.68 (m, 2H), 7.56 – 7.47 (m, 1H), 7.46 – 7.37 (m, 2H), 7.35 – 7.22 (m, 3H), 7.19 – 7.08 (m, 2H), 6.58 (d, J = 7.6 Hz, 1H), 5.10 (dt, J = 7.6, 5.6 Hz, 1H), 3.77 (s, 3H), 3.36 – 3.17 (m, 2H).

Methyl((_R_)-3-(4-((((2-bromobenzyl)oxy)carbonyl)oxy)phenyl)-2-((_tert_-butoxycarbonyl) amino)propanoyl)-_L_-phenylalaninate (32)[8]

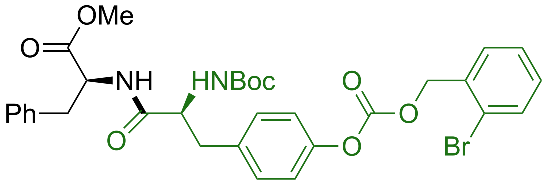

White solid, yield 41 mg (90%), R_f 0.34 (9:1, dichloromethane/ methanol). ^{1}H NMR (500 MHz, $CDCl_3$) δ 7.63 (dd, J = 8.1, 1.2 Hz, 1H), 7.52 (dd, J = 7.7, 1.7 Hz, 1H), 7.40 – 7.35 (m, 1H), 7.30

– 7.21 (m, 6H), 7.17 – 7.12 (m, 2H), 7.07 – 7.02 (m, 2H), 6.35 (d, *J* = 7.7 Hz, 1H), 5.38 (s, 2H), 4.98 (s, 1H), 4.80 (q, *J* = 6.5 Hz, 1H), 4.35 (s, 1H), 3.70 (s, 3H), 3.16 – 3.00 (m, 4H), 1.43 (s, 9H).

_N_-(3,5-dimethylphenyl)-2-(4-hydroxyphenyl)acetamide (33)[8]

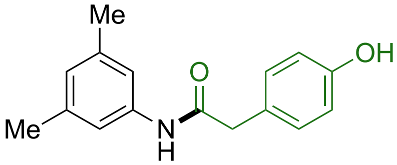

Light yellow solid, yield 47 mg (74%), R_f 0.4 (6:4, hexanes/ethyl acetate). ^{1}H NMR (300 MHz, DMSO) δ 9.88 (s, 1H), 9.24 (s, 1H), 7.20 (d, *J* = 1.5 Hz, 2H), 7.11 (d, *J* = 8.4 Hz, 2H), 6.72 – 6.65 (m, 3H), 3.46 (s, 2H), 2.21 (s, 6H)

Methyl _N_-(((9_H_-fluoren-9-yl)methoxy)carbonyl)-_N_^{2}-(_tert_-butoxycarbonyl)-_L_-lysyl-_L_-phenylalaninate (34)[8]

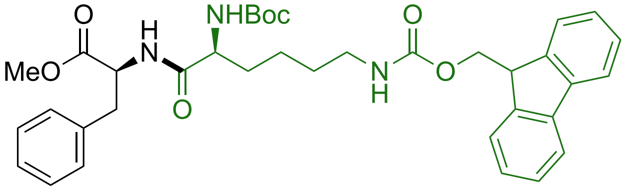

Pale yellow solid, yield 36 mg (82%), R_f 0.5 (9:1, dichloromethane/ methanol). ^{1}H NMR (500 MHz, $CDCl_3$) δ 7.75 (dd, *J* = 11.5, 7.3 Hz, 2H), 7.57 (d, *J* = 7.1 Hz, 2H), 7.39 (q, *J* = 5.9 Hz, 2H), 7.34 – 7.27 (m, 2H), 7.25 – 7.13 (m, 3H), 7.10 (d, *J* = 8.2 Hz, 2H), 6.62 (s, 1H), 5.48 (s, 1H), 4.86 (d, *J* = 9.8 Hz, 1H), 4.58 (s, 1H), 4.35 (t, *J* = 7.6 Hz, 2H), 4.17 (d, *J* = 11.3 Hz, 2H), 3.69 (d, *J* = 11.3 Hz, 3H), 3.21 – 2.95 (m, 4H), 1.74 (s, 2H), 1.56 – 1.42 (m, 11H), 1.24 (s, 2H).

9. NMR SPECTRA

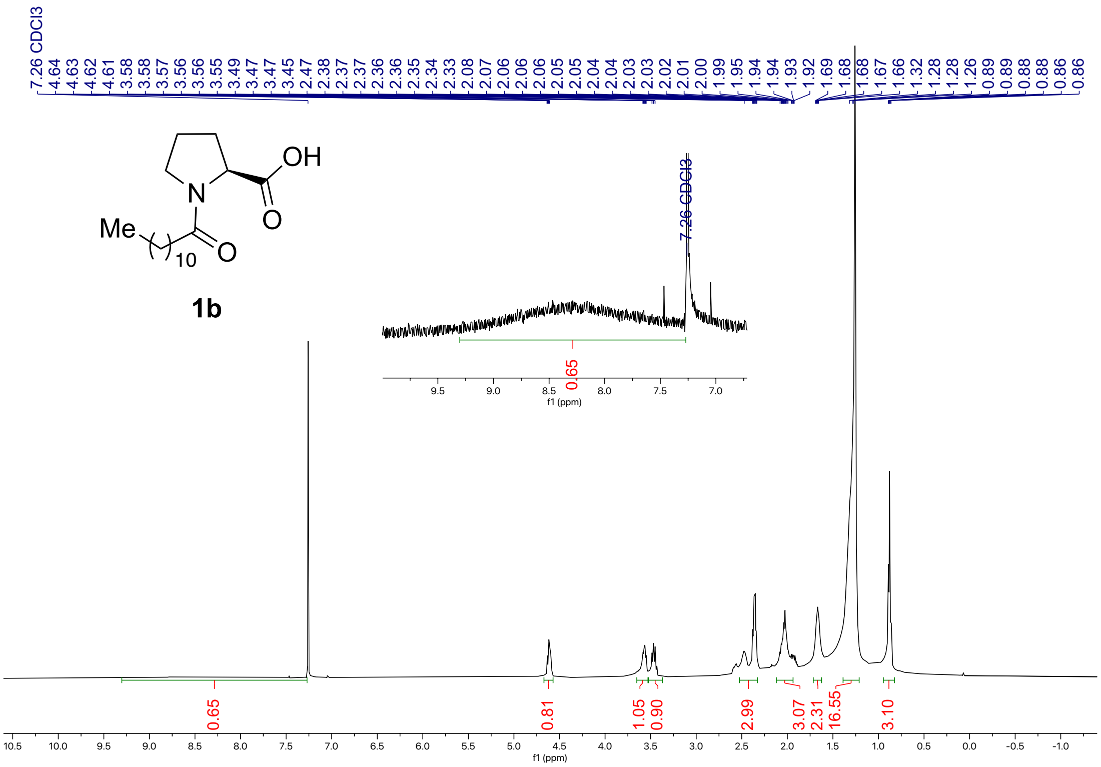

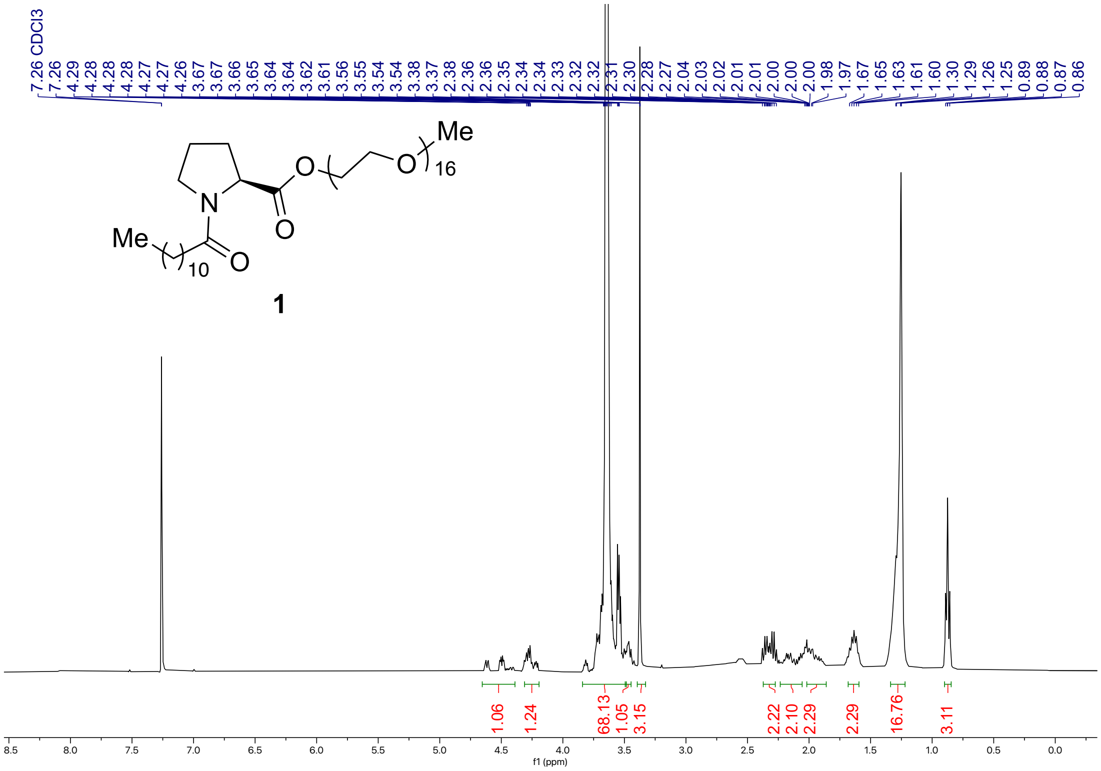

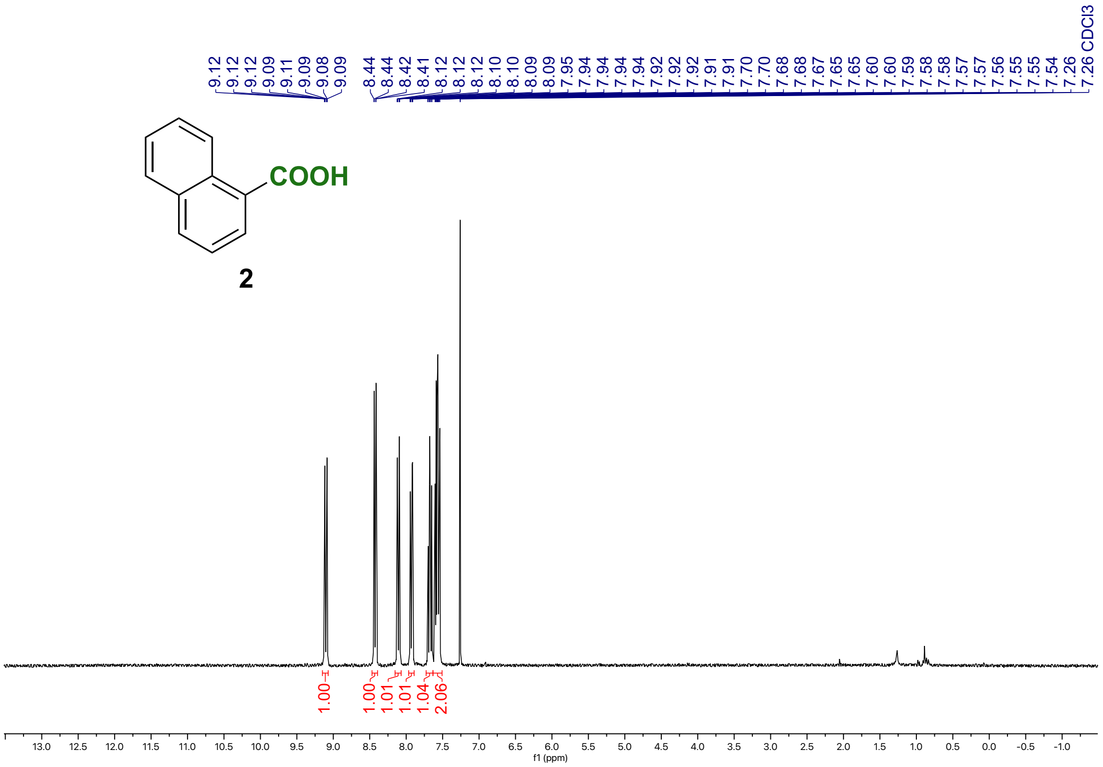
9.12
9.12
9.12
9.09
9.11
9.09
9.08
9.09
8.44
8.44
8.42
8.41
8.12
8.12
8.10
8.09
8.09
7.95
7.94
7.94
7.94
7.92
7.92
7.92
7.91
7.91
7.70
7.70
7.68
7.68
7.67
7.65
7.65
7.60
7.60
7.59
7.58
7.58
7.57
7.57
7.56
7.55
7.55
7.54
7.26
7.26 CDCl3
COOH
2
1.00
1.00
1.01
1.01
1.04
2.06
f1 (ppm)

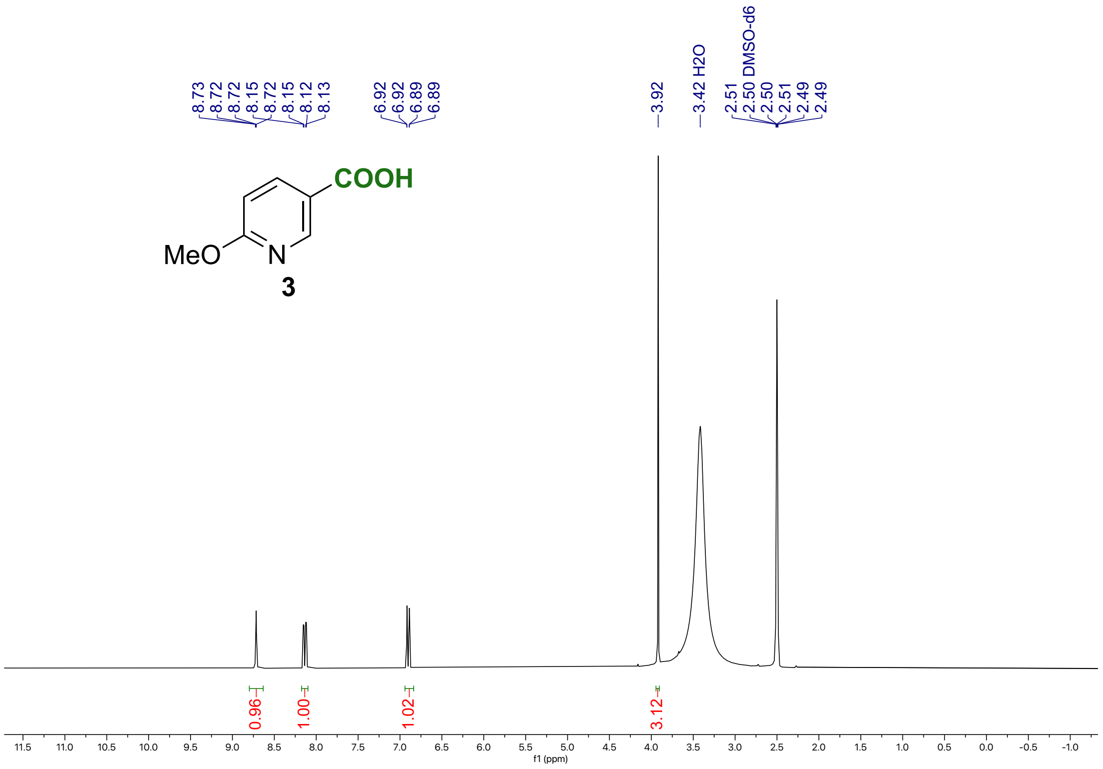
8.73
8.72
8.72
8.15
8.72
8.15
8.12
8.13
6.92
6.92
6.89
6.89
3.92
3.42 H2O
2.51
2.50 DMSO-d6
2.50
2.51
2.49
2.49
COOH
MeO
N
3
0.96
1.00
1.02
3.12
f1 (ppm)

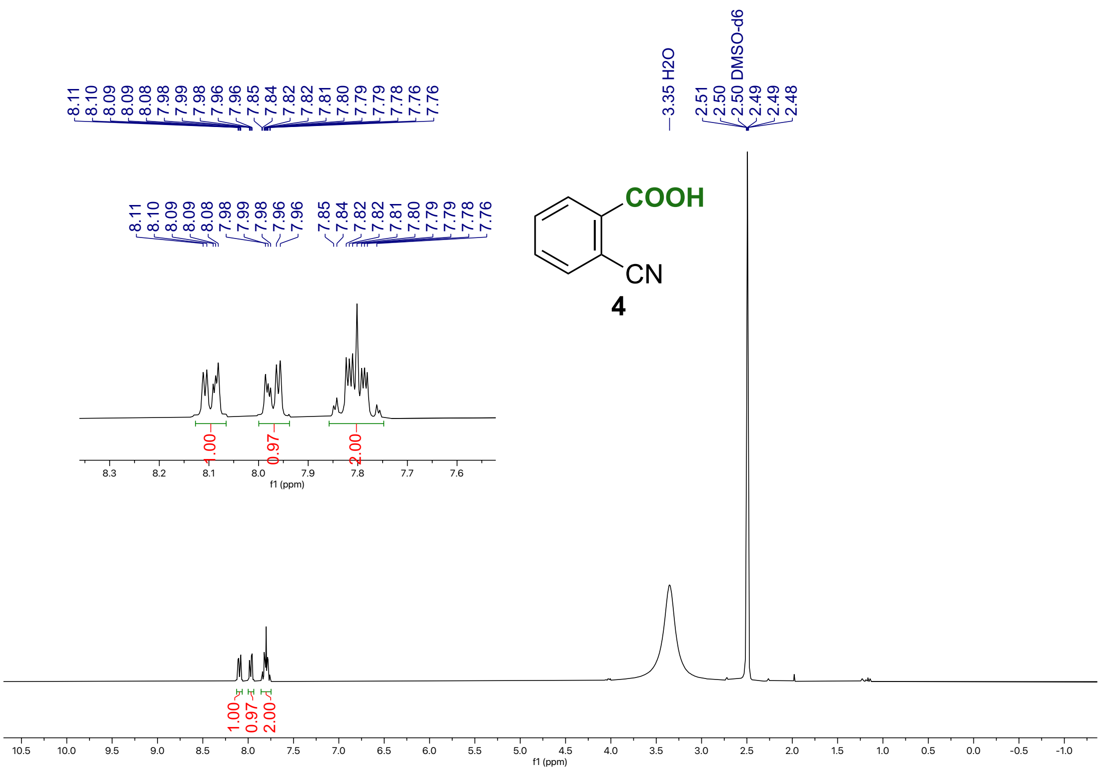
8.11
8.10
8.09
8.08
7.98
7.99
7.98
7.96
7.96
7.85
7.84
7.82
7.82
7.81
7.80
7.79
7.79
7.78
7.76
7.76
3.35 H2O
2.51
2.50
2.50 DMSO-d6
2.49
2.49
2.48
COOH
CN
4
1.00
0.97
2.00
f1 (ppm)

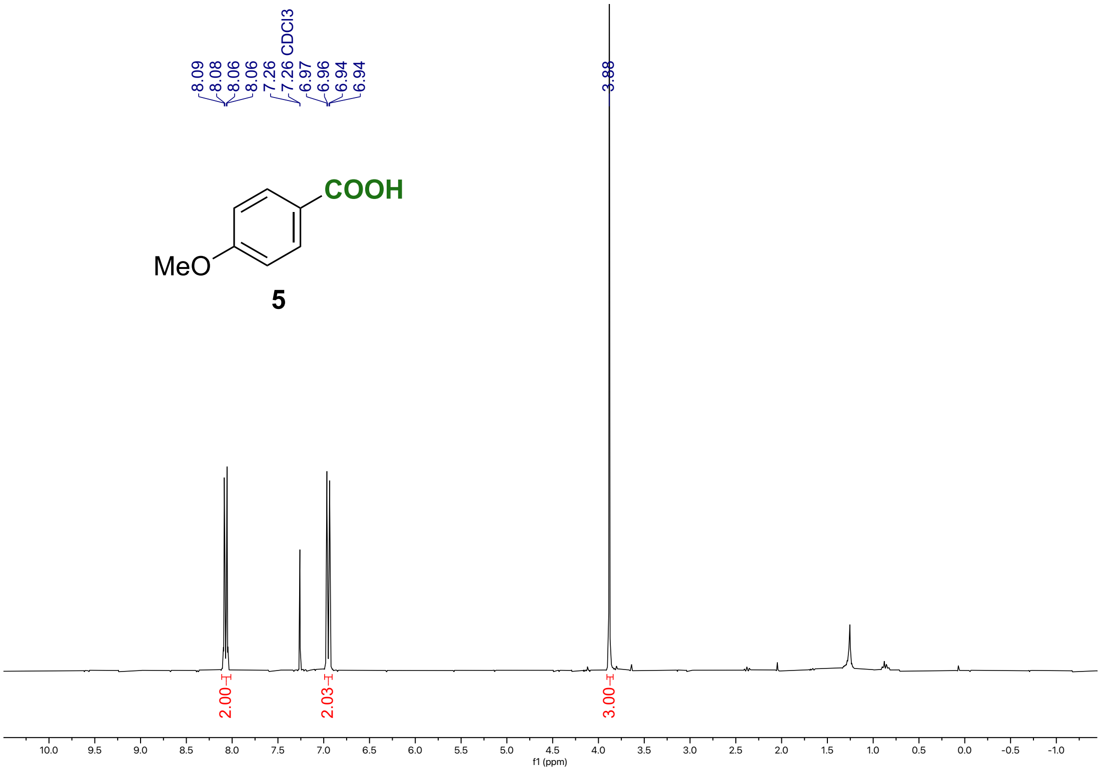
8.09
8.08
8.06
8.06
7.26
7.26 CDCl3
6.97
6.96
6.94
6.94
3.88
COOH
MeO
5
2.00
2.03
3.00
f1 (ppm)

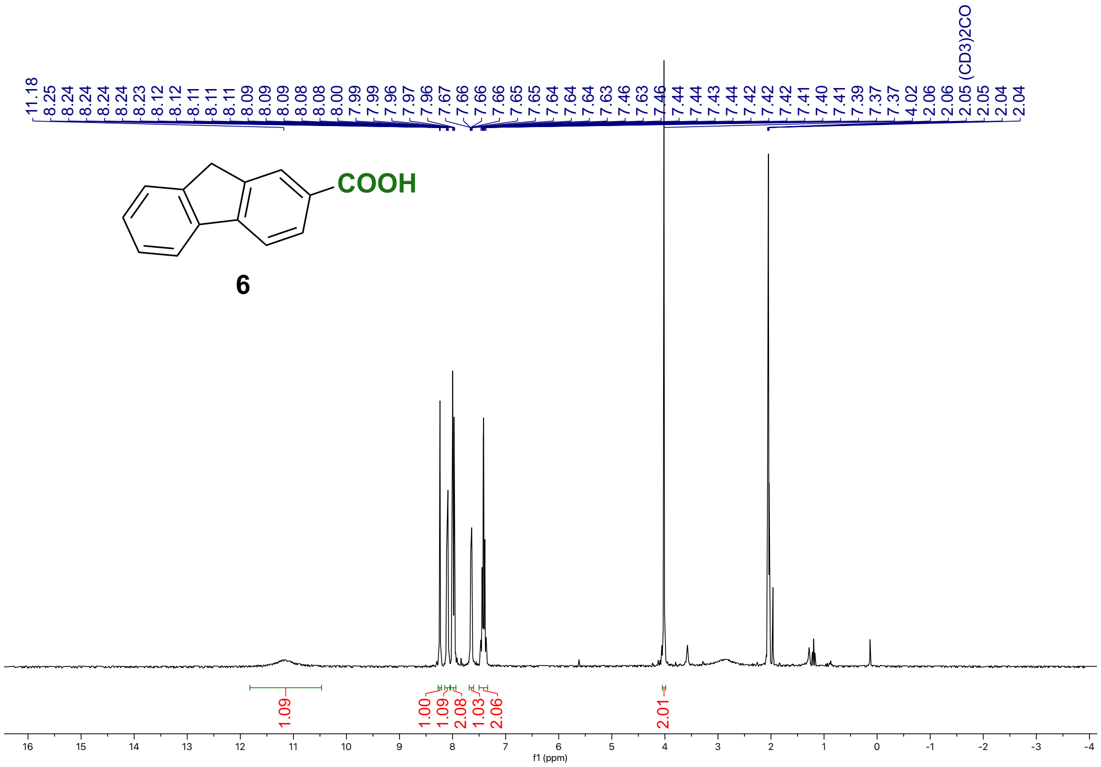
11.18 8.25 8.24 8.24 8.24 8.23 8.12 8.12 8.11 8.11 8.09 8.09 8.09 8.08 8.00 7.99 7.96 7.97 7.96 7.67 7.66 7.66 7.66 7.65 7.64 7.64 7.63 7.63 7.46 7.44 7.44 7.43 7.44 7.42 7.42 7.42 7.41 7.40 7.41 7.39 7.37 7.37 4.02 2.06 2.06 2.05 (CD3)2CO 2.05 2.04 2.04
COOH
6
1.09
1.00
1.09
2.08
1.03
2.06
2.01
f1 (ppm)

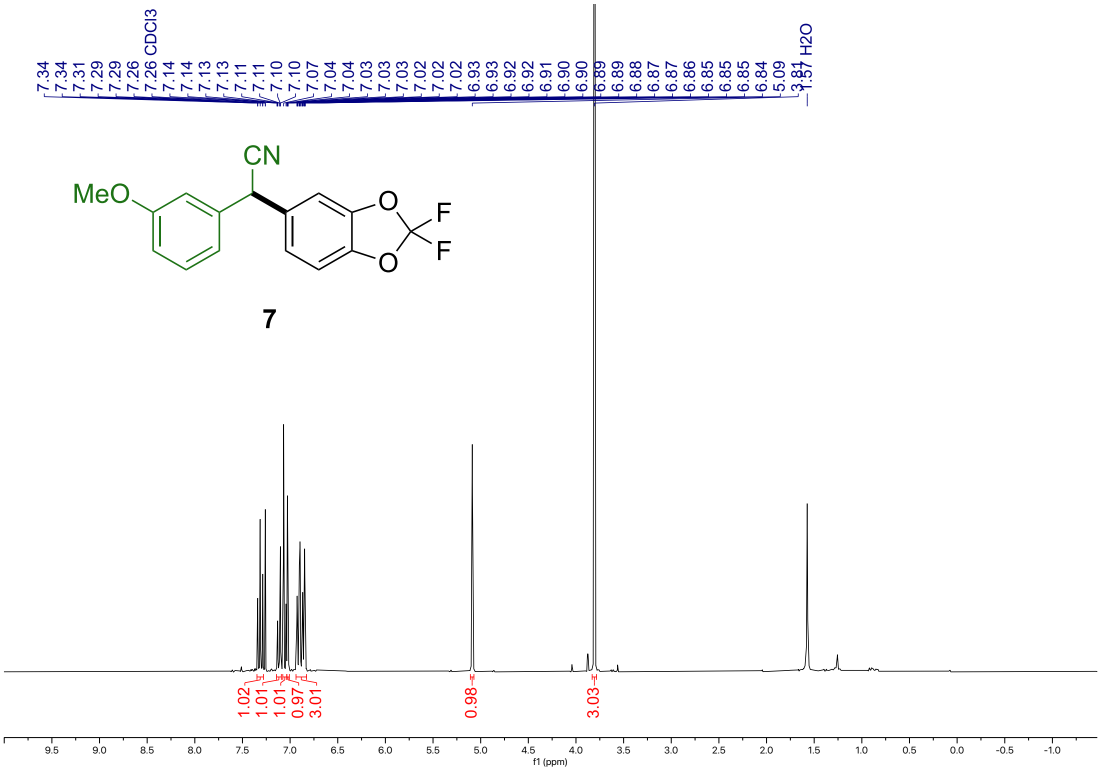
7.34 7.34 7.31 7.29 7.29 7.26 CDCl3 7.14 7.14 7.13 7.13 7.11 7.11 7.10 7.07 7.04 7.04 7.03 7.03 7.03 7.02 7.02 7.02 6.93 6.93 6.92 6.92 6.91 6.90 6.90 6.89 6.89 6.88 6.87 6.86 6.85 6.85 6.84 5.09 3.81 1.57 H2O
CN
MeO
O
O
F
F
7
1.02
1.01
1.01
0.97
3.01
0.98
3.03
f1 (ppm)

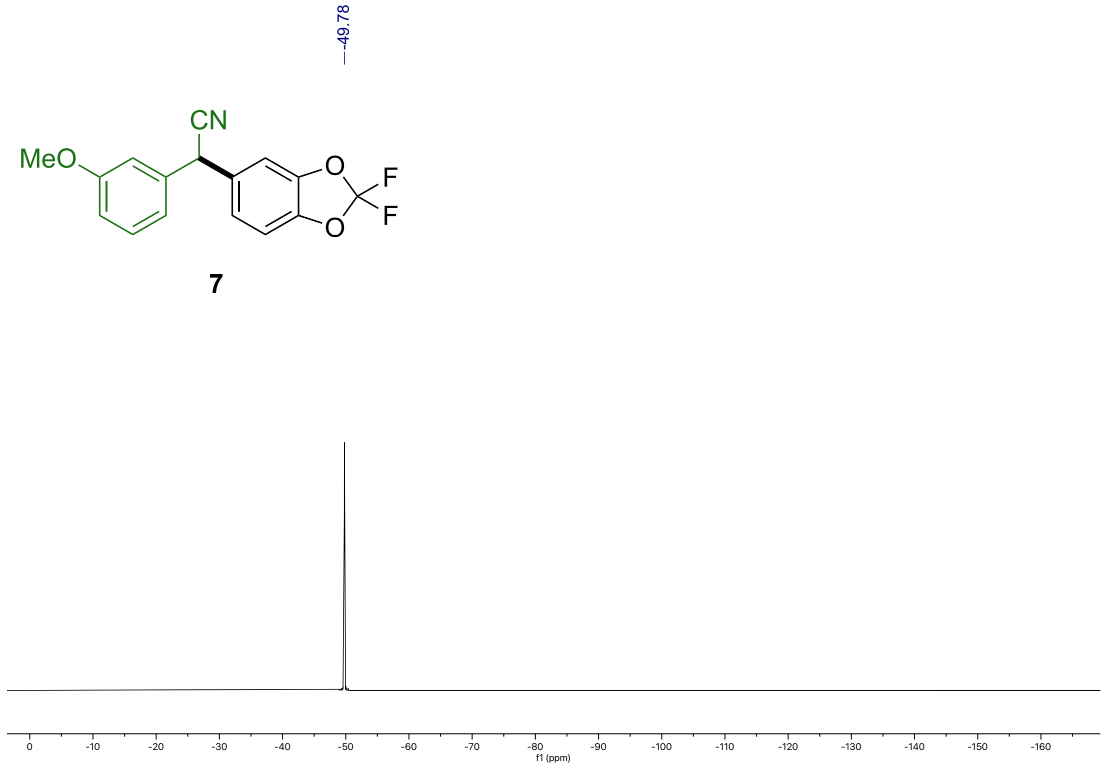

7

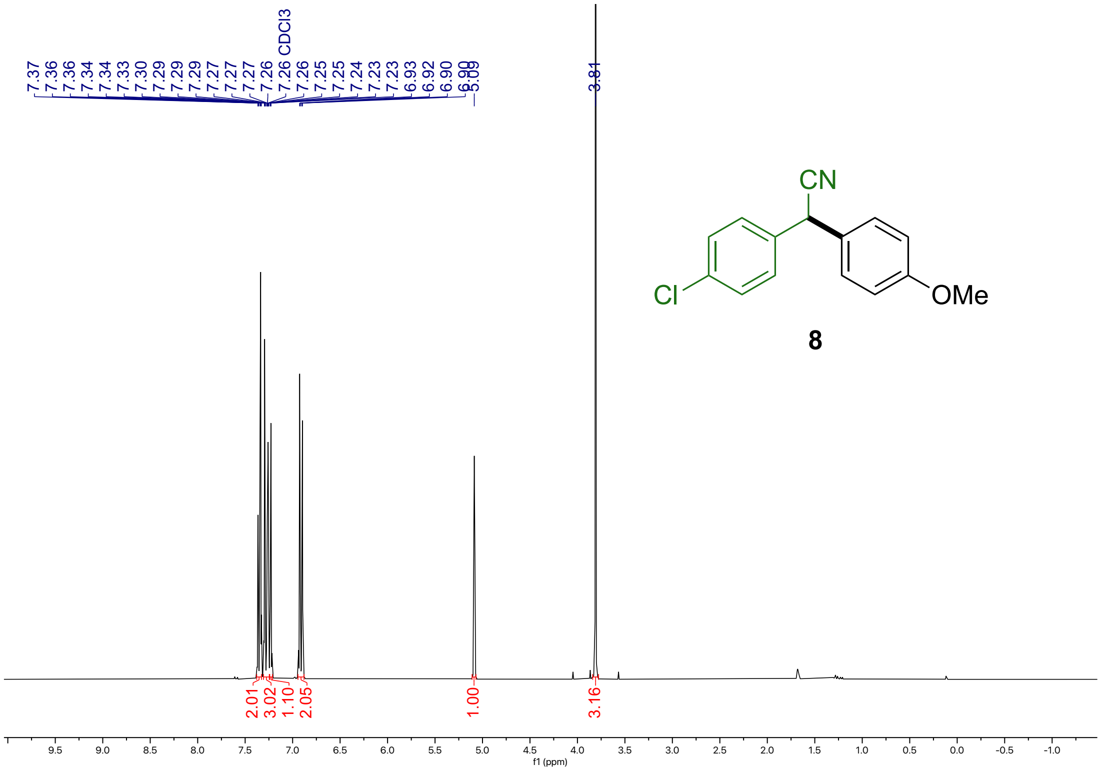

8

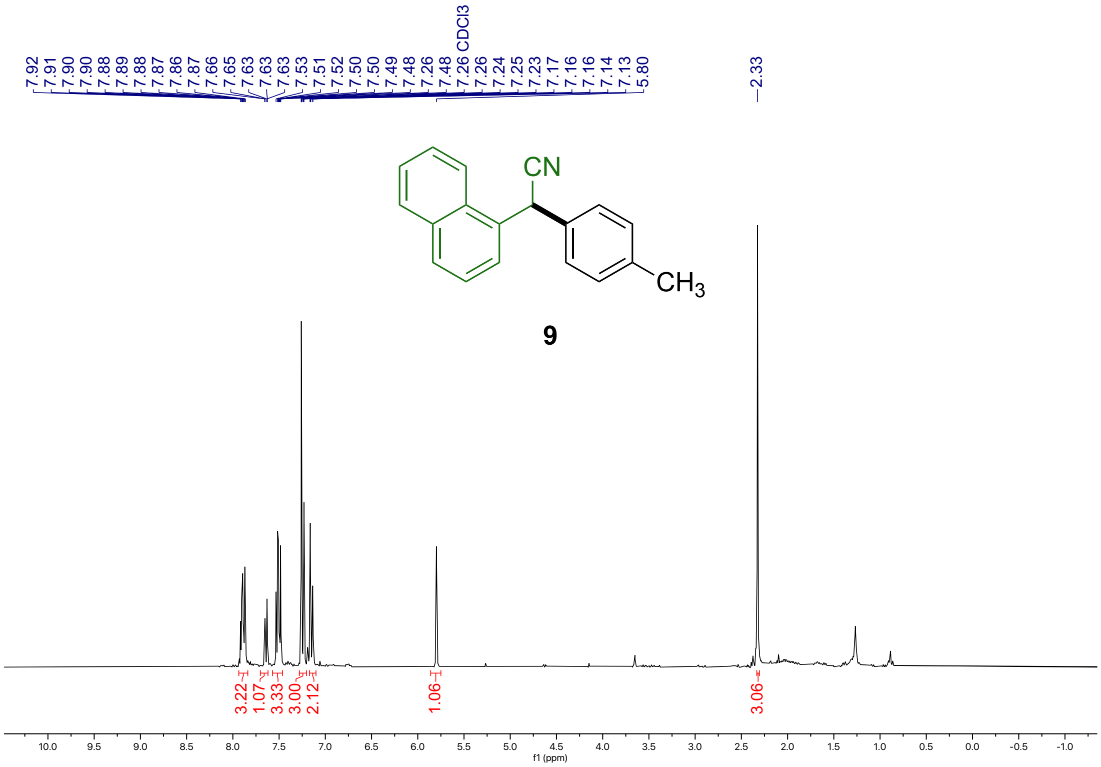

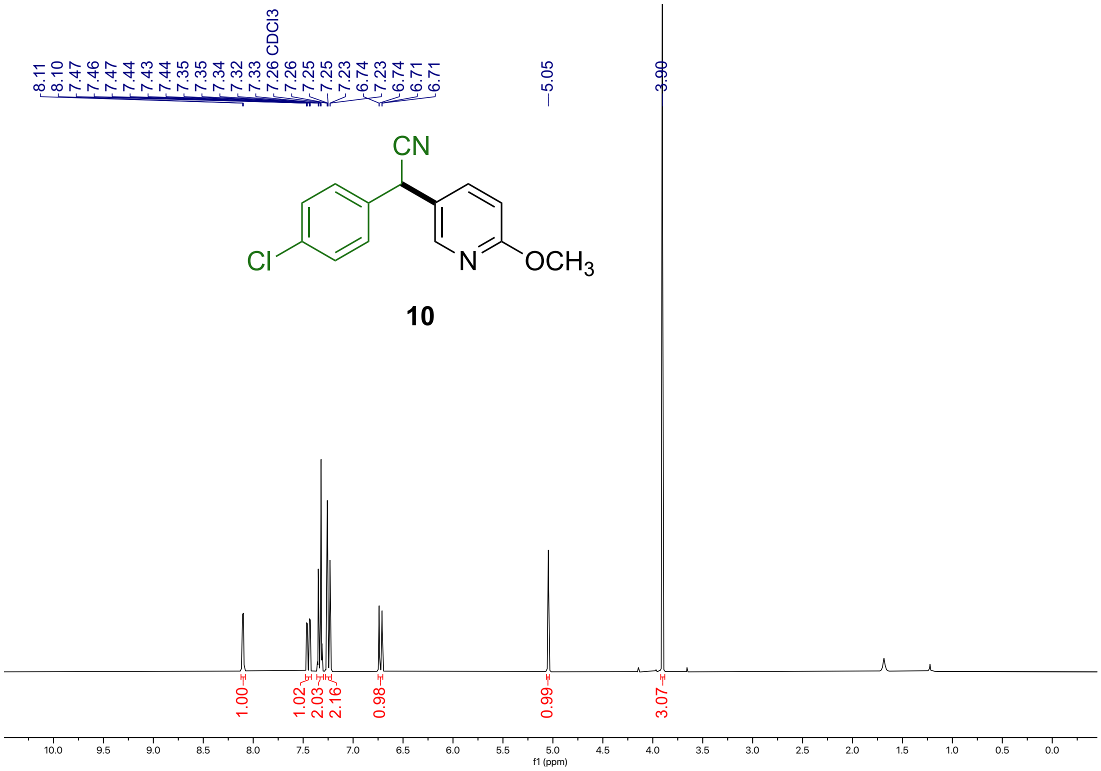

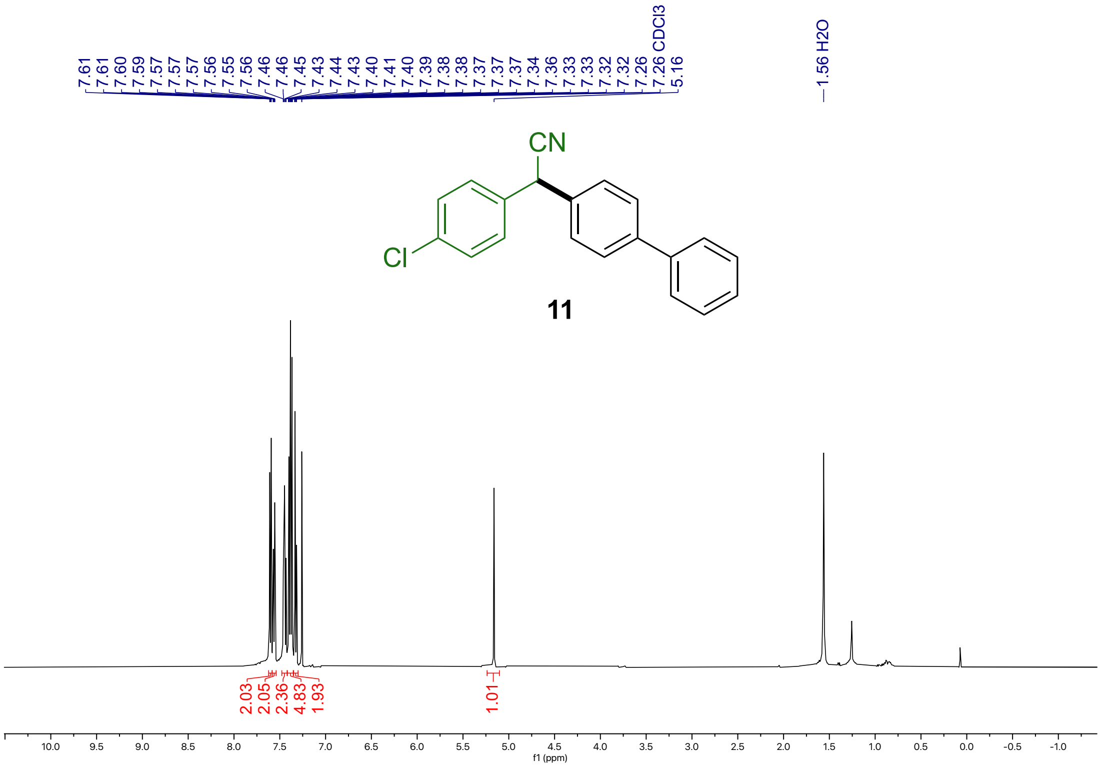
7.61
7.61
7.60
7.59
7.57
7.57
7.56
7.55
7.56
7.46
7.45
7.43
7.44
7.43
7.40
7.41
7.40
7.39
7.38
7.38
7.37
7.37
7.37
7.34
7.36
7.33
7.33
7.32
7.32
7.26
7.26 CDCl3
5.16
1.56 H2O
CN
Cl
11
2.03
2.05
2.36
4.83
1.93
1.01
f1 (ppm)

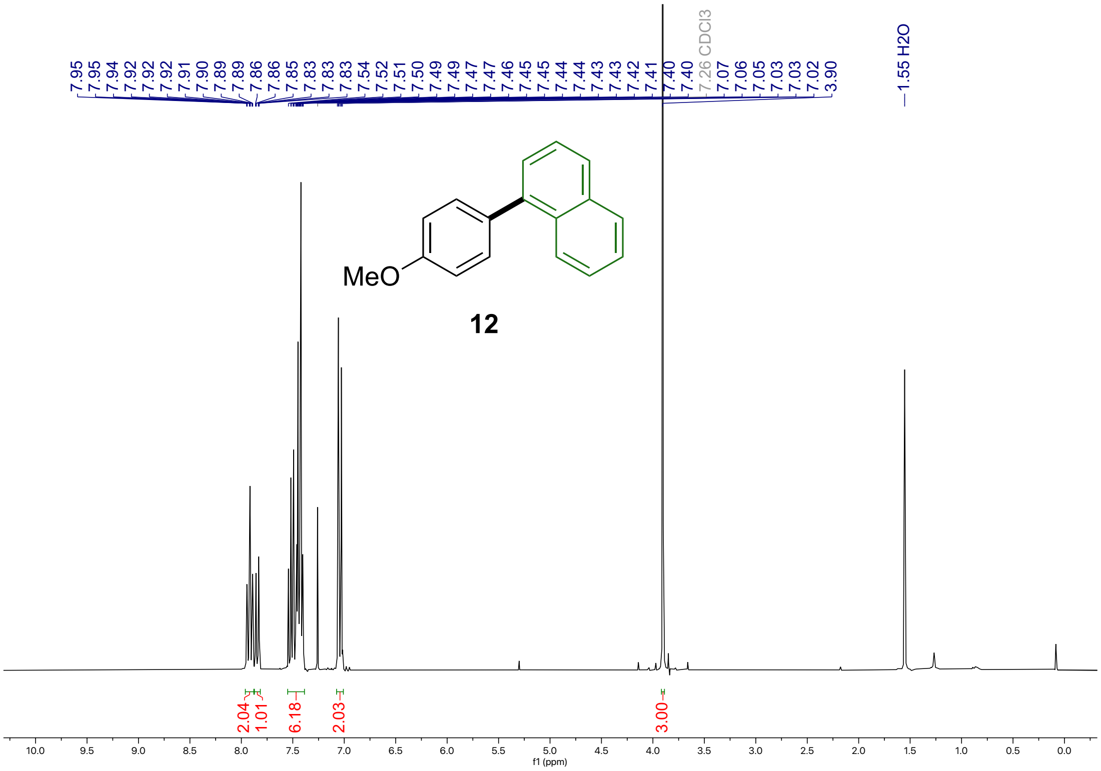
7.95
7.95
7.94
7.92
7.92
7.91
7.90
7.89
7.86
7.86
7.85
7.83
7.83
7.54
7.52
7.51
7.50
7.49
7.47
7.47
7.46
7.45
7.45
7.44
7.43
7.42
7.41
7.40
7.40
7.26 CDCl3
7.07
7.06
7.05
7.03
7.03
7.02
3.90
1.55 H2O
MeO
12
2.04
1.01
6.18
2.03
3.00
f1 (ppm)

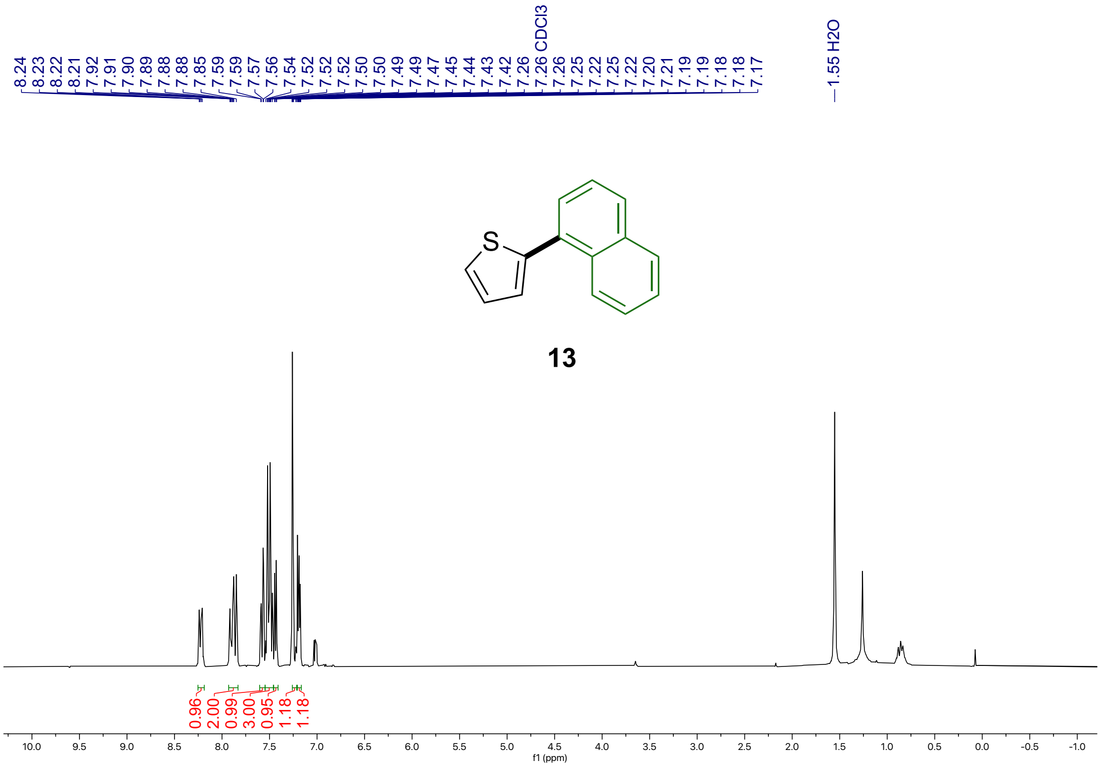

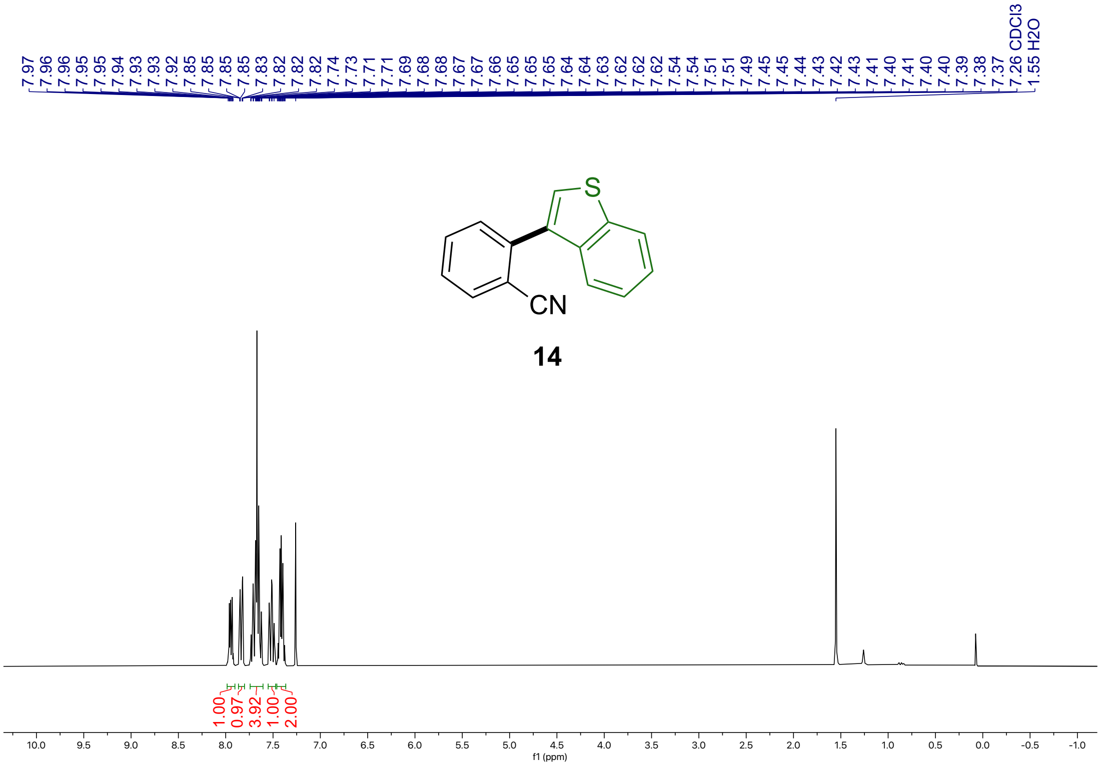

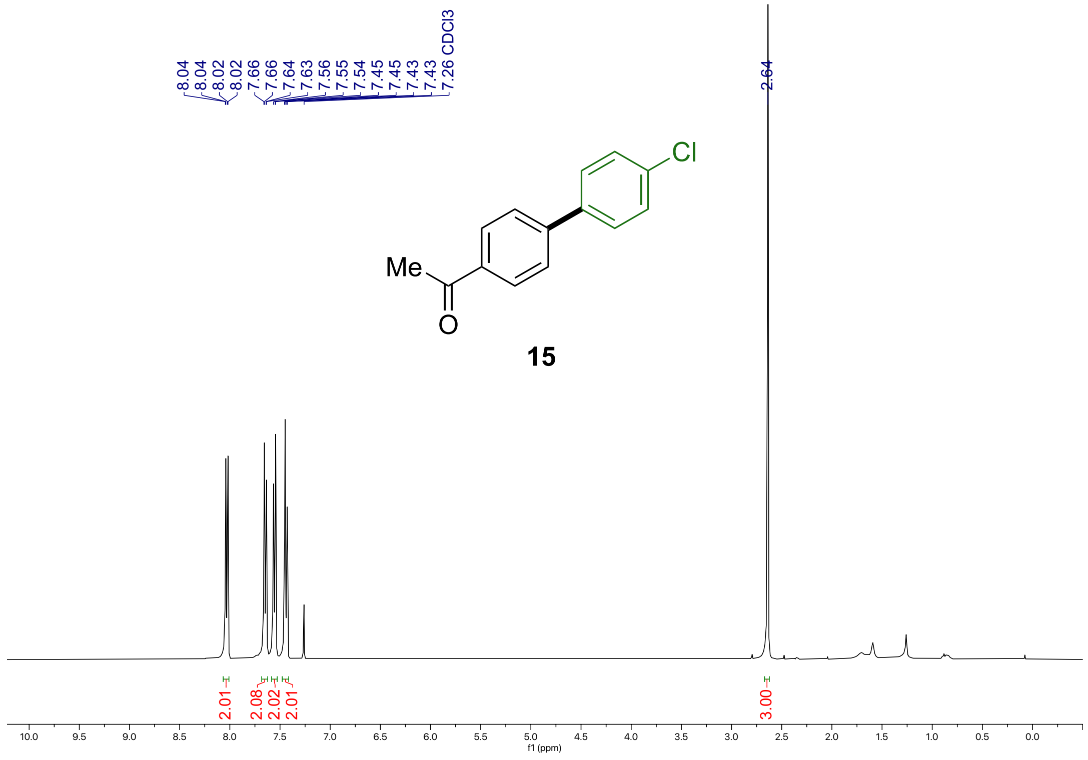

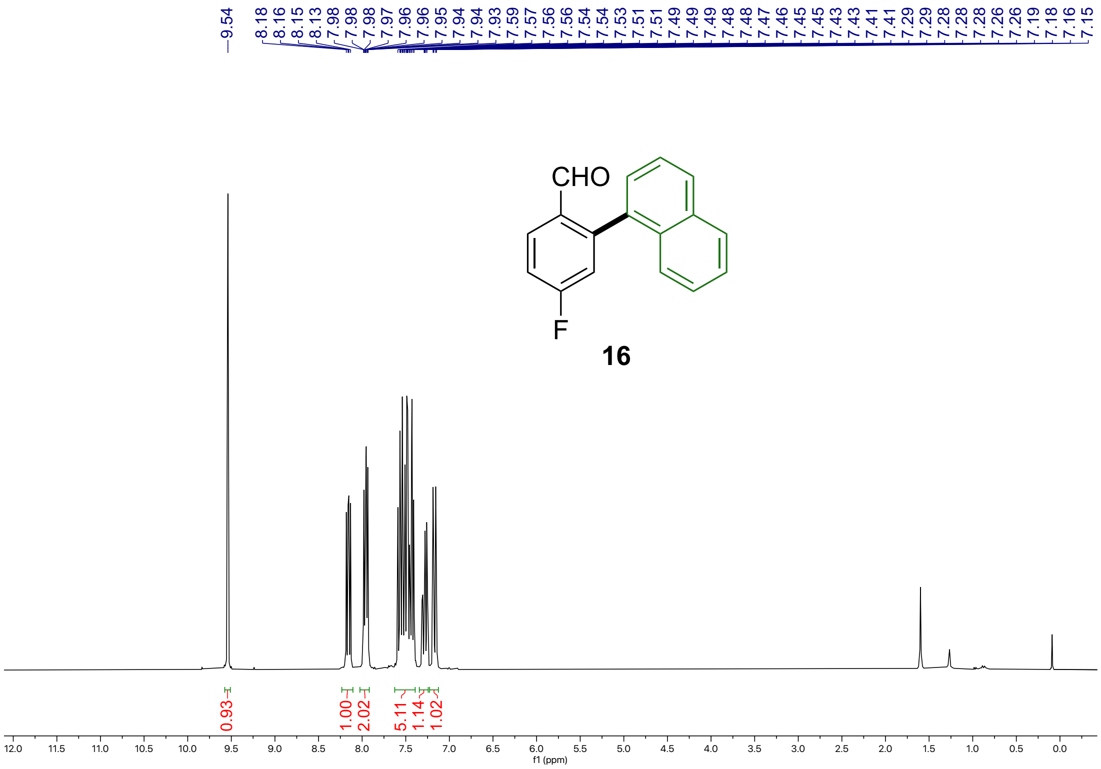

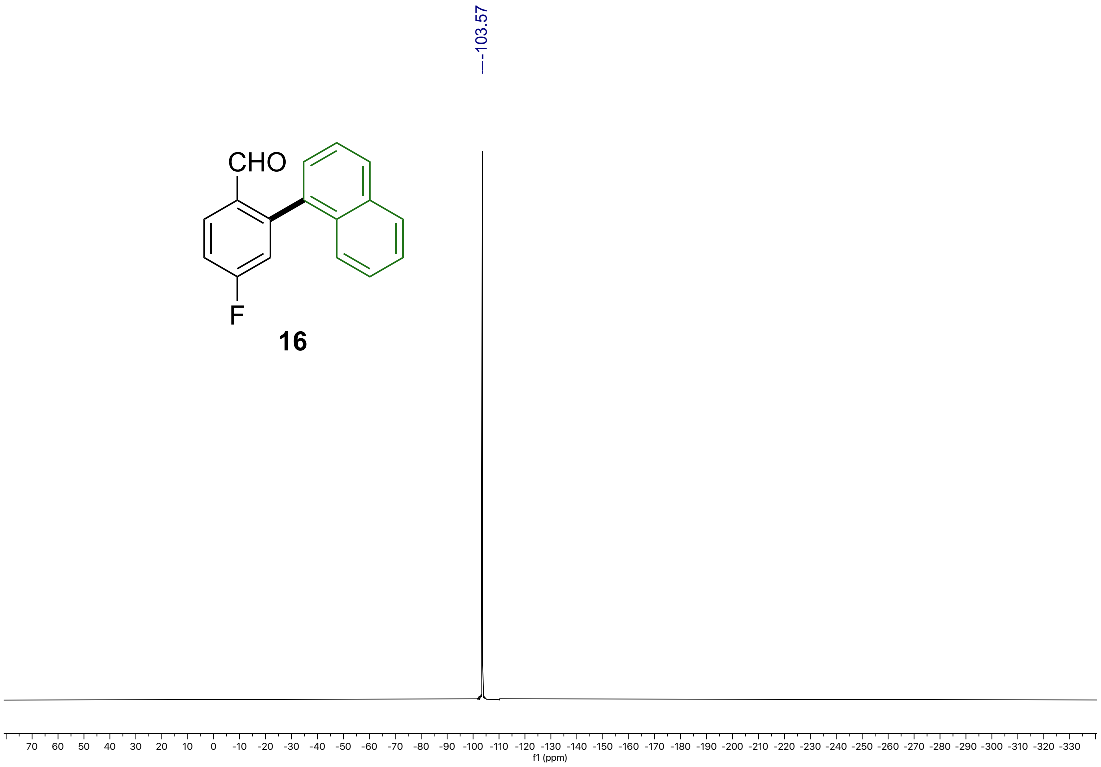
-103.57
CHO
F
f1 (ppm)

16

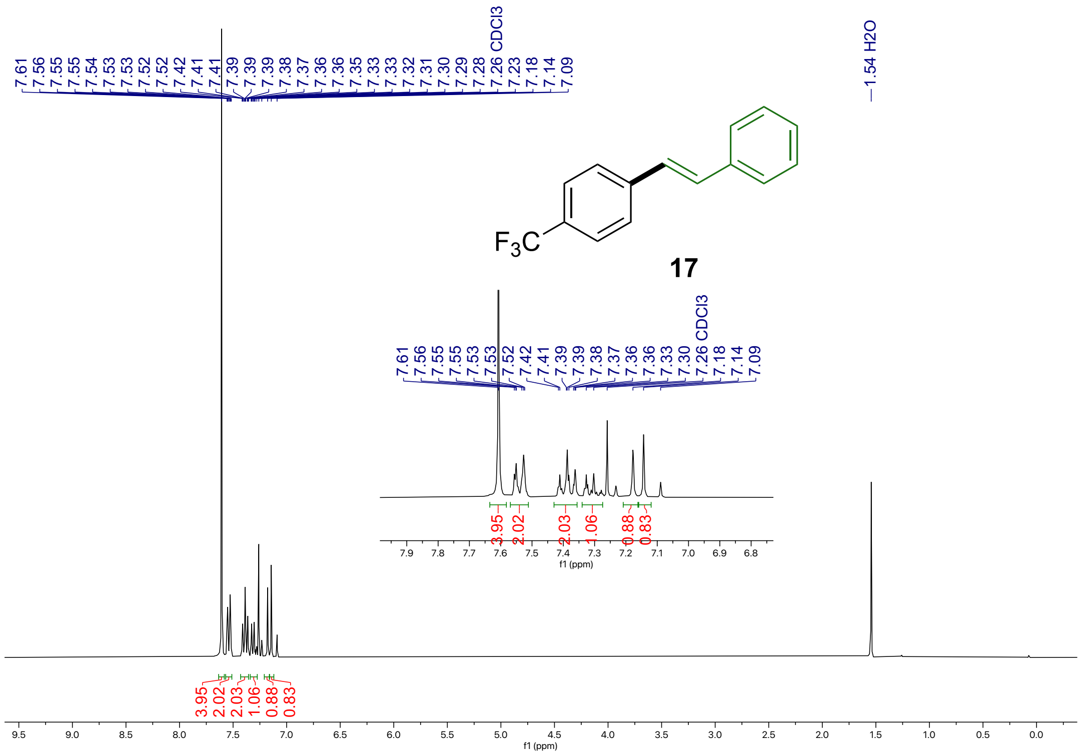
7.61 7.56 7.55 7.55 7.54 7.53 7.53 7.52 7.52 7.42 7.41 7.41 7.39 7.39 7.39 7.38 7.37 7.36 7.36 7.35 7.33 7.33 7.32 7.31 7.30 7.29 7.28 7.26 CDCl3 7.23 7.18 7.14 7.09
1.54 H2O
F3C
3.95 2.02 2.03 1.06 0.88 0.83
f1 (ppm)

17

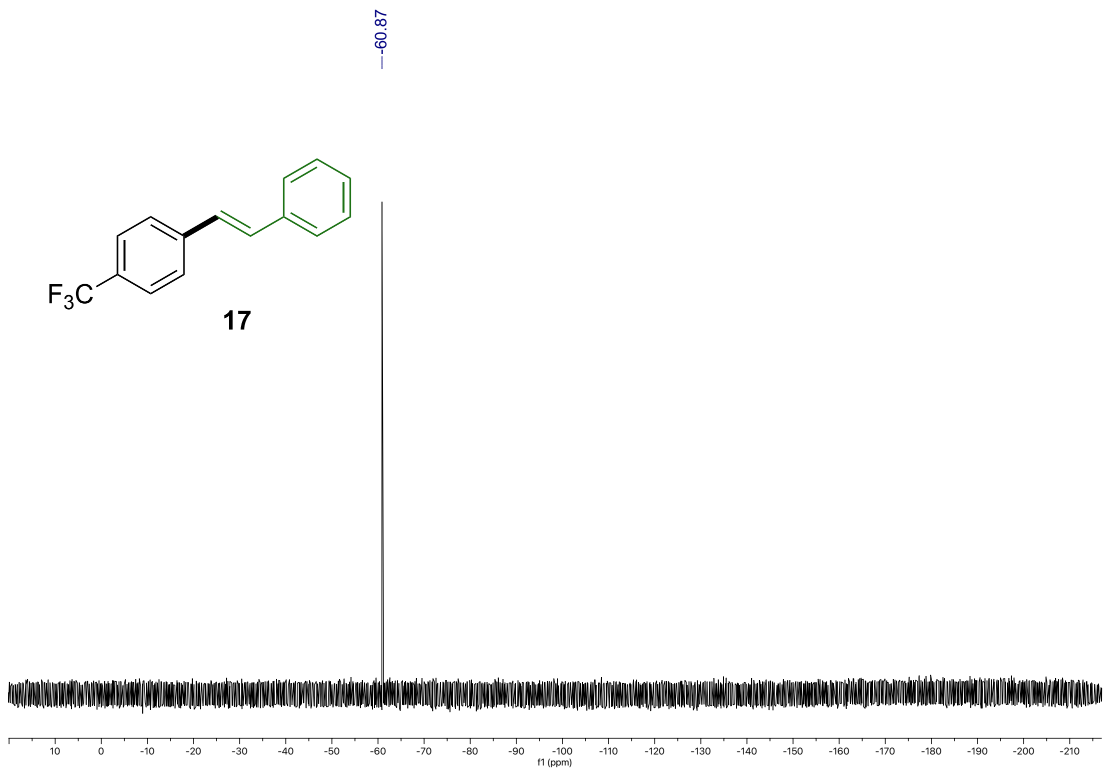
-60.87
F3C
17
10 0 -10 -20 -30 -40 -50 -60 -70 -80 -90 -100 -110 -120 -130 -140 -150 -160 -170 -180 -190 -200 -210
f1 (ppm)

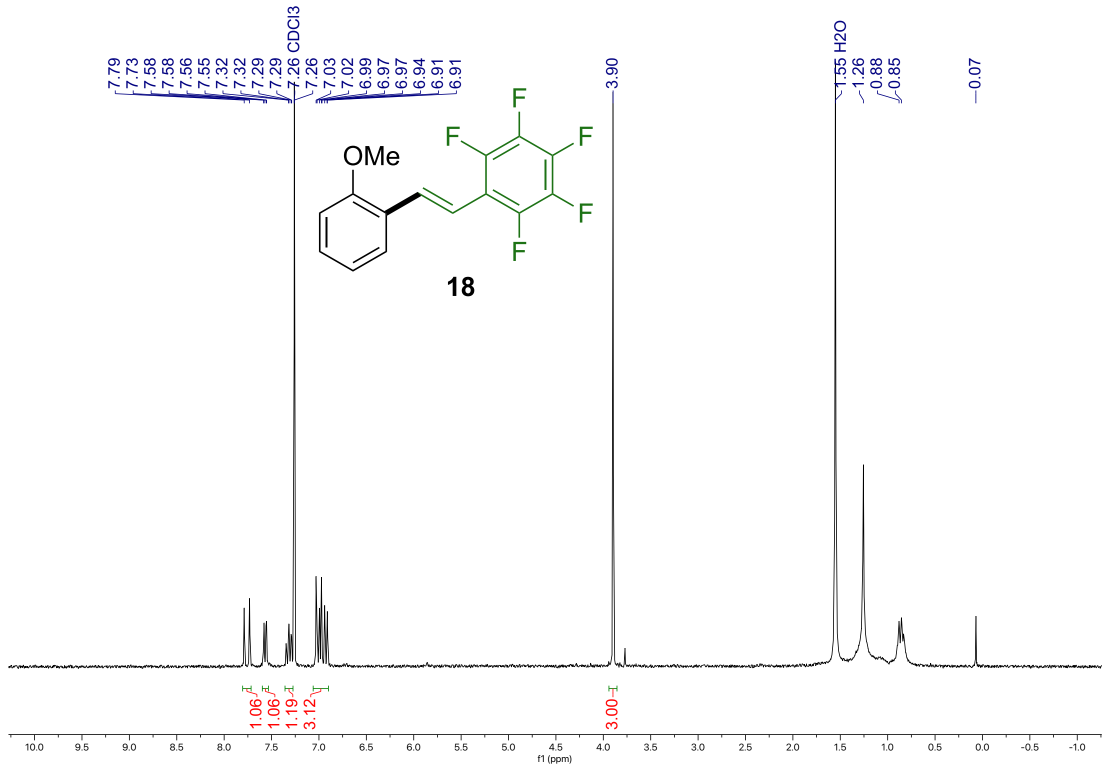
7.79 7.73 7.58 7.56 7.55 7.32 7.32 7.29 7.29 7.26 CDCl3 7.26 7.03 7.02 6.99 6.97 6.97 6.94 6.91 6.91
3.90
1.55 H2O 1.26 0.88 0.85
0.07
OMe
F F F F F
18
1.06 1.06 1.19 3.12 3.00
10.0 9.5 9.0 8.5 8.0 7.5 7.0 6.5 6.0 5.5 5.0 4.5 4.0 3.5 3.0 2.5 2.0 1.5 1.0 0.5 0.0 -0.5 -1.0
f1 (ppm)

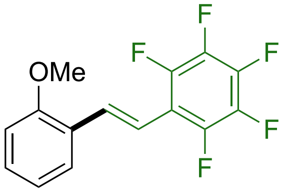
OMe
F
F
F
F
F

18

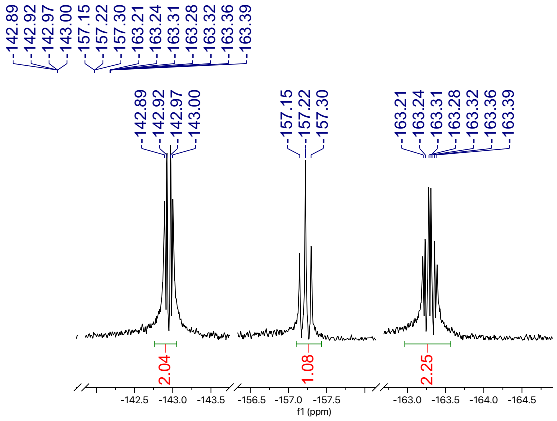
-142.89 -142.92 -142.97 -143.00 -157.15 -157.22 -157.30 -163.21 -163.24 -163.31 -163.28 -163.32 -163.36 -163.39
2.04
1.08
2.25
-142.5 -143.0 -143.5 -156.5 -157.0 -157.5 -163.0 -163.5 -164.0 -164.5
f1 (ppm)

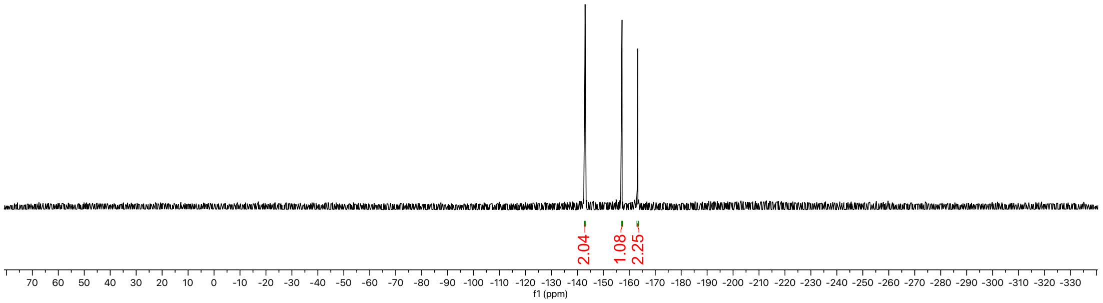
2.04
1.08
2.25
70 60 50 40 30 20 10 0 -10 -20 -30 -40 -50 -60 -70 -80 -90 -100 -110 -120 -130 -140 -150 -160 -170 -180 -190 -200 -210 -220 -230 -240 -250 -260 -270 -280 -290 -300 -310 -320 -330
f1 (ppm)

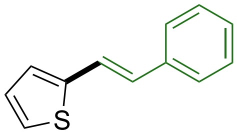
S

19

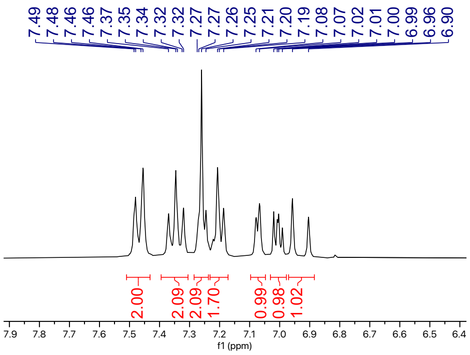
7.49 7.48 7.46 7.37 7.35 7.34 7.32 7.32 7.27 7.27 7.26 7.25 7.21 7.20 7.19 7.08 7.07 7.02 7.01 7.00 6.99 6.96 6.90
2.00
2.09
2.09
1.70
0.99
0.98
1.02
7.9 7.8 7.7 7.6 7.5 7.4 7.3 7.2 7.1 7.0 6.9 6.8 6.7 6.6 6.5 6.4
f1 (ppm)

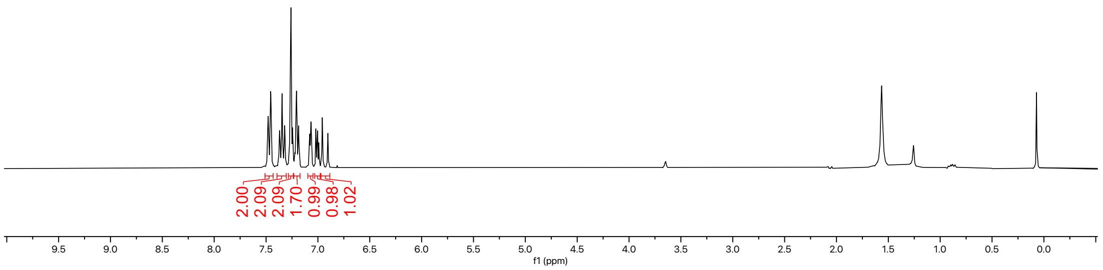
7.49 7.48 7.47 7.46 7.37 7.46 7.36 7.34 7.35 7.32 7.32 7.27 7.27 7.25 7.26 7.21 7.20 7.19 7.08 7.07 7.01 7.02 7.00 6.99 6.96 6.90
2.00
2.09
2.09
1.70
0.99
0.98
1.02
9.5 9.0 8.5 8.0 7.5 7.0 6.5 6.0 5.5 5.0 4.5 4.0 3.5 3.0 2.5 2.0 1.5 1.0 0.5 0.0
f1 (ppm)

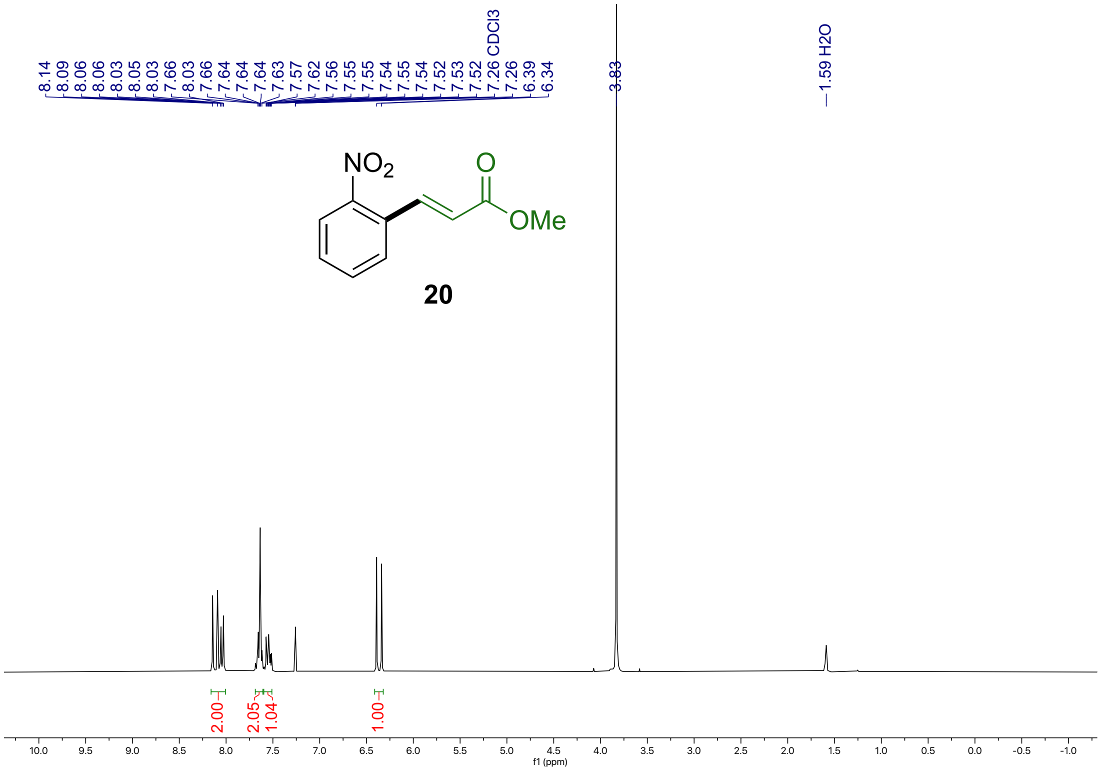

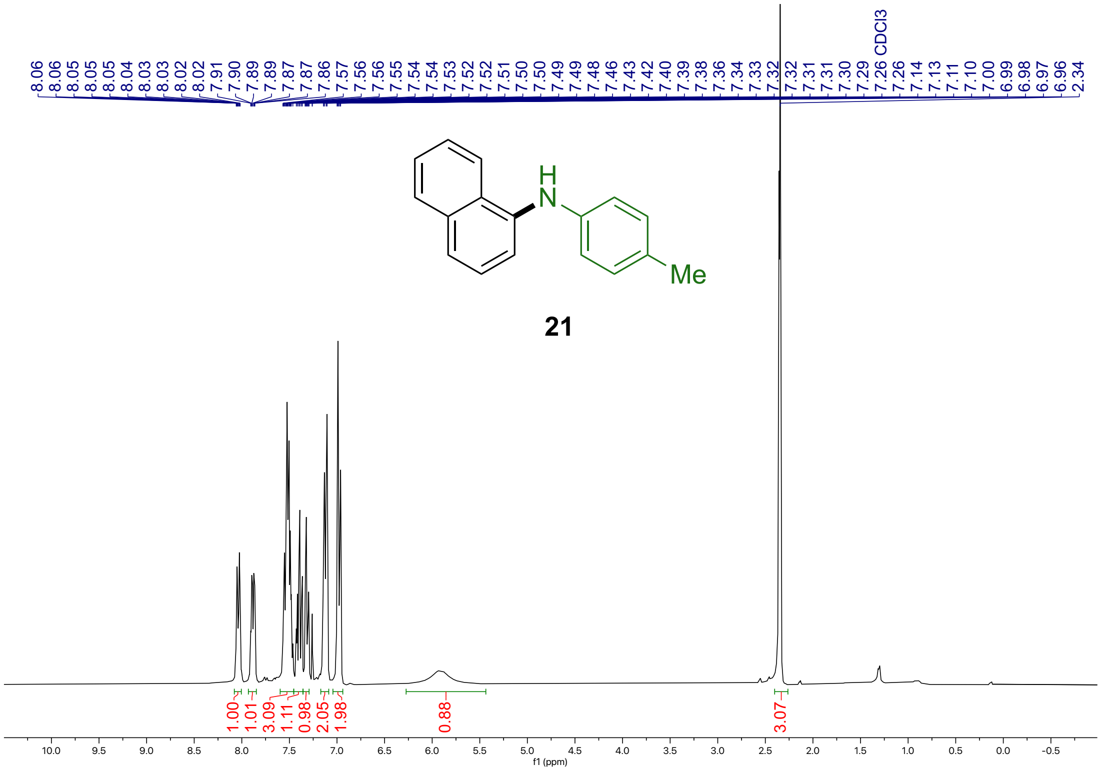

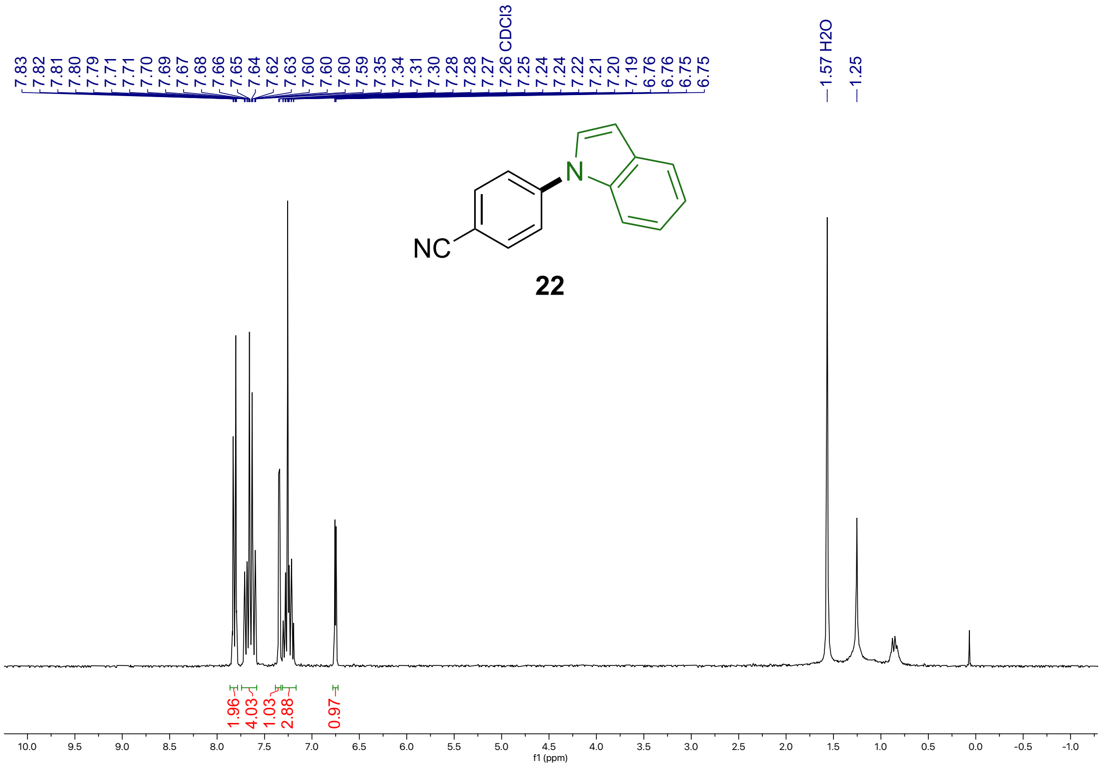

7.83 7.82 7.81 7.80 7.79 7.71 7.71 7.70 7.69 7.67 7.68 7.66 7.65 7.64 7.62 7.63 7.60 7.60 7.59 7.35 7.34 7.31 7.30 7.28 7.28 7.27 7.26 CDCl3 7.25 7.24 7.24 7.22 7.21 7.20 7.19 6.76 6.75 6.75
1.57 H2O
1.25
NC
N
22
1.96 4.03 1.03 2.88 0.97
f1 (ppm)

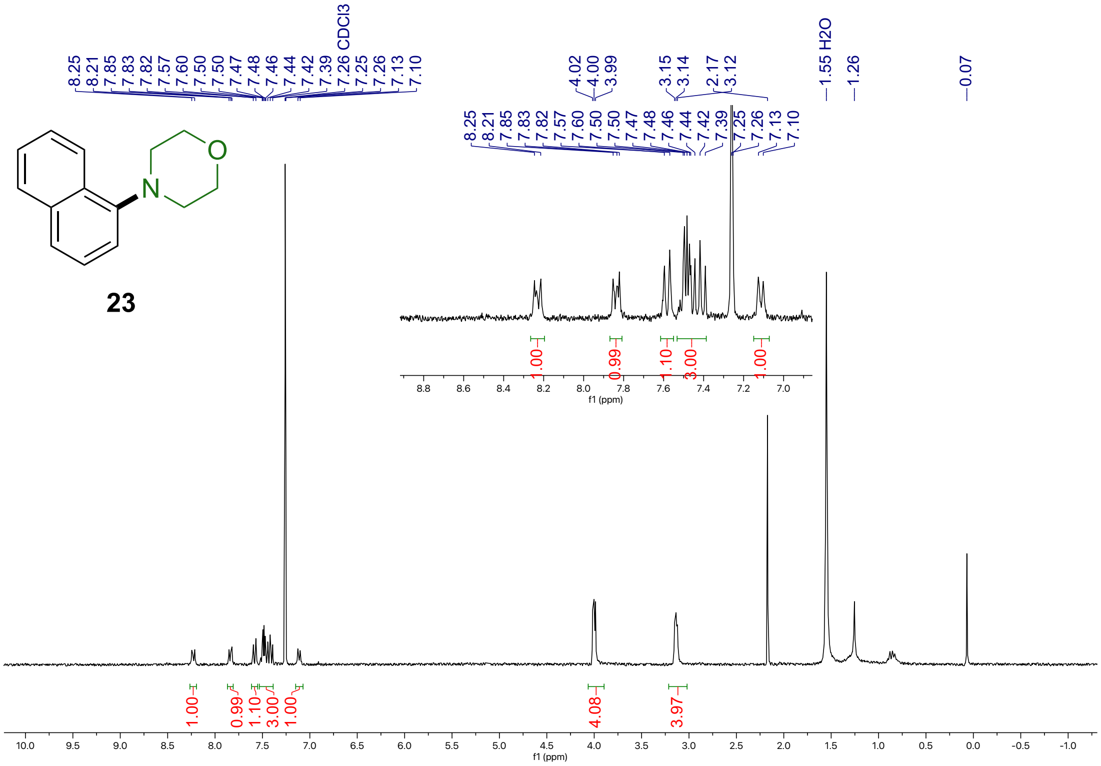

8.25 8.21 7.85 7.83 7.82 7.57 7.60 7.50 7.50 7.47 7.48 7.46 7.44 7.42 7.39 7.26 CDCl3 7.25 7.26 7.13 7.10
4.02 4.00 3.99
3.15 3.14 2.17 3.12
1.55 H2O
1.26
0.07
N
O
23
1.00 0.99 1.10 3.00 1.00
4.08 3.97
f1 (ppm)

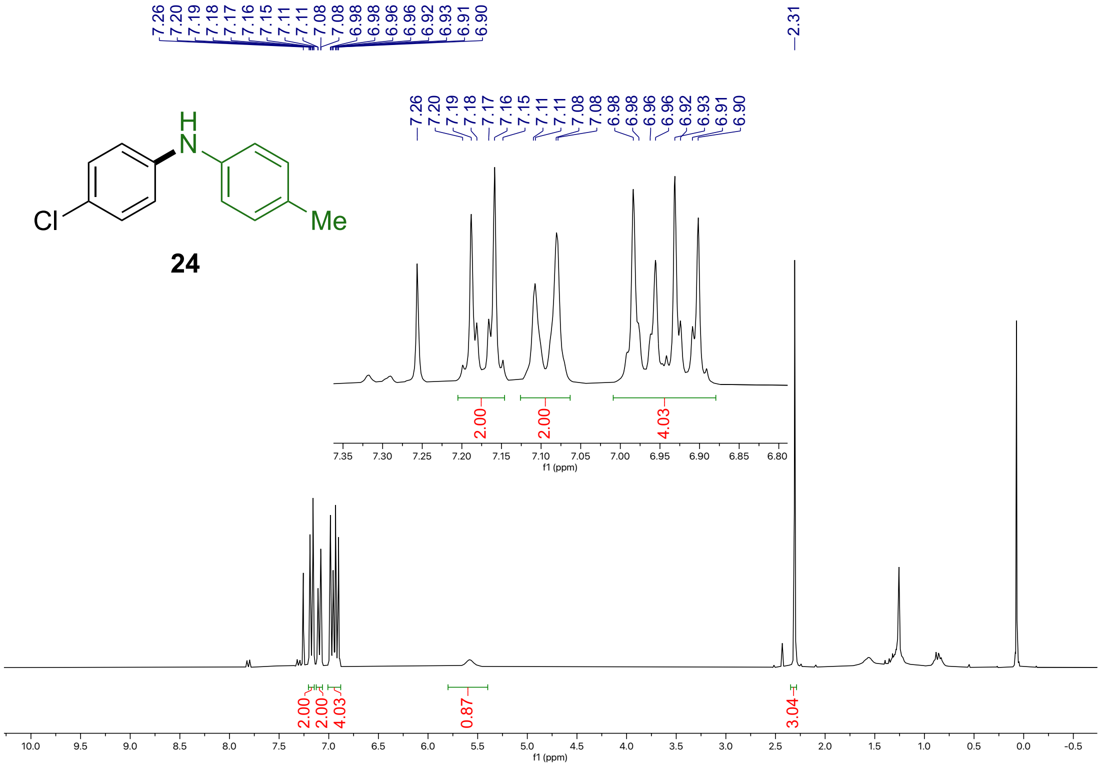

7.26 7.20 7.19 7.18 7.17 7.16 7.15 7.11 7.11 7.08 7.08 6.98 6.98 6.96 6.96 6.92 6.93 6.91 6.90
2.31
Cl
H
N
Me
24
2.00
2.00
4.03
0.87
3.04
f1 (ppm)

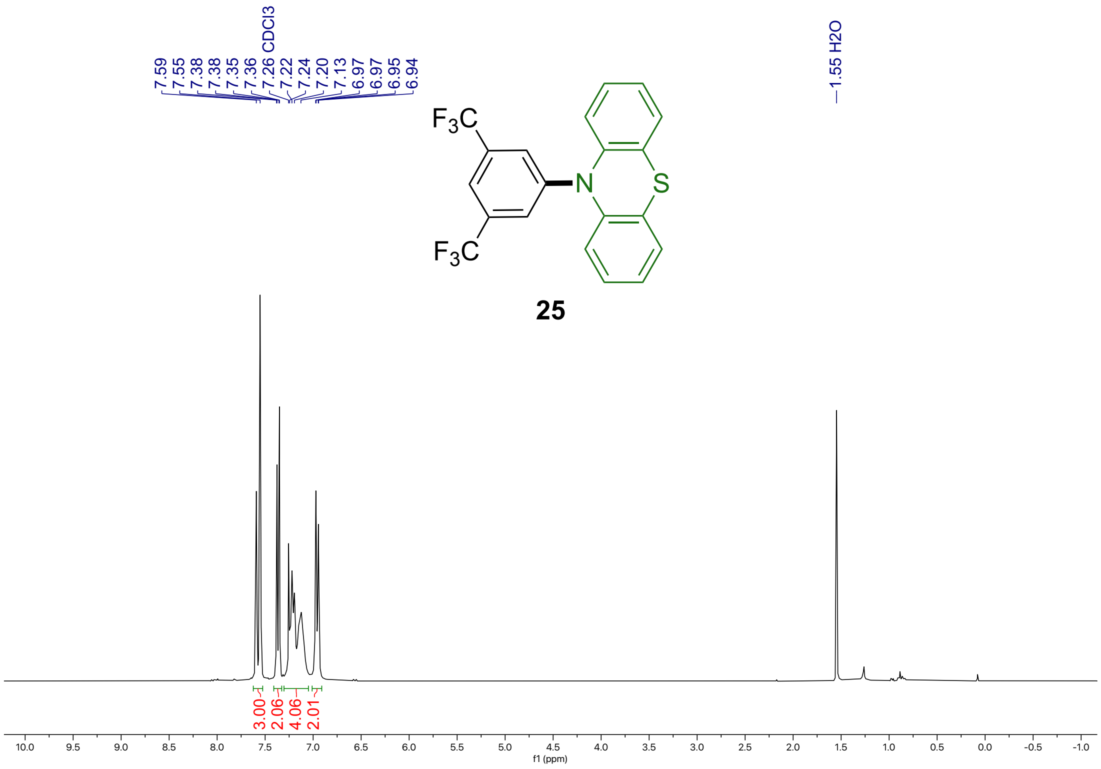

7.59 7.55 7.38 7.38 7.35 7.36 7.26 CDCl3 7.22 7.24 7.20 7.13 6.97 6.97 6.95 6.94
1.55 H2O
F3C
N
S
F3C
25
3.00
2.06
4.06
2.01
f1 (ppm)

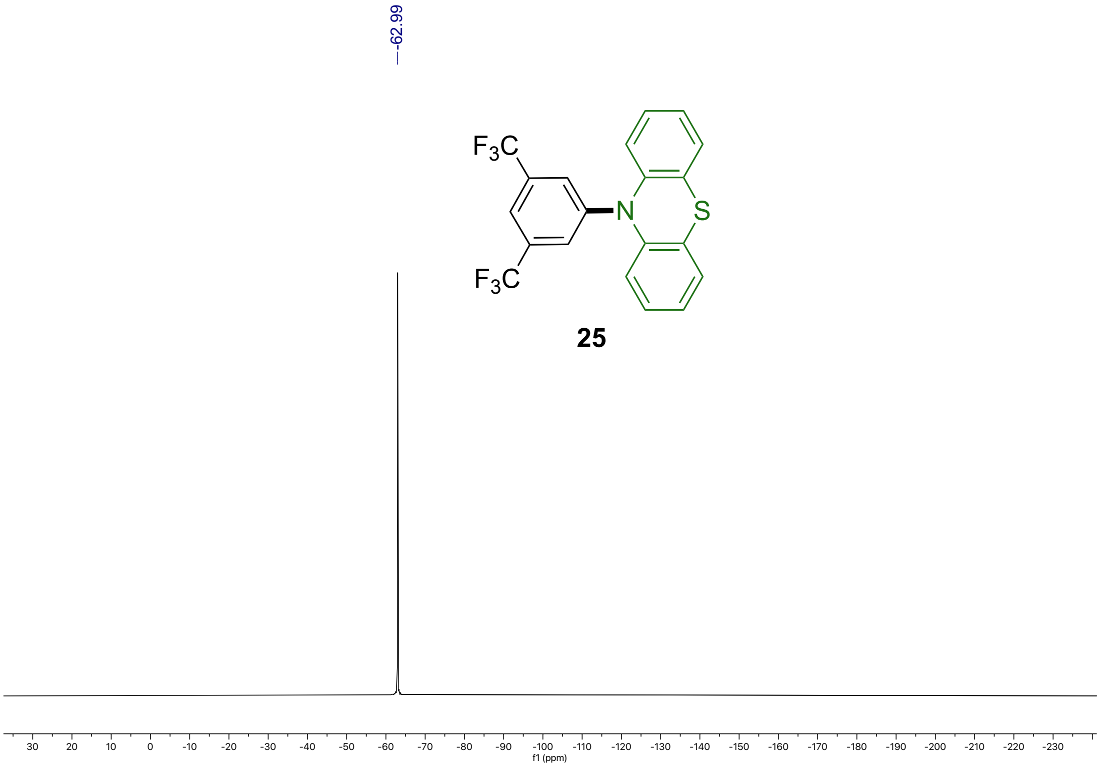
-62.99
F3C
N
S
F3C
30 20 10 0 -10 -20 -30 -40 -50 -60 -70 -80 -90 -100 -110 -120 -130 -140 -150 -160 -170 -180 -190 -200 -210 -220 -230
f1 (ppm)

25

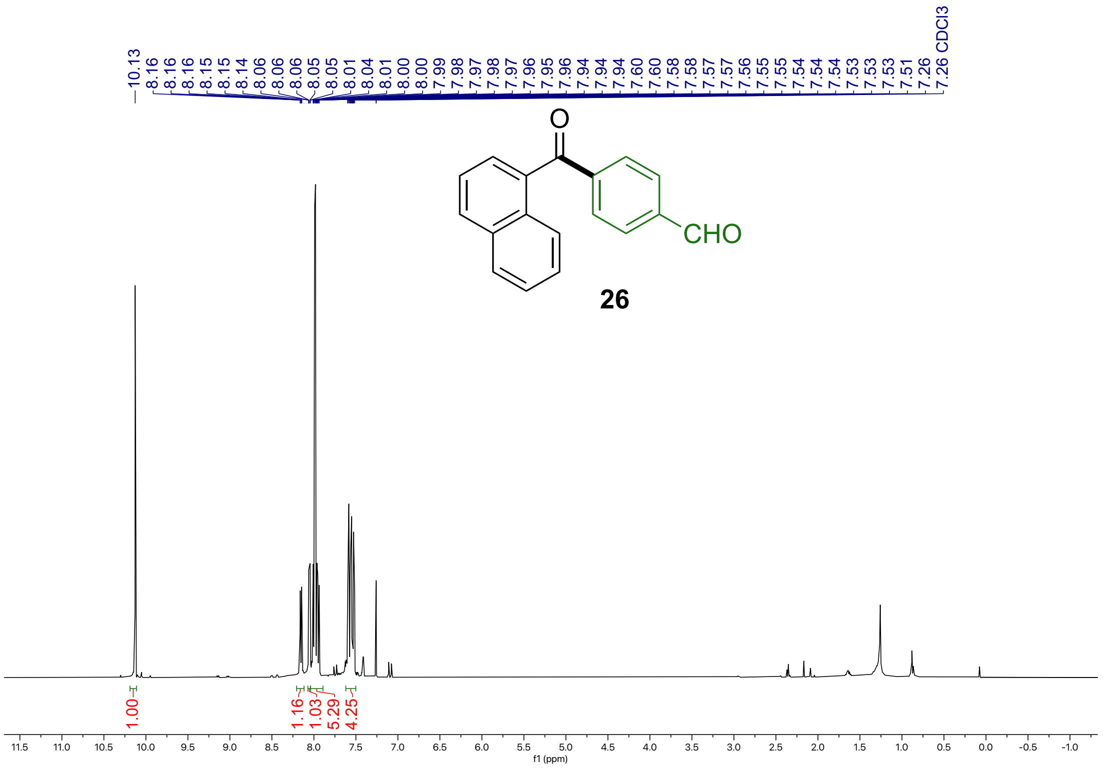
10.13
8.16 8.16 8.16 8.15 8.15 8.14 8.06 8.06 8.05 8.05 8.04 8.01 8.01 8.00 7.99 7.98 7.97 7.98 7.97 7.96 7.95 7.96 7.94 7.94 7.94 7.60 7.60 7.58 7.58 7.57 7.57 7.56 7.55 7.55 7.54 7.54 7.54 7.53 7.53 7.53 7.51 7.26 7.26 CDCl3
O
CHO
1.00
1.16
1.03
5.29
4.25
11.5 11.0 10.5 10.0 9.5 9.0 8.5 8.0 7.5 7.0 6.5 6.0 5.5 5.0 4.5 4.0 3.5 3.0 2.5 2.0 1.5 1.0 0.5 0.0 -0.5 -1.0
f1 (ppm)

26

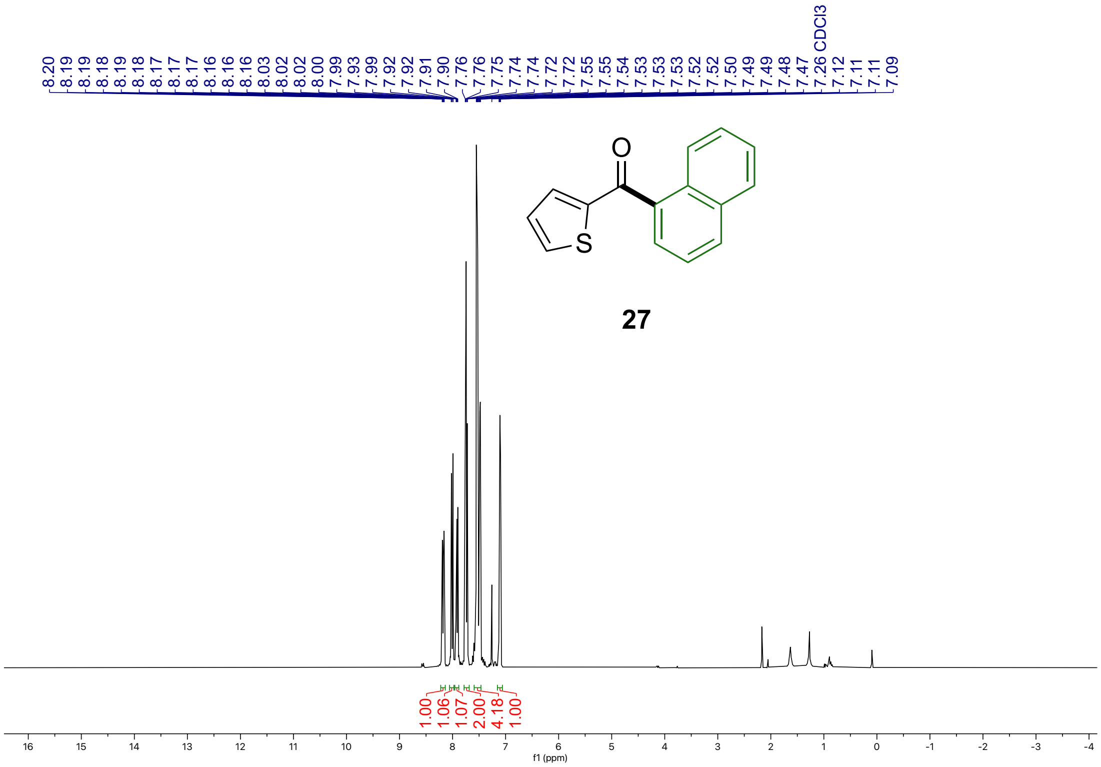
8.20
8.19
8.18
8.19
8.18
8.17
8.17
8.16
8.16
8.03
8.02
8.00
7.99
7.93
7.99
7.92
7.92
7.91
7.90
7.76
7.76
7.75
7.74
7.74
7.72
7.55
7.55
7.54
7.53
7.53
7.53
7.52
7.52
7.50
7.49
7.49
7.48
7.47
7.26 CDCl3
7.12
7.11
7.11
7.09
O
S
27
1.00
1.06
1.07
2.00
4.18
1.00
f1 (ppm)

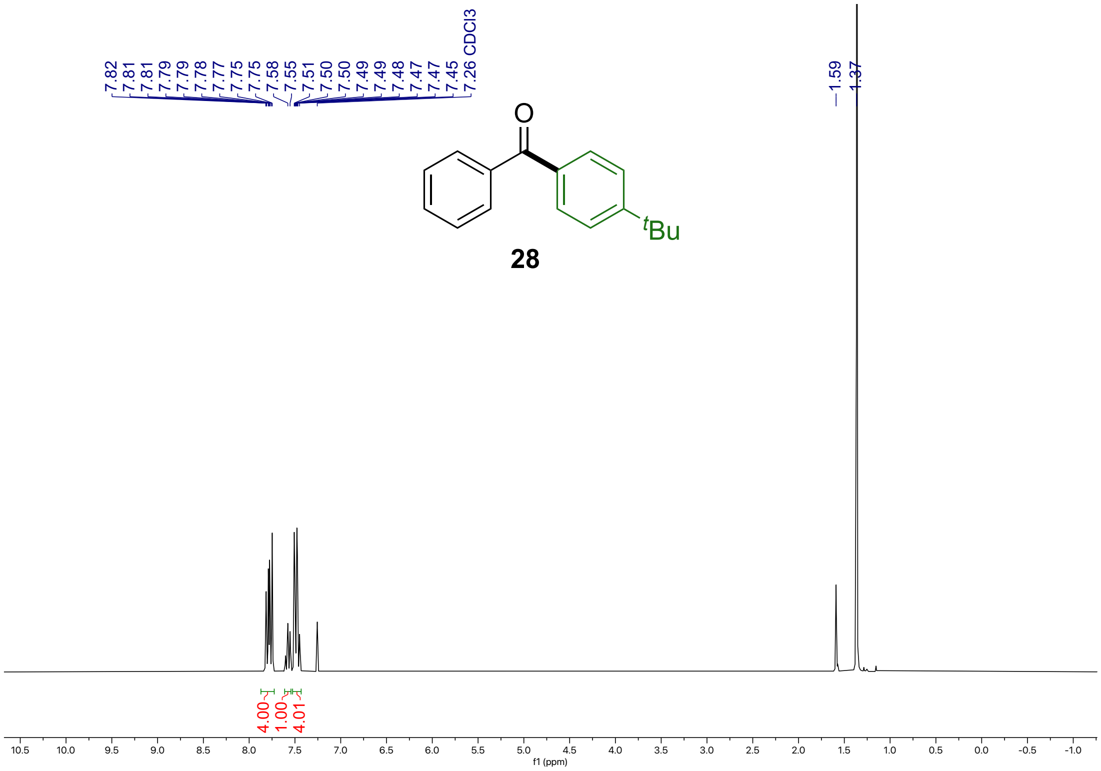
7.82
7.81
7.81
7.79
7.79
7.78
7.77
7.75
7.75
7.58
7.55
7.51
7.50
7.49
7.49
7.48
7.47
7.47
7.45
7.26 CDCl3
1.59
1.37
O
tBu
28
4.00
1.00
4.01
f1 (ppm)

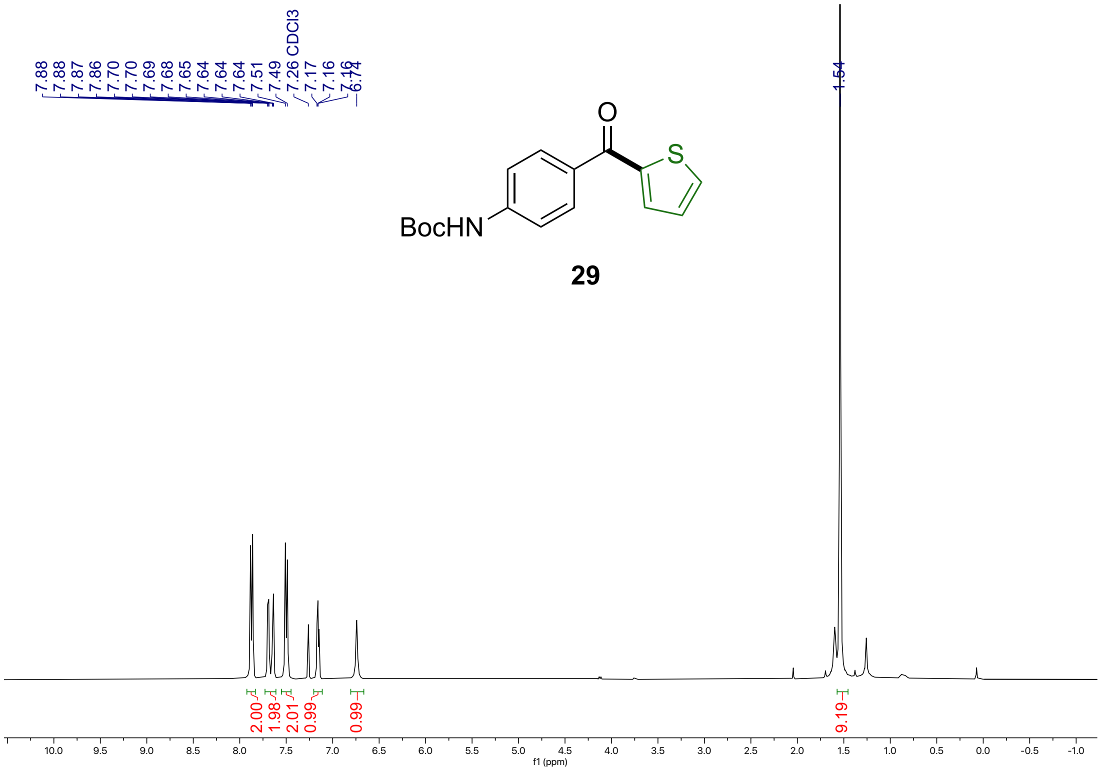

29

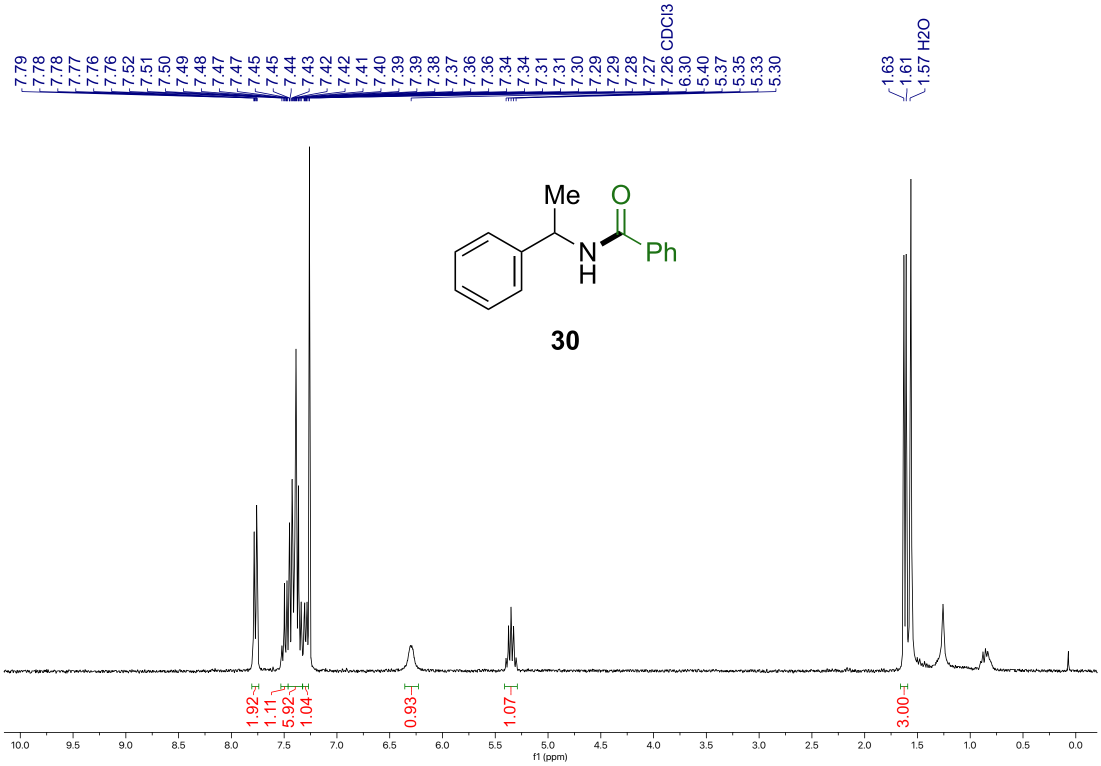

30

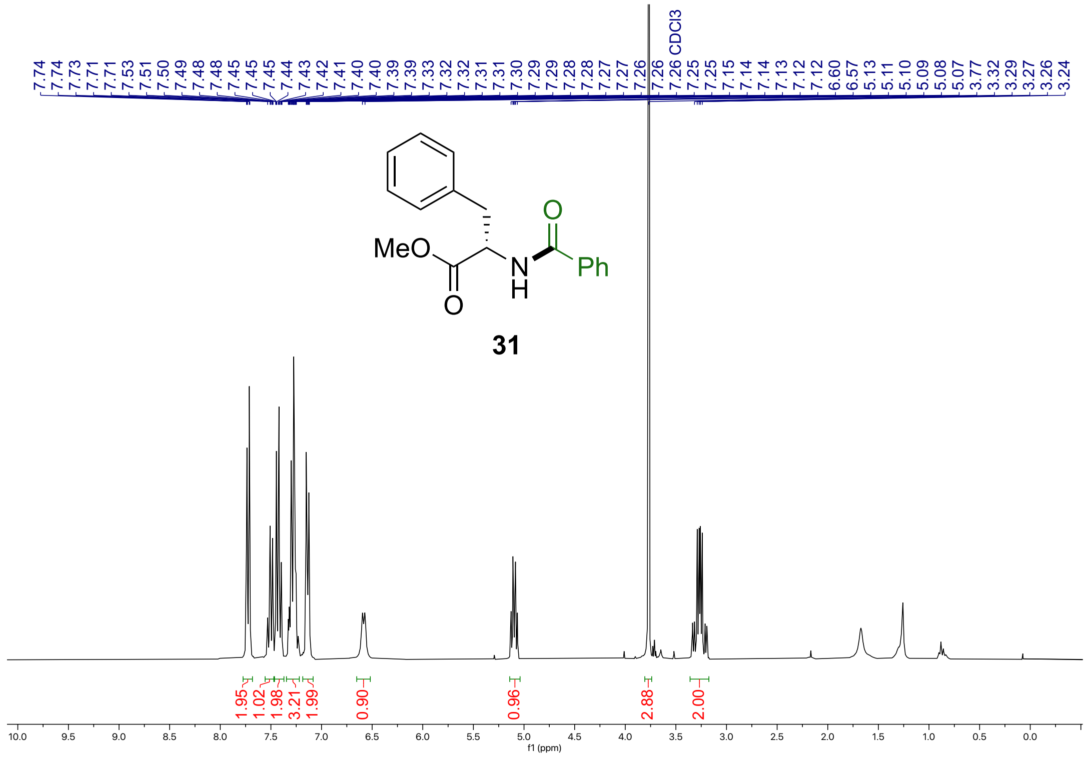

31

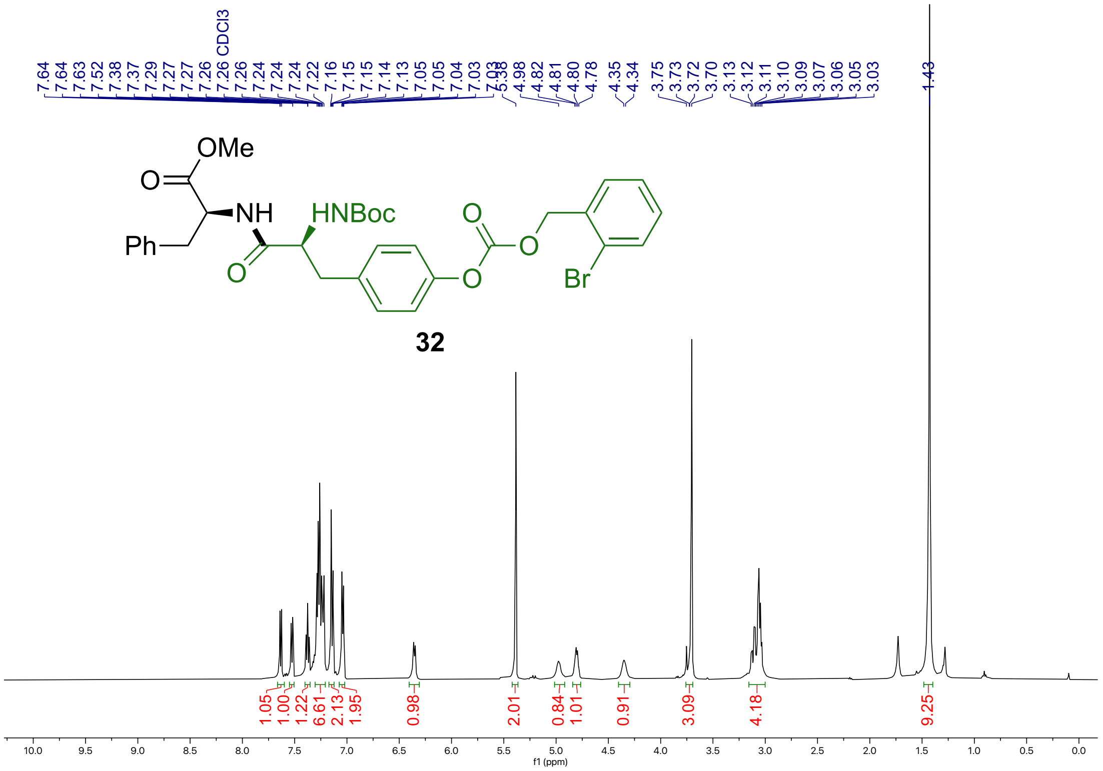

32

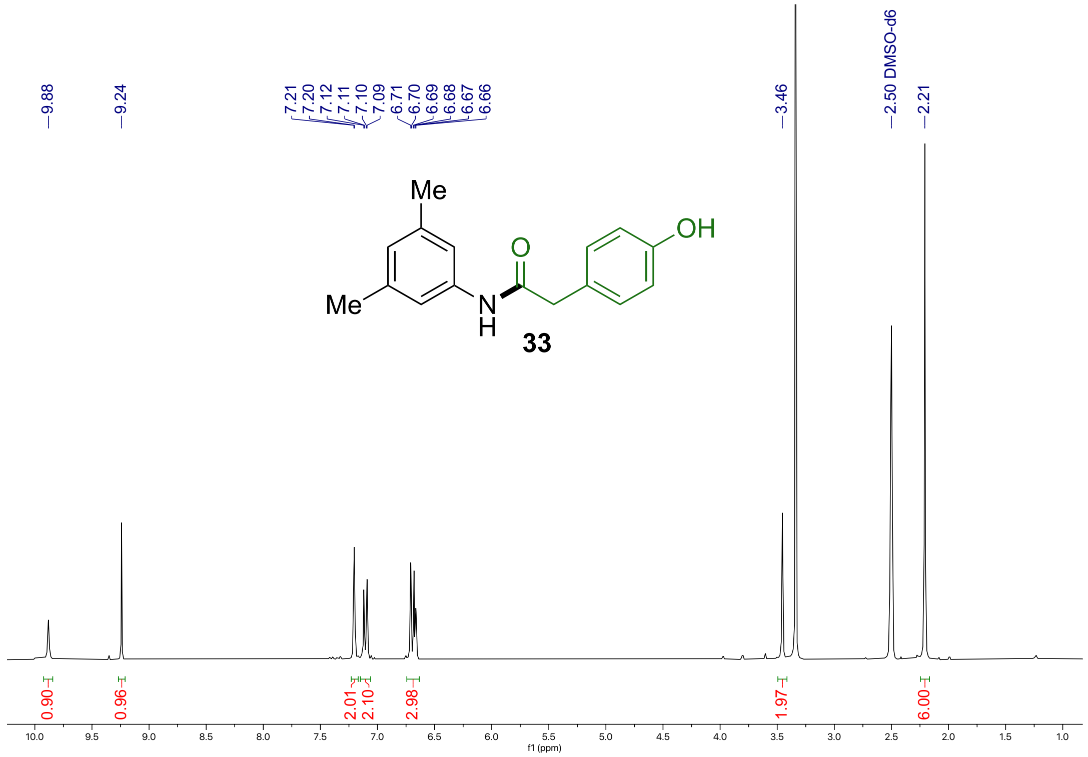
9.88
9.24
7.21
7.20
7.12
7.11
7.10
7.09
6.71
6.70
6.69
6.68
6.67
6.66
3.46
2.50 DMSO-d6
2.21
Me
O
OH
Me
N
H
33
0.90
0.96
2.01
2.10
2.98
1.97
6.00
10.0 9.5 9.0 8.5 8.0 7.5 7.0 6.5 6.0 5.5 5.0 4.5 4.0 3.5 3.0 2.5 2.0 1.5 1.0
f1 (ppm)

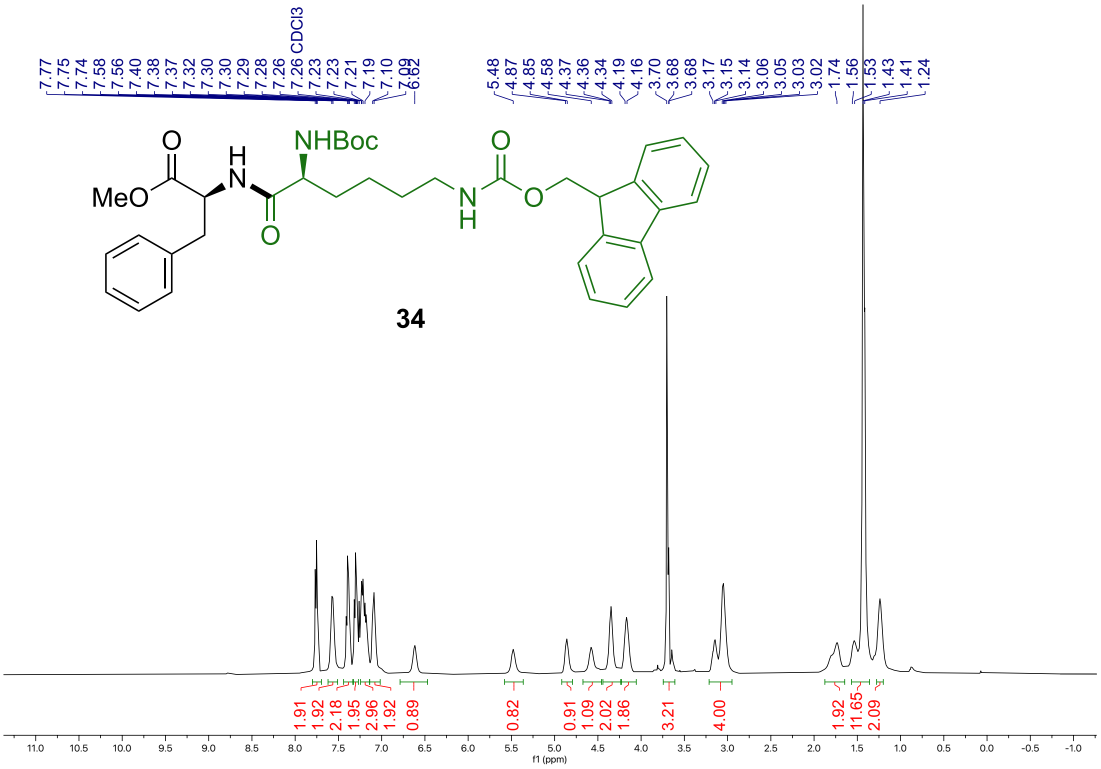
7.77
7.75
7.74
7.58
7.56
7.40
7.38
7.37
7.32
7.30
7.30
7.29
7.28
7.26
7.26 CDCl3
7.23
7.23
7.21
7.19
7.10
7.09
6.62
5.48
4.87
4.85
4.58
4.37
4.36
4.34
4.19
4.16
3.70
3.68
3.68
3.17
3.15
3.14
3.06
3.05
3.03
3.02
1.74
1.56
1.53
1.43
1.41
1.24
O
MeO
H
N
NHBoc
O
O
N
H
O
34
1.91
1.92
2.18
1.95
2.96
1.92
0.89
0.82
0.91
1.09
2.02
1.86
3.21
4.00
1.92
11.65
2.09
11.0 10.5 10.0 9.5 9.0 8.5 8.0 7.5 7.0 6.5 6.0 5.5 5.0 4.5 4.0 3.5 3.0 2.5 2.0 1.5 1.0 0.5 0.0 -0.5 -1.0
f1 (ppm)
